# RiceMetaSysB: a database of blast and bacterial blight responsive genes in rice and its utilization in identifying key blast-resistant WRKY genes

**DOI:** 10.1093/database/baz015

**Published:** 2019-02-11

**Authors:** V Sureshkumar, Bipratip Dutta, Vishesh Kumar, G Prakash, Dwijesh C Mishra, K K Chaturvedi, Anil Rai, Amitha Mithra Sevanthi, Amolkumar U Solanke

**Affiliations:** 1 Indian Council of Agricultural Research (ICAR)—National Research Centre on Plant Biotechnology, Pusa Campus, New Delhi, India; 2 Jamia Hamdard, Hamdard Nagar, New Delhi, India; 3 ICAR—Indian Agricultural Research Institute, Pusa Campus, New Delhi, India; 4 ICAR—Indian Agricultural Statistics Research Institute, Pusa Campus, New Delhi, India

## Abstract

Nearly two decades of revolution in the area of genomics serves as the basis of present-day molecular breeding in major food crops such as rice. Here we report an open source database on two major biotic stresses of rice, named RiceMetaSysB, which provides detailed information about rice blast and bacterial blight (BB) responsive genes (RGs). Meta-analysis of microarray data from different blast- and BB-related experiments across 241 and 186 samples identified 15135 unique genes for blast and 7475 for BB. A total of 9365 and 5375 simple sequence repeats (SSRs) in blast and BB RGs were identified for marker development. Retrieval of candidate genes using different search options like genotypes, tissue, developmental stage of the host, strain, hours/days post-inoculation, physical position and SSR marker information is facilitated in the database. Search options like ‘common genes among varieties’ and ‘strains’ have been enabled to identify robust candidate genes. A 2D representation of the data can be used to compare expression profiles across genes, genotypes and strains. To demonstrate the utility of this database, we queried for blast-responsive WRKY genes (fold change ≥5) using their gene IDs. The structural variations in the 12 WRKY genes so identified and their promoter regions were explored in two rice genotypes contrasting for their reaction to blast infection. Expression analysis of these genes in panicle tissue infected with a virulent and an avirulent strain of *Magnaporthe oryzae* could identify *WRKY7*, *WRKY58*, *WRKY62*, *WRKY64* and *WRKY76* as potential candidate genes for resistance to panicle blast, as they showed higher expression only in the resistant genotype against the virulent strain. Thus, we demonstrated that RiceMetaSysB can play an important role in providing robust candidate genes for rice blast and BB.

## Introduction

Rice (*Oryza sativa* L.) remains a staple food crop for more than half of the world’s population, while it is the means of livelihood for millions of poor farmers in South East Asia. Therefore, production and productivity of rice have a huge impact on world peace and economy. Still, every year a large amount of rice produce is ruined by several biotic and abiotic stresses undermining sustainable rice production. The major abiotic stresses that affect rice production are drought, heat, cold, salinity, flooding and high or low light intensity, while the major biotic stresses are diseases caused by bacteria, fungi, viruses, nematodes and infestation by insect pests. Among the various pathogens affecting the rice crop, the most important ones are *Magnaporthe oryzae* causing rice blast disease and *Xanthomonas oryzae* pv. *oryzae* (Xoo) causing bacterial blight (BB) (1–3). *M. oryzae* is a part of a species complex and causes disease on various grasses and other related species, such as barley, wheat and finger millet (4). It is a highly adaptive pathogen that can affect plants across all developmental stages and tissues, but the leaf and panicle blast cause huge yield losses, sometimes up to 100% (5). The causal organism of BB Xoo is highly specific to rice plant and can reduce crop production up to 70% under severe epidemics (6–8). This is a vascular pathogen and enters the plant via hydathodes and wounds. First, it reproduces in the intercellular space beneath epithelial cells and then spreads via xylem tissue leading to the disease (9). The best way to tackle these diseases is the use of resistant cultivars with one or more R genes. In rice, till date more than 100 R genes providing genetic resistance to rice blast have been identified, of which 24 have been cloned and validated. All these R genes are of Nucleotide binding site-leucine rich repeat (NBS-LRR) type except one (10–11). Similarly, 41 R genes (i.e. 29 dominant and 12 recessive) conferring resistance to BB have so far been identified based on disease responses to different Xoo races (12).

Transcriptome studies provide an important means to study the functional elements involved in stress response, which ultimately reveal the transcriptional regulation of genes under different growth stages, and tissues (13). Expression studies on rice lines carrying different blast resistance genes (*Pia, Pish, Pi9, Pi1* and *Pi54*) revealed the role of a plethora of transcriptional regulons acting through multiple signaling kinases and transcription factors, upon infection by different *Magnaporthe* strains (14–20). Likewise, many transcriptomes studies of R gene-introgressed BB-resistant lines were generated to understand the resistant gene networks under disease infection (21–23). The major regulons identified from such studies represented disease resistance genes, pathogenesis-related proteins, enzymes of oxidative burst and LRR domains, signaling molecules, transcription factors (MYB, ERF, WRKY etc.) and cell wall modification-related genes.

With the accumulation of a large number of genome-wide gene expression data sets in the public domain, different databases were developed in rice on either a specific stress or all stresses like RiceSRTFDB for salinity stress (http://www.nipgr.res.in/RiceSRTFDB.html), drought stress gene database (http://pgsb.helmholtz-muenchen.de/droughtdb/) and plant stress gene database (http://ccbb.jnu.ac.in/stressgenes/). Recently, we have also developed RiceMetaSys database for identification and analysis of differentially expressed genes (DEGs) under drought and salt stresses in rice (24). All these databases catered to one or more abiotic stresses. Thus, there is no database that comprehensively covers gene expression profiles of major rice diseases across pathogen strains and host genotypes. Even those databases, which cater to all stresses and developmental stages, such as OryzaExpress, RicePLEX and RiceXPro, did not allow meta-analysis of multiple expression data sets under one common theme (24). Though ROAD database allowed meta-analysis, it is no more functional (24). Moreover, in ROAD one had to search for data sets for meta-analysis and carry out the same afresh. Hence, in the present study we have developed a database, entitled RiceMetaSysB (B for biotic stress), on rice blast and BB responsive genes (RGs) using meta-analysis of publicly available microarray experiments. We have further demonstrated the utility of our database by characterizing a major transcription factor governing plant disease reactions, WRKY, under panicle blast infection in resistant and susceptible rice varieties infected with virulent and avirulent strains of the blast pathogen.

## Database construction and content

### Data source

Expression microarrays are useful for identifying genes specific to different tissues and environmental conditions. Affymetrix and Agilent microarrays are the two major microarray technologies used for gene expression studies. Affymetrix arrays are single-channel systems while Agilent has both single- and two-channel microarray systems. To construct RiceMetaSysB, data from 241 and 186 samples on rice blast and BB stress were collected from NCBI GEO (Gene Expression Omnibus) database (https://www.ncbi.nlm.nih.gov/geo), respectively. Expression data for rice blast stress were obtained from nine genotypes {Nipponbare, Nipponbare [Pia], Nipponbare [Pish], Lijiangxintuanheigu (LTH), IRBL18 [Pi9 near isogenic line (NIL) of LTH], IRBL22 [Pi9 NIL of LTH], Taipei 309, transgenic lines of TP309:OX-Pi54 and OX-OsWRKY28} while that of BB were from five genotypes (Nipponbare, IR24, IRBB5, IRBB7 and Osaba1-1), representing different strains, inoculation time points, growth stages and tissues. Detailed information of the data sets is provided in Supplementary data Tables S1 and S2.

### Meta-analysis of gene expression arrays

For uniformity of the data sets that were generated across experiments in different laboratories, preprocessing (background correction) was carried out for all data sets. Preprocessing was done using robust multi-array average method for Affymetrix data sets (25). For single-channel microarray Agilent data sets, normalization was done using quantile method while for two-channel microarray systems, normalization was done using aquantile method (26). Limma package version 3.28.21 from R package was used for gene expression analysis (27). For filtering the robust candidate genes, we have used parameters adjusted *P*-value <0.05, average expression >8 and log2FC <−1 or >1 for both the biotic stresses. Data processing workflow was as per Sandhu *et al.* (24) and is given in Supplementary data Figure S1.

### Database construction

Affymetrix IDs were converted into MSU and Rice Annotation Project (RAP) IDs by using OryzaExpress (http://bioinf.mind.meiji.ac.jp/OryzaExpress/ID_converter.php). Physical position and annotations of the identified biotic RGs were retrieved from RGAP (Rice Genome Annotation Project; http://rice.plantbiology.msu.edu/) and RAP (https://rapdb.dna.affrc.go.jp/) databases. BatchPrimer3 tool was used both for identification of microsatellites and designing primers for their amplification (28). RiceMetaSysB database was hosted under XAMPP (Apache, MariaDB, PHP and Perl) server in the Windows operating system. PHP5 and HTML5 were used for constructing the front end while MySQL was used at the back end. CSS, Jquery and JS were used for providing a better interface of the database. Chart.js was used to generate interactive graphs of the expression profiles of DEGs across/within genotypes and strains. The URL of the database is http://14.139.229.201/ricemetasysb/.

### Differentially expressed WRKY gene search for rice blast from database

We used MSU IDs of all WRKY genes as input to retrieve the differentially expressed (DE) WRKY genes using ‘LOC-ID search’ option under rice (leaf) blast RGs. The output was downloaded in excel format and rearranged on the basis of log fold change (FC) values. For further validation by expression analysis, those WRKY genes that had a significant expression value of logFC ≥5 and common with the panicle blast-specific WRKY genes identified from the transcriptome data generated in our laboratory using a resistant genotype Tetep and susceptible genotype HP2216 (unpublished data) were selected.

### Analysis of structural variations in WRKY genes

We had earlier generated low coverage whole genome sequence (WGS) of rice genotypes Tetep and HP2216 in our laboratory. We used this WGS data to identify structural variations at nucleotide as well as amino acid level in the selected blast-responsive WRKY genes. We also looked for variations in 1 kb promoter region of these genes. For identification of contigs of Tetep and HP2216 containing WRKY genes, blast analysis was carried out in Bioedit (29) offline software using sequences of selected WRKY genes from RGAP (http://rice.plantbiology.msu.edu/). Obtained contigs were used for identification of WRKY genes using Fgenesh software (http://www.softberry.com/). After confirmation of the selected WRKY genes for their identity, gene sequences along with 1 kb promoter regions were extracted from the contigs. The nucleotide and amino acid sequences of selected WRKY genes from Nipponbare, Tetep and HP2216 were used for multiple sequence alignment using ClustalW (http://www.ebi.ac.uk/Tools/msa/clustalw2/). Phylogenetic relationships among these sequences were visualized using Dendroscope 3 (30). A total of 1 kb promoter analysis was carried out using a motif-based sequence analysis tool, the MEME suite (http://meme-suite.org/) for identification of novel elements and PlantPAN 2.0 (31) (http://plantpan2.itps.ncku.edu.tw/) for identification of known promoter elements.

### Plant material and pathogen used

To validate *Magnaporthe*-responsive WRKY genes identified, we used two contrasting rice lines, a resistant genotype RIL4 [a recombinant inbred (RI) line derived from Tetep × HP2216 cross] and a susceptible genotype HP2216. One month old healthier plants raised in the rice nursery were transferred to pots and kept in the blast phenotyping facility under controlled environmental conditions (28–32°C temperature and relative humidity of 60%) with watering at frequent intervals. These plants were infected at booting stage in the neck of panicle with *M. oryzae* spores using syringe injection method. Two *M. oryzae* strains were used for infection: a virulent strain Mo-ni-025 and an avirulent strain Mo-ni-075*.* Panicle tissues were collected after 6, 12 and 24 h post-infection (hpi) along with mock samples injected with water.

### RNA isolation and expression analysis using quantitative real-time polymerase chain reaction

Samples were frozen in liquid nitrogen and stored at −80°C. RNA was isolated from each sample in two biological replicates using Spectrum™ Plant Total RNA Kit (Sigma-Aldrich, USA) as per manufacturer’s instructions. RNA was reverse-transcribed with random primers using High-Capacity cDNA Reverse Transcription kit (Applied Biosystems, USA). For quantitative real-time polymerase chain reaction (qRT-PCR), *Actin* gene was used as normalizer. Primer sequences of *WRKY* and *Actin* genes are given in Supplementary data Table S3. Each biological replicate had three technical replicates for transcript abundance studies. qRT-PCR was done in the total volume of 10 μl with 150 ng cDNA, 5 μl SYBR green (Brilliant III Ultra-Fast SYBR® Green QPCR Master Mix by Agilent Technologies, Santa Clara, CA, USA), 0.4 μl of ROX dye and 10 μM of each primer. Amplification was carried out in 96-well plates using LightCycler 480II machine by Roche (Basel, Switzerland) and the cycling conditions used were 95°C for 30 s, 58°C for 15 s and 72°C for 20 s, for 40 cycles. The melting point determination or dissociation curves were studied and analyzed at the end of PCR cycles.

## Results

### Statistics of RiceMetaSysB database

**Figure 1:**
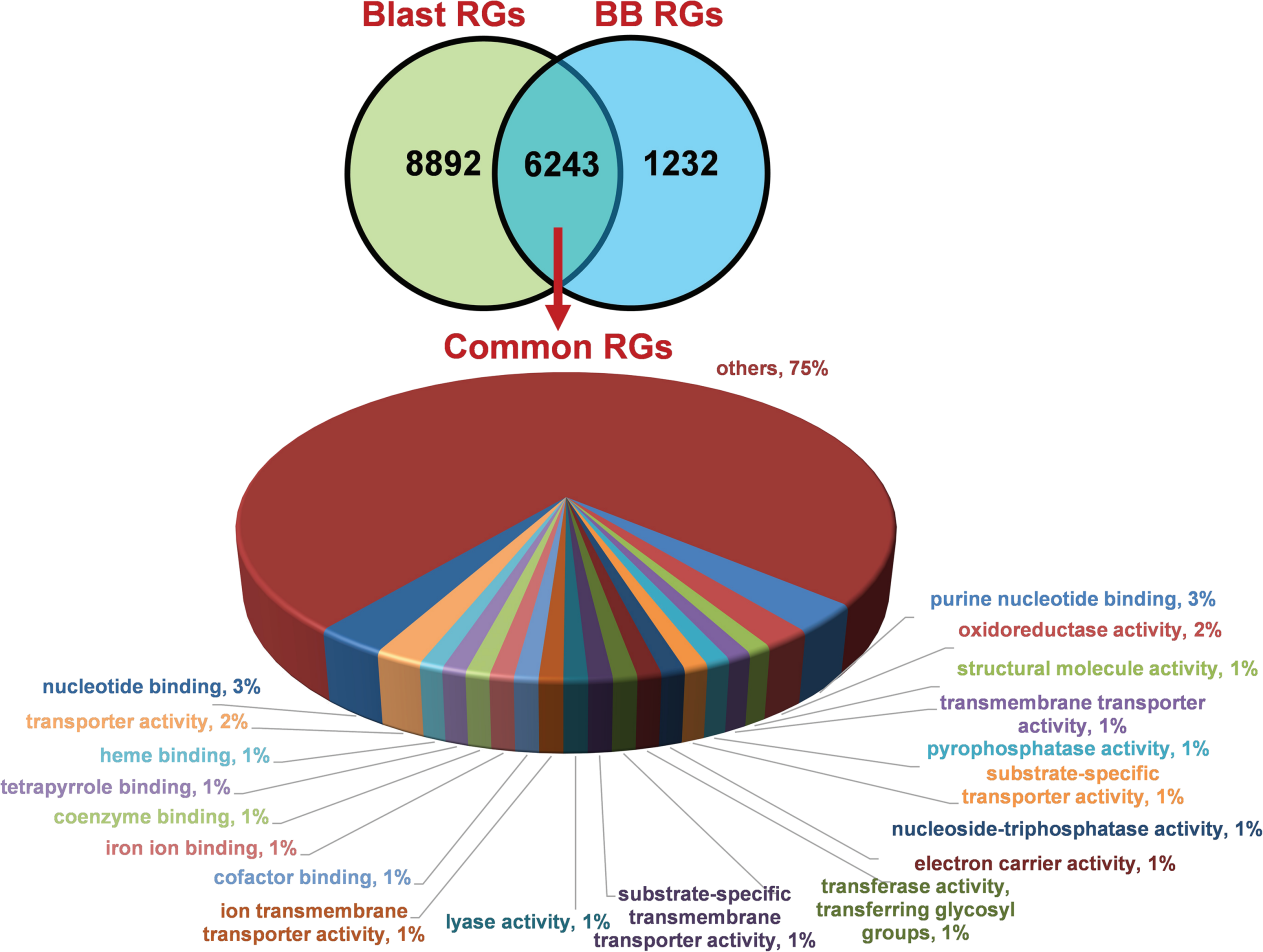
Distribution and functional annotation of common 6243 DEGs under rice blast and BB RGs.

**Figure 2:**
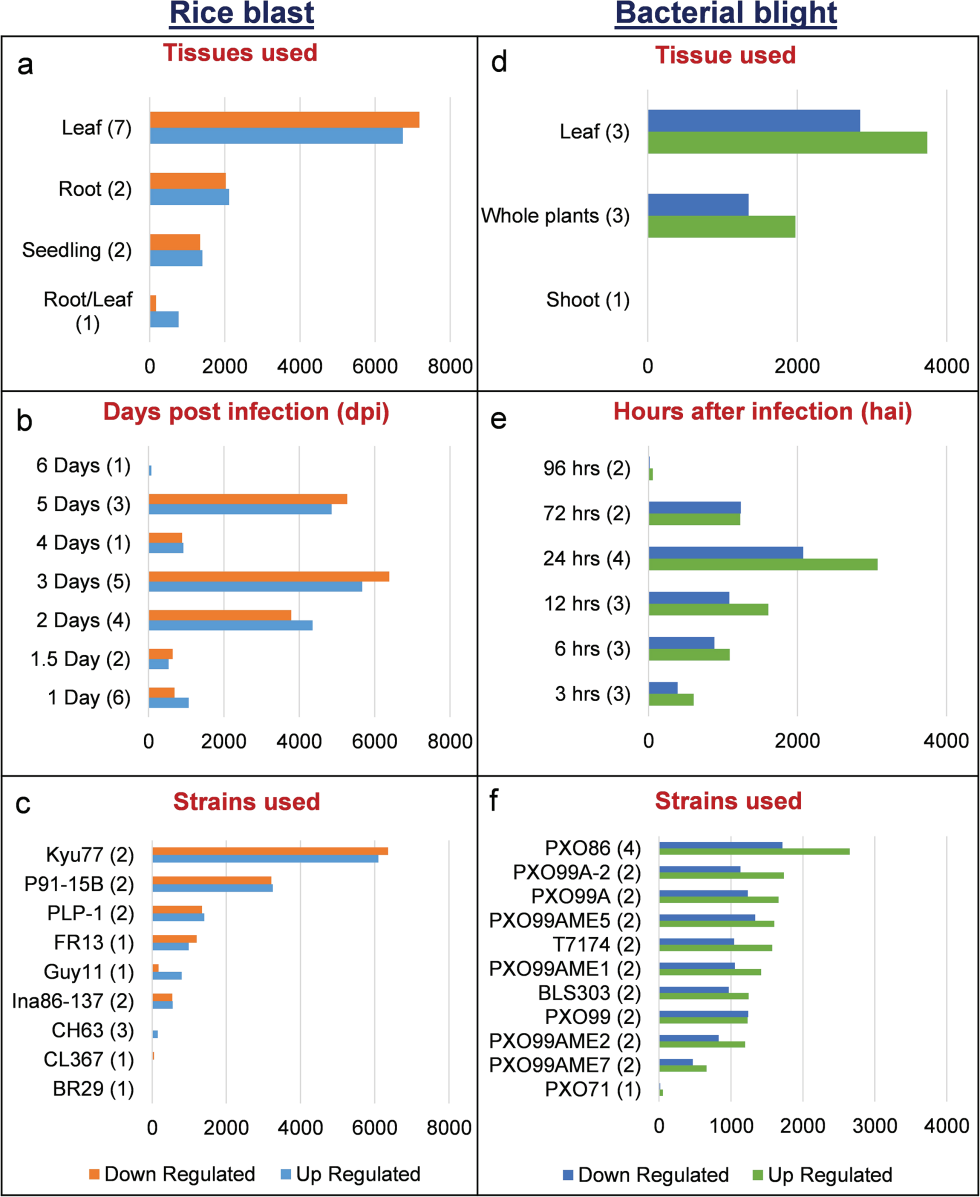
Distribution of DEGs in RiceMetaSys (biotic stress). **(A–C)** Distribution of rice blast RGs across tissues,dpi and strains. **(D–F)** Distribution of bacterial leaf blight RGs across tissues, hpi and strains.

**Figure 3:**
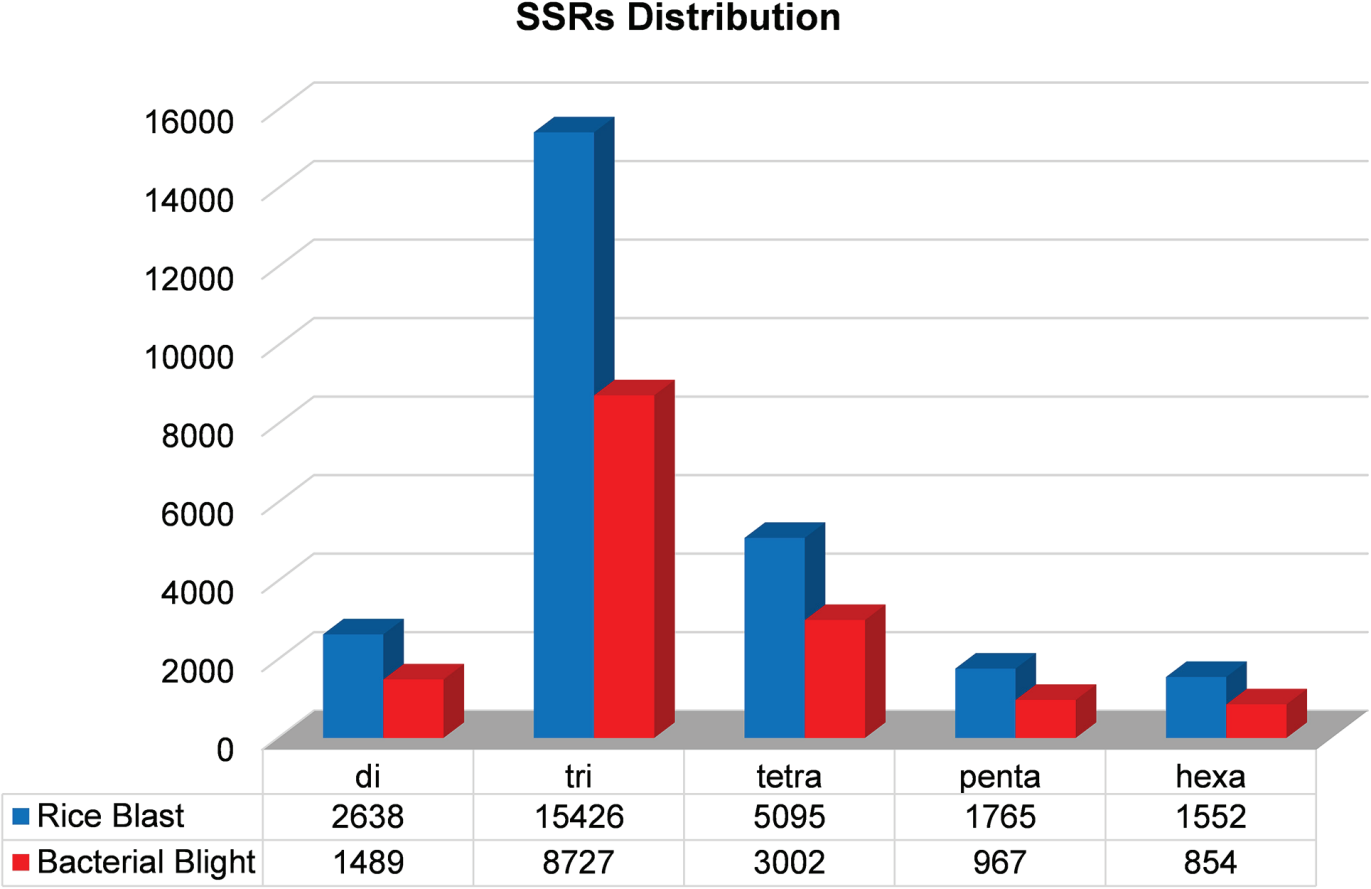
Distribution of microsatellites in the rice blast and BB RGs.

We identified 15 135 DEGs for rice blast disease from 10 GEO microarray data sets and 7475 DEGs for BB disease from 5 GEO microarray data sets. Of these, 6243 genes were found to be common to both rice blast and BB suggesting that molecular response to rice blast is more complicated than that of BB (Figure 1). Gene ontology analysis revealed that 41%, 41% and 18% of the rice blast DEGs were involved in biological process (BP), cellular component (CC) and molecular function (MF), respectively. Similarly, in case of BB, 39%, 44% and 17% of the DEGs were present under BP, CC and MF classes. In case of common disease RGs, a large number of them encoded undefined expressed proteins, followed by adenosine tri phosphate (ATP) binding, nucleotide binding and transporter activity (Figure 1). Under rice blast infection, the number of downregulated genes was more than that of upregulated genes. Contrastingly under BB infection, higher number of DEGs was found to be upregulated. Most often used tissue, strain and time points in blast and BB studies are presented in Figure 2. To facilitate breeders to follow the inheritance of these genes in their breeding populations, we identified SSR motifs in the DEGs of both rice blast and BB RGs and designed primers for the same. SSRs found in rice blast DEGs amounted to 9365 while in BB DEGs it was 5375. Further, SSRs were classified based on the motif length like di, tri, tetra, penta and hexa (Figure 3). Trinucleotide repeats were the most frequent SSR motifs in both rice blast and BB RGs.

### Important features of database

**Figure 4:**
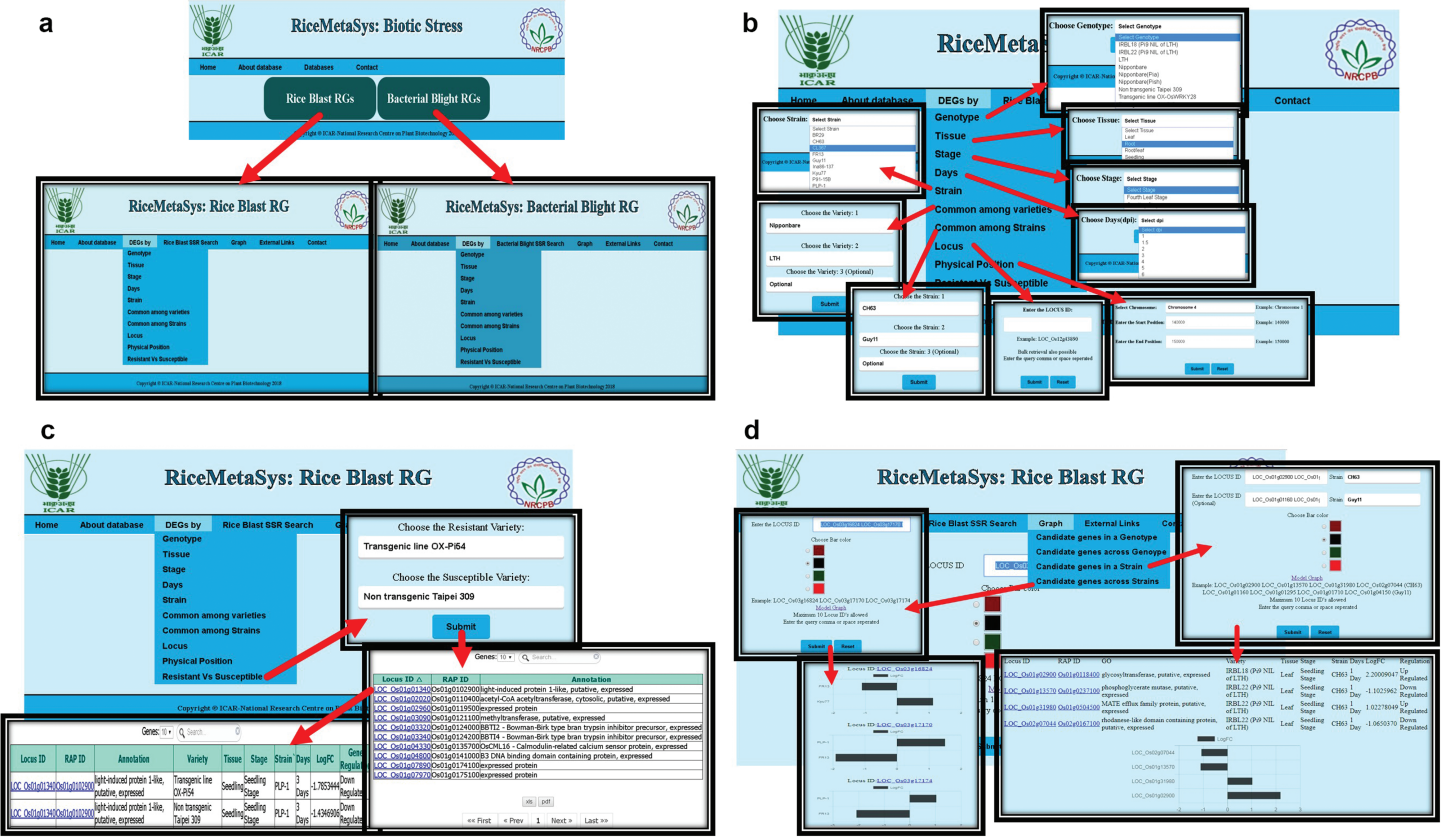
**(A)** Hompage of the RiceMetaSysB (biotic stress) database and two separate links for rice blast and BB RGs. **(B)** Different search options available in the database like genotype, tissue, stage, days, strain, common among varieties/strains, locus, physical position and resistant vs. susceptible genotypes search. **(C)** Resistant vs. susceptible search input, its output and hyperlink of locus search. **(D)** Interactive graph results of candidate genes in single and multiple strains.

Freely accessible and user-friendly web interfaces can help researcher to identify most robust candidate genes. Separate tabs/links have been designed to represent rice blast and BB DEGs under the RiceMetaSysB database (Figure 4). The common genes to rice blast and BB can be viewed separately from the homepage. Besides the identity, tissue and stage of the genotype used for study, in case of biotic stresses, strain used for infection and time points [days post-inoculation (dpi)/hours after inoculation (hai)] chosen for sampling for gene expression are also important. Hence, search options specific to each one of these parameters have been incorporated in the database so that users can retrieve candidate genes based on their need and objectives. All query outputs are in tabular format that can be retrieved in either ‘pdf’ or ‘spreadsheet’ format. There is a provision to sort the results based on FC, LOC ID (gene locus ID) and nature of regulation. Further, to make the results biologically more meaningful, candidate genes between disease-resistant and susceptible genotypes have been enabled. The user can also retrieve DEGs common to two or more strains or varieties. Physical position search option can facilitate researchers to retrieve DEGs in a particular chromosomal location of interest in the genome with the help of chromosome number and start and end positions. This search facility would be a valuable one for those following forward genetic approaches. Locus search facility provides the user to identify gene expression with the input of LOC IDs. Promoters of the selected DEGs can also be retrieved from the ‘DEG promoter’ tab. SSR search is enabled in this database through LOC IDs as input. The result window provides information on the SSR motif and forward and reverse primers. SSR query LOC IDs are hyperlinked to ISM-ILP database. Both locus search and SSR search options allow the users to retrieve batch queries. Further, external links have been provided for ISM-ILP database and pathway analysis and enrichement tools. The user can generate a graphical representation of up to 10 DEGs from single or multiple genotypes and strains. This option is very useful to visualize the expression level among genotypes and strains.

### Selection of DE WRKY genes from rice blast RGs

To demonstrate the utility of RiceMetaSysB database, we chose WRKY genes. To retrieve the DE WRKY genes from the database, locus IDs of all WRKY genes present in rice were submitted in ‘Locus ID’ search tab under the DEG panel (Figure 4). From 102 MSU IDs of WRKY genes submitted, a total of 316 entries were retrieved. These entries were in the tabular format with columns representing MSU and RAP IDs of the gene along with its annotation, growth stage, tissue and variety used in the experiment, pathogen strain and duration after which samples were collected and log FC and direction of regulation (up or down) of the gene. Sorting as per expression levels (logFC >5) recorded 47 entries. After filtering the list and removing the duplicate entries, 18 WRKY genes from the publically available leaf blast experiments finally remained (Table 1). From the panicle blast transcriptome experiment carried out in our laboratory in two rice varieties Tetep (resistant) and HP2216 (susceptible), we had earlier identified 12 WRKY genes with higher and upregulated expression in the blast-resistant line (data not shown). Between these two sets of WRKY genes, 8 were common to both leaf blast and panicle blast experiments while 10 were unique to leaf blast experiments and 4 to panicle blast experiments. Since we decided to check their expression in neck or panicle blast, eight WRKY genes common to both data sets and four genes unique to the panicle blast experiment were selected for expression analysis (Supplementary data Figure S2).

**Table 1:** WRKY genes identified from RiceMetaSysB database under blast infection with upregulation of 5 or above Log FC

**Locus ID**	**Annotation**	**Variety**	**Strain**	**Days**	**LogFC**
LOC_Os01g09100	WRKY10	Nipponbare	Kyu77	5	5.6
		Nipponbare (Pish)	Kyu77	5	5.4
LOC_Os01g14440	WRKY1	Nipponbare	FR13	4	5.1
LOC_Os01g40260	WRKY77	Nipponbare (Pish)	Kyu77	3	6.1
		Nipponbare (Pish)	Kyu77	2	6.0
		Nipponbare (Pia)	P91-15B	2	5.6
		Nipponbare	P91-15B	3	5.3
		Nipponbare	Kyu77	5	5.2
LOC_Os01g43650	WRKY11	Nipponbare	Kyu77	5	5.8
LOC_Os01g51690	WRKY26	Nipponbare (Pish)	Kyu77	2	6.6
		Nipponbare (Pish)	Kyu77	3	6.1
		Nipponbare	Kyu77	3	5.7
LOC_Os01g53040	WRKY14	Nipponbare	Kyu77	5	5.0
LOC_Os01g61080	WRKY24	Nipponbare	Kyu77	5	6.9
		Nipponbare	Guy11	2	5.4
LOC_Os02g08440	WRKY71	Nipponbare	FR13	4	5.0
LOC_Os02g26430	WRKY42	Nipponbare	Kyu77	5	7.0
		Nipponbare (Pish)	Kyu77	3	6.3
LOC_Os02g53100	WRKY32	Nipponbare (Pish)	Kyu77	5	6.5
		Nipponbare	Kyu77	5	5.5
LOC_Os03g21710	WRKY79	Nipponbare (Pish)	Kyu77	2	5.3
LOC_Os05g39720	WRKY70	Nipponbare	Kyu77	5	8.2
		Nipponbare (Pish)	Kyu77	3	6.4
		Nipponbare	Kyu77	3	6.4
LOC_Os05g49620	WRKY19	Nipponbare	Kyu77	5	7.5
		Nipponbare (Pish)	Kyu77	2	7.4
		Nipponbare (Pish)	Kyu77	3	7.2
		Nipponbare	Kyu77	3	6.8
		Nipponbare (Pish)	Kyu77	5	6.3
		Nipponbare	P91-15B	1	5.6
		Nipponbare (Pia)	P91-15B	2	5.5
LOC_Os06g44010	WRKY28	Nipponbare (Pia)	Ina86-137	2	9.8
		Nipponbare	Kyu77	5	5.9
		Nipponbare	Kyu77	3	5.3
LOC_Os09g25070	WRKY62	Nipponbare	Kyu77	5	6.6
		Nipponbare	Kyu77	3	5.4
LOC_Os11g02530	WRKY40	Nipponbare (Pish)	Kyu77	2	6.8
		Nipponbare (Pish)	Kyu77	3	6.4
		Nipponbare (Pish)	Kyu77	5	6.3
		Nipponbare (Pia)	P91-15B	2	6.0
		Nipponbare	Kyu77	3	5.4
LOC_Os11g29870	WRKY72	Nipponbare (Pish)	Kyu77	2	5.1
LOC_Os12g02450	WRKY64	Nipponbare (Pish)	Kyu77	2	6.6
		Nipponbare (Pia)	P91-15B	2	6.2
		Nipponbare (Pish)	Kyu77	3	6.2
		Nipponbare (Pish)	Kyu77	5	6.2
		Nipponbare	Kyu77	3	5.1

### Variations in the candidate WRKY genes at nucleotide level

**Figure 5:**
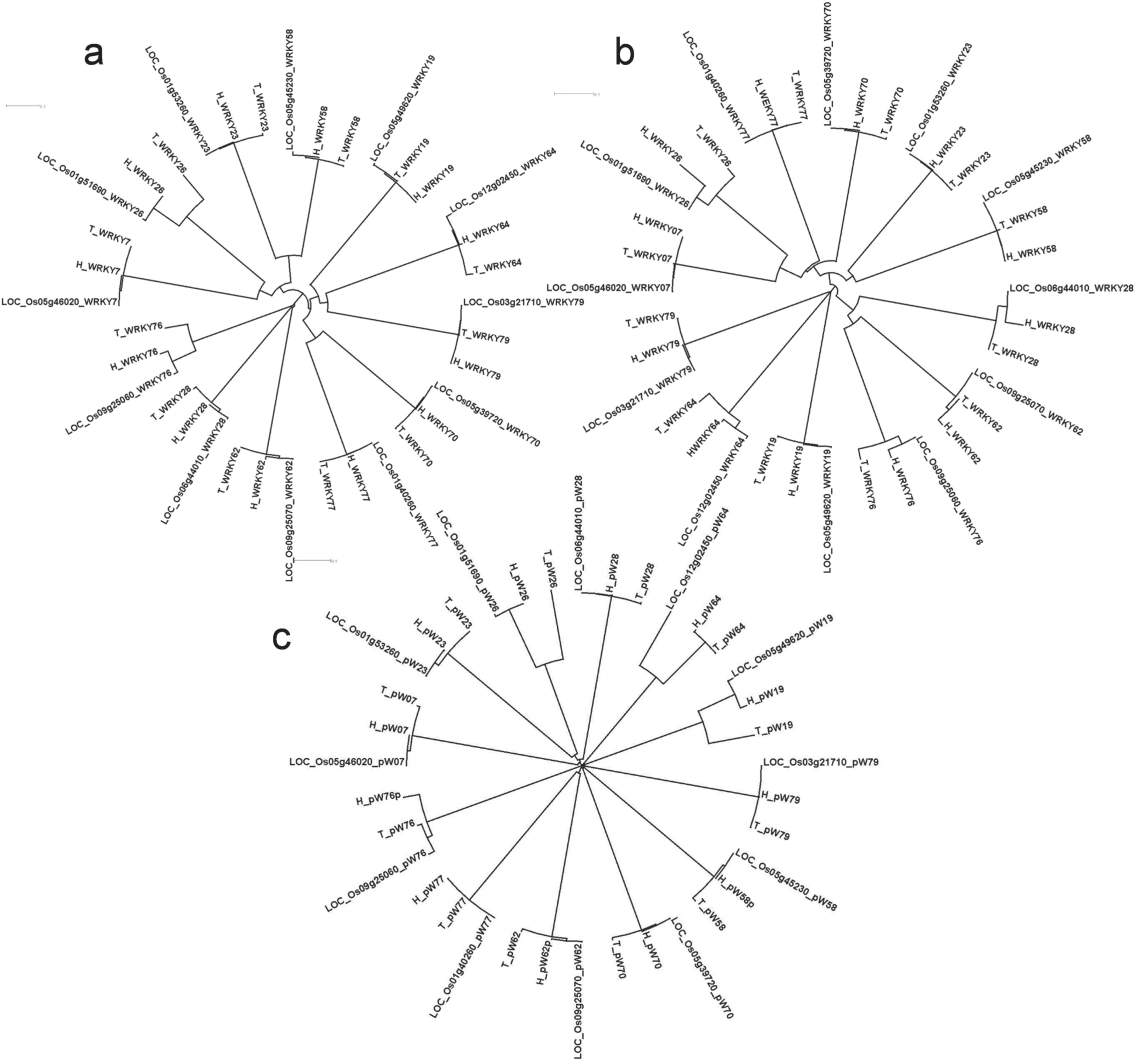
Phylogenetic analysis of 12 DE WRKY genes in blast experiments using **(A)** complete genes, **(B)** proteins and **(C)** 1 kb promoter regions in three genotypes Tetep, HP2216 and Nipponbare using ClustalX2.1 tool.

Significant structural differences were observed in the WRKY genes between Tetep and HP2216. The information about single nucleotide polymorphisms (SNPs) and Insertions/Deletions (InDels) present among the two genotypes along with the reference, Nipponbare, is given in Supplementary data Table S4**.***WRKY64* had the most number of variations, with 40 base substitutions and 5 InDels followed by *WRKY23* which had 15 base substitutions and 8 InDels. Among these eight InDels, seven were deletions while only one was an insertion in the susceptible genotype HP2216. *WRKY77* was the least variable with just one deletion (CAC) in HP2216 followed by *WRKY76* and *WRKY28* both of which had two base substitutions and one deletion in HP2216. Overall, across the 12 WRKY genes, there were 27 InDels with 19 insertions and 8 deletions in the rice blast-resistant genotype Tetep. We also generated phylogenetic tree where three WRKY genes namely, *WRKY26, WRKY28* and *WRKY76* were evolutionary different in Tetep and HP2216 (Figure 5A). Of these three genes, *WRKY26* and *WRKY28* had five InDels each, with five and two substitutions, respectively.

### Variations in WRKY genes at protein level

All the 12 selected WRKY genes have conserved WRKY and zinc finger domains in them in both the genotypes, except WRKY58 which had no zinc finger domain. Two WRKY domains were present in WRKY70 (Type I) whereas single WRKY domain was present in rest of the 11 genes. The sequence of WRKY domain, WRKYGQK, was conserved in all proteins except in WRKY07, WRKY26 and WRKY77, all of which had WRKYG(K)K. The CCHH zinc finger domain was present in all WRKYs except WRKY19, WRKY64 and WRKY79 where CCHC zinc finger domain (Type III) was present. Standards prescribed by Wu *et al.* (32) and Ross *et al.* (33) have been followed in categorizing the *WRKY* genes. The variations in amino acid sequences among the 12 WRKY proteins are summarized in Supplementary data Table S5**.** WRKY76 had two larger (92 and 22 amino acid long) and one smaller deletion (6 amino acid long) in HP2216 besides 8 amino acid substitutions (Supplementary data Figure S3). WRKY26 had 22 variations which included 2 larger InDels, 4 smaller InDels and 16 amino acid substitutions. WRKY64 also had 22 variations of which 21 were single amino acid substitution, and the remaining one was an amino acid deletion in Tetep. WRKY77 had only one amino acid deletion in Tetep while WRKY23 had only one substitution. Both WRKY28 and WRKY79 had two amino acid substitutions. WRKY7, WRKY19 and WRKY58 each had one substitution and one InDel. Phylogenetic analysis showed that three WRKY genes namely WRKY79, WRKY64 and WRKY19 are evolutionary different from the rest of the WRKY genes at amino acid level. Further, within-gene variation was observed in WRKY26, WRKY64, WRKY76 and WRKY28, which had multiple variations in their amino acid sequences (Figure 5B).

### Variations in 1 kb promoter regions of WRKY gene

Considerable variations in the form of SNPs and InDel were observed in the promoter regions. Four variations were observed in the promoter region of *WRKY76*. Three variations were observed in the promoter of *WRKY64* whereas two variations were present in the promoter region of *WRKY19*. *WRKY26* and *WRKY58* both had one variation each in the promoter region. These structural variations are shown in Supplementary data Figure S4. Phylogenetic analysis showed that promoter sequences of *WRKY19*, *WRKY26* and *WRKY64* were completely different after 505, 406 and 554 bp upstream from ATG in Tetep and HP2216, respectively (Figure 5C). These promoter sequences were further subjected to search for unknown motifs and six such motifs were identified. We observed that the number and positions of motifs were conserved in all three genotypes in all 12 promoters except the absence of motif MEME1 in the promoter of WRKY76 in HP2216 and absence of motif MEME3 in the promoter of *WRKY64* in Nipponbare **(**Supplementary data Figure S5**)**. We also checked the known AP2, bHLH, bZIP, WRKY, MYB, HSF, Dehydrin, ERF and NAC transcription factors’ binding domains in the promoter sequences using PlantPAN software. The details of different motifs present in the promoters are shown in Supplementary data Table S6. Interestingly, the susceptible genotype HP2216 had the promoter elements identical to that of Nipponbare, which is also susceptible to rice blast, while the resistant genotype was very different in its *cis*-element composition with the absence of 17 elements (5 dehydrin, 5 bHLH, 4 bZIP, 2 WRKY and 1 HSF) and presence of two new elements (one each of AP2 and HSF).

### Expression analysis of WRKY genes under panicle blast

**Figure 6:**
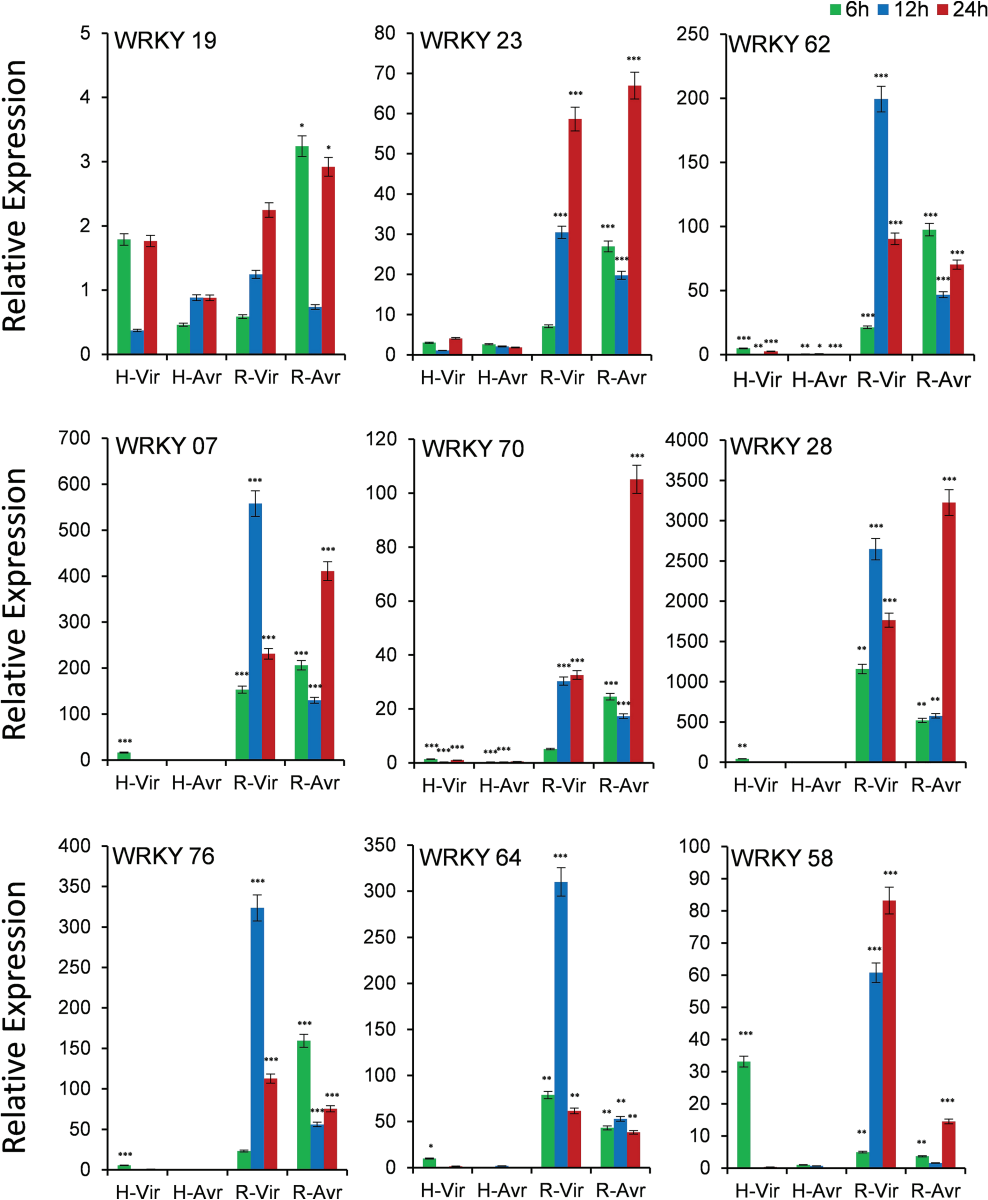
Expression analysis of WRKY genes in blast-infected panicle tissues at three-time intervals. T means Tetep; H, HP2216 with virulent and avirulent *M. oryzae* strains. Asterisks indicate a significant difference compared with untreated at *P* < 0.05, by Student’s *t*-test.

Out of 12 genes, we could get quantifiable amplification in 9 genes (Figure 6). As the blast disease-resistant genotype Tetep did not flower in our experiment, we used its derivative RIL4 that showed resistance reaction akin to its parent Tetep. Though the expression of *WRKY19* gene was observed in both the genotypes, transcript abundance was more in case of resistant genotype RIL4, which increased with increasing time course, particularly against infection with the virulent pathogen. The gene *WRKY23* also had a very high level of expression in resistant RIL4 compared to susceptible HP2216 again in which increase in expression level with increase in time period in response to infection by the virulent pathogen was observed. Similarly, *WRKY62*, *WRKY07*, *WRKY76* and *WRKY64* had a very high level of expression in RIL4 against the virulent pathogen strain at 12 hpi. The gene *WRKY70* also had a very high level of expression in RIL4, but the expression was the highest against the avirulent pathogen after 24 hpi. The gene *WRKY58* had a moderate expression in susceptible genotype HP2216 against the virulent pathogen strain, which decreased sharply after 6 hpi; however, in the resistant genotype it had a very high level of expression against the virulent strain. In case of the gene *WRKY28*, the highest expression was observed in RIL4 against both the avirulent as well as virulent strains.

## Discussion

With the advent of microarray and RNA-seq technologies, a huge amount of genome-wide expression data is available in rice, especially under biotic stress conditions. Besides the use of resistant genotypes or a contrasting pair of genotypes (in terms of their disease response) for studying gene expression under disease infection, a large number of transgenic lines and NILs transformed/introgressed with major R genes have been used for characterization of response to disease infection in rice (10, 18, 20, 34). Most of the publicly available databases like OryzaExpress, RicePLEX, ROAD, RiceSRTFDB, QlicRice, OryGenesDB, RiceXPro and qTeller and commercial platforms like GeneMapper and Genevestigator developed either cater to abiotic stresses or gene/QTL (quantitative trait loci) mapping projects and the features of the same have been discussed in detail in Sandhu *et al.* (24). Recently, the gene expression modules and regulatory networks under various abiotic stresses in rice have been elucidated through a comprehensive database called EGRIN (35). As none of these databases were either breeder-friendly or amenable for any direct utilization in crop improvement, we initially developed a breeder-friendly database, named RiceMetaSys, for DEGs under drought and salt stresses in rice (24). Now we extended our work on abiotic stresses to the two most important biotic stresses of rice, blast and BB. For this, all the available microarray sets related to blast and BB in rice were collated and normalized, so that these experiments can be brought on a common platform (36). As our biotic stress-based database (unlike the abiotic one) included the Agilent arrays too, a huge number of samples were analyzed amounting to 241 samples for rice blast and 186 for BB giving rise to 15135 and 7475 disease RGs, respectively. Following our earlier database, we named the current one as RiceMetaSysB for biotic stresses. In addition to the search options provided in the earlier database for retrieval of candidate genes specific to genotype, tissue and developmental stage of the host, the current one carries options based on strain and hours/dpi as well as ‘common genes among varieties’ and ‘strains’ to identify most robust candidate genes. Similarly, the 2D representation of the data in the form of graph provides expression profiles not only across genes and genotypes but also across strains. Further, this database provides candidate gene-specific SSR and intron-specific length polymorphism for marker-assisted breeding of the robust DEGs.

In the earlier version of the database, we could not demonstrate the practical utility (24). Hence, here we identified blast-responsive WRKY genes using RiceMetaSysB database. There are more than 100 WRKY transcription factors present in both japonica and indica rice, out of which many WRKYs are shown to be DE during blast disease development in rice (18, 37–38). Several WRKY genes like *OsWRKY13*, *OsWRKY31*, *OsWRKY45*, *OsWRKY53*, *OsWRKY71*, *OsWRKY89, OsWRKY76* and *OsWRKY67* have been functionally characterized for resistance to blast or BB by generating transgenics (18, 39–46). From a wide array of experiments, using our DB, we could identify 18 blast-responsive WRKY genes.

Using an RI population from the blast-resistant Tetep and susceptible HP2216, *Pi54* gene has been already identified and introgressed in other cultivated lines for blast resistance (47). For our validation studies, we used these two genotypes and an RI line derived from these two genotypes. We observed the expression of *WRKY19* in both the genotypes with both virulent and avirulent strains demonstrating its utility as a biomarker to ascertain *Magnaporthe* attack on rice crop. *WRKY23*, *WRKY28* and *WRKY70* were *Magnaporthe*-responsive but did not contribute to blast resistance as they were upregulated in the resistant line but with higher expression in response to the avirulent strain than the virulent strain. On the other hand, *WRKY7*, *WRKY58*, *WRKY62*, *WRKY64* and *WRKY76* were found to be the promising candidate genes providing resistance to panicle blast as they are highly expressed only in the resistant line, that too in response to the virulent strain. When we checked these genes in the literature, we found that *WRKY28*, *WRKY62* and *WRKY76* are negative regulators of rice blast (17, 45, 48). This was surprising for us as we found its higher expression in resistant genotypes, that too in response to virulent strain. Then we checked its structural variation and we found that proteins of *WRKY26*, *WRKY28*, *WRKY62*, *WRKY64* and *WRKY76* are very different in Tetep and HP2216 with large number of amino acid substitutions and InDels. This shows that probably alleles from Tetep are contributing for resistance whereas its alternative allele may be contributing for susceptibility. We have scope to conclude this because in earlier study by Tao *et al.* (44) regarding *WRKY45* from rice showed that a pair of alleles of *WRKY45* plays opposite roles in rice–bacteria interaction. Such a conclusion is also supported by observation that the *cis*-element composition of the resistant genotype, Tetep, was completely different from that of the susceptible genotypes, HP2216 and Nipponbare. As many as five bHLH and five dehydrin elements were lost in Tetep indicating that they do have a role in inducing susceptibility or losing resistance. The role of dehydrin, an important transcription factor in abiotic stress tolerance, has recently been implicated in biotic stress tolerance (49). However, we can conclusively comment on the contribution of these genes or *cis*-elements only after their complementation test by developing transgenics. However, from this analysis we are able to show that RiceMetaSysB database can play a very important role in providing robust candidate genes responsible for rice blast and BB stresses.

## Conclusion and future prospects

RiceMetaSysB is an open source and user-friendly web interface for identification of most robust genes for rice blast and BB diseases. Physical position and SSR search options are expected to be more meaningful for breeders while other options will be useful for pathologists and molecular biologists working on these two diseases. The utility of the database has been demonstrated in our study which identified *WRKY7*, *WRKY58*, *WRKY62*, *WRKY64* and *WRKY76* to be potential candidate WRKY genes for contributing toward panicle blast resistance in rice. More candidate genes belonging to other gene families can also be identified this way and used in functional genomics and crop improvement.

## Supplementary Material

Supplementary Data

## Notes

**Database URL:** http://14.139.229.201/ricemetasysb/
