# Supplementary material for: RiceMetaSysB: a database of blast and bacterial blight responsive genes in rice and its utilization in identifying key blast-resistant WRKY genes
Source: Database (Oxford). 2019 Feb 11;2019:baz015. doi: 10.1093/database/baz015 (PMC6369264; doi:10.1093/database/baz015)
Supplement: Supplementary Data [file suppl_blastblight_baz015_supp.pdf]

Table S1: Datasets used for Rice Blast analysis.

**GEO ID**	**Genotype**	**Tissue**	**Stage**	**Strain**
GSE7256	Nipponbare (S)	Leaf	Seedling Stage	FR13
GSE18361	Nipponbare (S)	Root/leaf	Seedling Stage	Guy11
GSE28308	Non-transgenic Taipei 309 (S)	Seedling	Seedling Stage	PLP-1
	Transgenic line OX-Pi54 (R)			
GSE30941	Nipponbare (S)	Leaf	Seedling Stage	FR13
				CL367
				BR29
GSE39635	Nipponbare [Pia] (R)	Leaf	Fourth Leaf Stage	Ina86-137
	Transgenic line OX-OsWRKY28 (S)			
GSE41798	IRBL18 (Pi9 NIL of LTH) (R)	Leaf	Seedling Stage	CH63
	IRBL22 (Pi9 NIL of LTH) (R)			
	LTH (S)			
GSE62893	Nipponbare (Pia) (R)	Leaf	Fourth Leaf Stage	P91-15B
	Nipponbare (S)			
GSE62894	Nipponbare (Pish) (R)	Leaf	Fourth Leaf Stage	Kyu77
	Nipponbare (S)			
GSE62895	Nipponbare (Pia) (R)	Root	Fourth Leaf Stage	P91-15B
	Nipponbare (S)			
GSE62422	Nipponbare (Pia) (R)	Leaf	Fourth Leaf Stage	P91-15B
	Nipponbare (S)			

Table S2: Datasets used for Bacterial blight analysis.

GEO ID	Genotype	Tissue	Stage	Strain
GSE36272	Nipponbare (S)	Leaf	14 Days	PXO99A
				PXO99AME7
				PXO99AME5
				T7174
				PXO86
				BLS303
				PXO99AME1
				PXO99AME2
				PXO99A-2
				MOCK
	IR24 (S)	Leaf	14 Days	PXO99A
				PXO99AME7
				PXO99AME5
				T7174
				PXO86
				BLS303
				PXO99AME1
				PXO99AME2
				PXO99A-2
				MOCK
GSE33411	IR24 (S)	Whole plants	2 Weeks	PXO86
	IRBB5 (R)			
	IRBB7 (R)			
GSE16793	Nipponbare (S)	Shoot	2 Weeks	PXO99A
GSE43050	IR24 (R)	Leaf	35 Days	PXO71
GSE63337	Osaba1-1 (R)	Leaf	6.5 Leaf Stage	PXO99
	Nipponbare (S)			

Table S3: List of primers used for quantitative real time PCR.

**Locus id**	**WRKY name**	**Primer sequence**
LOC_Os01g40260	OsWRKY77_qF	GAGGAAGTACGGCAAGAAGAT
	OsWRKY77_qR	CTCCACCCGCTTCTTCAC
LOC_Os01g51690	OsWRKY26_qF	GAGGTCGGACGATGAGATACT
	OsWRKY26_qR	CCGGTAGTAGTTCCTCGGATTA
LOC_Os01g53260	OsWRKY23_qF	GCAACGTGAAGAAGCAAGTG
	OsWRKY23_qR	AACTGGGAGAGGAACTGGA
LOC_Os03g21710	OsWRKY79_qF	GTTCATGCAGTGGGACGA
	OsWRKY79_qR	GTTTCCGCCGTCGTTCT
LOC_Os05g39720	OsWRKY70_qF	TACTCTTACACGAGCCAGCA
	OsWRKY70_qR	GGCTGCTCAAAGAACGACAT
LOC_Os05g45230	OsWRKY58_qF	CAGAAGGCCGTCAAGAACA
	OsWRKY58_qR	CAGGTTCTCTGTTGCAGTTTG
LOC_Os05g49620	OsWRKY19_qF	AGGTAGTGGCAGAGCCTAT
	OsWRKY19_qR	AGAAGTCGCTGCTGAACTG
LOC_Os06g44010	OsWRKY28_qF	TACGAGGGCGAGCACAA
	OsWRKY28_qR	CTGGTGCTGTGAGTGCTTG
LOC_Os09g25070	OsWRKY62_qF	GAGGAGGTGGTGCAAGTG
	OsWRKY62_qR	GTGGGATTTGATCCACAACTCTA
LOC_Os12g02450	OsWRKY64_qF	TCGTCGAAGCATCACAGATTAG
	OsWRKY64_qR	AACAGGTGACGGAGATGTTG
LOC_Os05g46020	OsWRKY07_qF	TCCGGACGAAGTCAGAGAT
	OsWRKY07_qR	CGGTAGTAGTTCCTTGGGTTG
LOC_Os09g25060	OsWRKY76_qF	GTGCTCCATCTCCATCAACT
	OsWRKY76_qR	CGGCACACCTCCTTGAG
LOC_Os03g50885	OsActin_qF	GATCTGGCATCACACCTTCTAC
	OsActin_qR	CTGGGTCATCTTCTCACGATTG

Table S4: Structural variations at nucleotide level in Tetep and HP2216 in comparison with Nipponbare.

Gene name	Position	Nipponbare	HP2216	Tetep
**WRKY7**	116	T	T	C
	500	G	G	A
	507	G	G	A
	511-522	CCGTGTTCAATG	deletion	CCGTGTTCAATG
	584	C	T	T
	900	T	T	C
	935	T	T	C
	1006-1192	*	*	ATATACTATGGCTGTGTTCGTTCC AGTGACATTGGGTGAGAAAGGTC CTCGTTTTCCGCGCGCACGCTTCC CAAACTACTAAACGGTGCGTTTTT TGNNAAATAGTTAATACTCAATTA ATCATATGCTAATGGCCCACCTCG TTTTGCGTATCTTCCCAATCTCCCT ACTCCCCTTCTCCTCAAACACACC CTAT
	1516	T	T	C
	1895	C	C	T
**WRKY19**	88	C	C	G
	558	A	A	T
	559	A	A	C
	623	C	C	T
	625-632	CCGCCGCCG	CCGCCGCCG	deletion
	799	G	G	A
**WRKY23**	514	C	C	A
	524	G	G	A
	731	G	G	A
	947	T	T	C
	1190	A	A	*
	1246	G	G	A
	1326	*	*	A
	1578	G	G	T
	1945-1947			AAA INSERTION
	2161-2162			GG INSERTION
	2442	T	T	C
	2655	G	G	A
	2762	*	*	C
	2791	*	*	A
	2859-2864	ACTTGA	ACTTGA	deletion
	2999	T	T	C
	3022	A	A	*
	3024	A	A	T
	3074			TA INSERTION
	3186-3189			TATA INSERTION
**WRKY26**	78	A	A	G

	114-116	*	*	ACG INSERTION
	188	G	G	T
	189-230	GCTCCGGCAGCGGCTACGG CGCCGCCTCGTACGTTGCTC CA	GCTCCGGCAGCGGCTACGGCG CCGCCTCGTACGTTGCTCCA	DELETION
	241	G	G	C
	314-316			CCA INSERTION
	363	C	C	G
	410-412			GGC INSERTION
	455	T	T	A
	499	C	C	G
	516	T	T	*
	523-525	ATA	ATA	DELETION
**WRKY28**	95	C	C	T
	205-206	TC	TC	AA
	1043-1056	ACAACCCGTGCCCG	DELETION	ACAACCCGTGCCCG
**WRKY58**	257	A	A	C
	277	C	C	A
	825	C	C	T
	837-839	TCT	TCT	DELETION
**WRKY62**	902	T	T	A
	1117	C	C	A
	1124	A	A	*
	1158	T	T	C
	1175	A	A	G
	1331	A	A	G
	1359-1368	*	*	GTTGGTTTTT
	1385	G	G	A
	1917	G	G	A
**WRKY64**	99	G	C	C
	111	A	G	G
	142	A	G	G
	154	C	T	T
	163	T	C	C
	168	T	C	C
	169	C	T	T
	187-190	TAGC	*	*
	222	A	G	G
	231	T	G	G
	246-247	TC	AT	AT
	256-258	*	CAT	CAT
	281	C	T	T
	289	T	T	A
	354	G	G	A
	419	G	G	T
	430	C	C	T
	463	C	C	G

	469	A	A	G
	473	A	A	G
	479	G	G	C
	484	A	A	G
	492	A	A	T
	503-507	GATTC	GATTC	CCACA
	509-512	CCGC	CCGC	GATG
	521	G	G	C
	524-526	GGC	GGC	CAT
	530	C	C	G
	559-560	TG	TG	CA
	570-572	CTT	CTT	*
	590	A	A	T
	700	G	G	T
	749	G	G	A
	753	T	T	*
	766	A	A	C
	785-787	CAA	CAA	*
	794	G	G	A
	803	T	T	C
	878	C	C	A
	902	A	A	G
	905	*	*	T
	924	T	T	C
	1005-1010	*	ATATAT	*
	1043	T	C	C
	1079	T	A	T
	1112	C	C	A
	1115	T	C	T
	1139	T	C	T
	1143	G	A	G
	1157	G	T	G
	1175	C	T	C
	1195	C	C	T
	1201	C	T	C
	1488	G	G	T
	1498	C	T	C
**WRKY70**	57	T	T	G
	107	T	T	C
	483	T	T	G
	509	T	T	C
	534	A	A	G
	764	A	A	T
	1250	C	C	A
	1825	G	G	T

	1860	T	T	C
	2243	C	C	T
	2484	G	G	A
	2789	T	T	*
**WRKY76**	569	G	G	A
	895-944	CGAGCGCGGACAGCTCGCTGCTGGTGGCGACGTACGAGGGCGAGCAC	deletion	CGAGCGCGGACAGCTCGCTGCTGGTGGCGACGTACGAGGGCGAGCAC
	901	C	C	T
**WRKY77**	824-826	CAC	deletion	CAC
**WRKY79**	86	T	T	C
	492	A	A	T
	898	*	*	T
	1363	A	A	C
	1365	C	C	T

Table S5: Variations at amino acid level in HP2216 and Tetep in comparison with Nipponbare

Gene name	Position	Nipponbare	HP2216	Tetep
**WRKY7**	80	A	A	T
	81-84	AVFN	*	AVFN
**WRKY19**	85-97	QVMPPGHGGLISG		QVMPPGHGGLISG
	184	A	A	T
**WRKY23**	155	T	T	K
**WRKY26**	15	N	S	N
	27	*	D	*
	52-65	GSGSGYGAASYVAP	*	GSGSGYGAASYVAP
	66	T	S	T
	94	*	P	*
	110	A	G	A
	126	*	G	*
	147	T	M	T
	184	N	N	*
	186	Y	Y	F
	187	R	R	G
	188	C	C	T
	190-209	TEGCNVKKRVERDK NDPRYVV	TEGCNVKKRVERDKN DPRYVV	*
	210	T	T	F
	211	M	T	V
	214	G	G	L
	215	I	I	V
	216	H	H	F
	217	N	N	K
	218	H	H	F
	219	V	V	G
	221	P	P	F
**WRKY28**	13	P	P	S
	50	P	P	T
**WRKY58**	72	A	A	E
	153	L	L	*
**WRKY62**	29-31	*	*	CVQ
**WRKY64**	9	P	S	S
	33	R	R	K
	55	G	G	C
	73	I	I	V
	75	E	E	Q
	79	Q	Q	L
	83	D	D	P
	84	S	S	H
	85	P	P	D

	86	H	H	D
	89	D	D	H
	90	G	G	H
	92	L	L	V
	102	V	V	I
	106	S	S	*
	112	S	S	C
	160	N	N	K
	192	H	H	Q
	220	A	A	V
	222	S	L	S
	318	A	A	S
	321	A	V	A
**WRKY70**	90	I	I	M
	99	I	I	T
	178	T	T	N
	370	A	A	S
	477	P	P	L
**WRKY76**	73	L	*	M
	74	T	*	D
	75	E	*	A
	76	V	*	A
	77	I	*	W
	78	A	*	R
	79	R	*	G
	80	L	*	G
	81	Y	*	V
	82	G	*	G
	83	G	*	C
	84	Q	*	S
	85	I	*	P
	86	P	*	V
	87	R	*	C
	88	L	*	L
	89	G	*	D
	90	L	*	L
	91	D	*	C
	92	G	*	V
	93	S	*	G
	94	A	A	L
	97	P	P	V
	99	P	P	E
	100-134	*	*	PSAARHELLDRPAGCR GGGDSKSMTNDEAKIL EAK

	136	S	S	T
	137	P	P	Q
	138	L	L	M
	140	G	G	E
	141	K	K	E
	142-147	*	*	NRRLTE
	220-311	TRDNPSPRAYFRCAFAPSCPVKKKVQRSAEDSSLLVATYEGEHNHPHPSPRAGELPAAAGGAGGSLPCSISINSSGPTITLDLTKNGGAVQV	*	TRDNPSPRAYFRCAFAPSCPVKKKVQRSAEDSSLLVATYEGEHNHPHPSPRAGELPAAAGGAGGSLPCSISINSSGPTITLDLTKNGGAVQV
**WRKY77**	93	H	H	*
**WRKY79**	29	V	V	A
	216	N	N	H

Table S6: Selected known Transcription factor (TF) binding elements in the promoters of DEG WRKYs.

Sl. No.	Pro ID	Nipponbare		HP2216		Tetep	
		Binding sequence	TF family	Binding sequence	TF family	Binding sequence	TF family
	pW07	acGTCGGtgt	AP2	acGTCGGtgt	AP2	acGTCGGtgt	AP2
		acgccCACGTcggtgtc	bHLH	acgccCACGTcggtgtc	bHLH		
		cCCACGtcg	bZIP	cCCACGtcg	bZIP	cCCACGtcg	bZIP
		cTTGACta	WRKY	cTTGACta	WRKY	tGTCAA	WRKY
		ccAACCAact	MYB	ccAACCAact	MYB	ccAACCAact	MYB
		aaaatTTTCT	HSF	aaaatTTTCT	HSF		
		CTCGG	Dehydrin	CTCGG	Dehydrin	CTCGG	Dehydrin
		agCGGCT	ERF	agCGGCT	ERF	agCGGCT	ERF
	pW19	aACGTGc	bHLH	aACGTGc	bHLH		
		aaaACGTGct	bZIP	aaaACGTGct	bZIP	aaaACGTGct	bZIP
		cgGTCAAagt	WRKY	cgGTCAAagt	WRKY	cgGTCAAagt	WRKY
		tggTAGGTtg	MYB	tggTAGGTtg	MYB	tggTAGGTtg	MYB
		caaCCTTAgt	AP2	caaCCTTAgt	AP2	caaCCTTAgt	AP2
		AGAAAgtttt	HSF	AGAAAgtttt	HSF	AGAAAgtttt	HSF
		CCGAG	Dehydrin	CCGAG	Dehydrin		
	pW23	tcCGGCCgcc	AP2	tcCGGCCgcc	AP2	tcCGGCCgcc	AP2
		gtggcgCACGTccggccg	bHLH	gtggcgCACGTccggccg	bHLH		
		taaCGTGGc	bZIP	taaCGTGGc	bZIP		
		ttTTGACttc	WRKY	ttTTGACttc	WRKY	ttTTGACttc	WRKY
		aaACCTAtct	MYB	aaACCTAtct	MYB	gggTGGTTgt	MYB
		TCGAC	Dehydrin	TCGAC	Dehydrin	AGAACttttt	HSF
	pW26	ccaCCGACgt	AP2	ccaCCGACgt	AP2	ccaCCGACgt	AP2
		cGAAGCttca	HSF	cGAAGCttca	HSF	cGAAGCttca	HSF
		gCACGTg	bHLH	gCACGTg	bHLH	gCACGTg	bHLH
		acatgCACGTgta	bZIP	acatgCACGTgta	bZIP	acatgCACGTgta	bZIP
		actTTGACca	WRKY	actTTGACca	WRKY	CCAAC	Dehydrin
		GTAGG	Dehydrin	GTGGG	Dehydrin	TGACC	WRKY
		ggCGGCT	ERF	ggCGGCT	ERF	AGCCGca	ERF
	pW28	GCATGtgg	bHLH	GCATGtgg	bHLH	GCATGtgg	bHLH
		tacCAGCTgg	bZIP	tacCAGCTgg	bZIP	tacCAGCTgg	bZIP
		gaGTCAAcaa	WRKY	gaGTCAAcaa	WRKY	gaGTCAAcaa	WRKY
		gcAACCAgcc	MYB	gcAACCAgcc	MYB	gcAACCAgcc	MYB
		AGAACttttt	HSF	AGAACttttt	HSF	AGAACttttt	HSF
		GTCGT	Dehydrin	GTCGT	Dehydrin	GTCGT	Dehydrin
	pW58	ccgACGTCac	bZIP	ccgACGTCac	bZIP	ccgACGTCac	bZIP
		gcaCCGACac	AP2	gcaCCGACac	AP2	gcaCCGACac	AP2
		ctgcccCACGTgttcctg	bHLH	ctgcccCACGTgttcctg	bHLH	ctgcccCACGTgttcctg	bHLH
		ccgTTGACga	WRKY	ccgTTGACga	WRKY	ccgTTGACga	WRKY
		gcAACCActa	MYB	gcAACCActa	MYB	gcAACCAcca	MYB
		CCGAG	Dehydrin	CCGAG	Dehydrin	CCGAG	Dehydrin

Sl. No.	Pro ID	Nipponbare		HP2216		Tetep	
		Binding sequence	TF family	Binding sequence	TF family	Binding sequence	TF family
	pW62	ctcggtCACGTaagcacg	bHLH	ctcggtCACGTaagcacg	bHLH	ctcggtCACGTaagcacg	bHLH
		ttCACGTttt	bZIP	ttCACGTttt	bZIP	ttCACGTttt	bZIP
		cgGTCAAg	WRKY	cgGTCAAg	WRKY	cgGTCAAg	WRKY
		gaAACCAacg	MYB	gaAACCAacg	MYB	gaAACCAacg	MYB
		ccTACGAggc	AP2	ccTACGAggc	AP2	ccTACGAggc	AP2
		CCAAC	Dehydrin	CCAAC	Dehydrin	CCAAC	Dehydrin
		AGCCGcg	ERF	AGCCGcg	ERF	AGCCGcg	ERF
		ACGGCgcc	NAC	ACGGCgcc	NAC	ACGGCgcc	NAC
	pW64	GAACGtac	bHLH	GAACGtac	bHLH		
		atgCGTCAca	bZIP	atgCGTCAca	bZIP		
		tagTGGTTgc	MYB	tagTGGTTgc	MYB	tagTGGTTgc	MYB
		tgaagGTTCT	HSF	tgaagGTTCT	HSF	tgaagGTTCT	HSF
		ATCGG	Dehydrin	CCTAC	Dehydrin		
	pW70	tccACGTCac	bZIP	tccACGTCac	bZIP	tccACGTCac	bZIP
		cCACGTc	bHLH	cCACGTc	bHLH	cCACGTc	bHLH
		gctTTGACca	WRKY	gctTTGACca	WRKY	gctTTGACca	WRKY
		ccAACCAtct	MYB	ccAACCAtct	MYB	ccAACCAtct	MYB
		agtatATTCT	HSF	agtatATTCT	HSF	aaaCCTTAat	AP2
		CTGAC	Dehydrin	CTGAC	Dehydrin	agtatATTCT	HSF
						CTGAC	Dehydrin
	pW76	gTGACGtaag	bZIP	gTGACGtaag	bZIP		
		attcgtgACGTGcgctcc	bHLH	attcgtgACGTGcgctcc	bHLH	attcgtgACGTGcgctcc	bHLH
		cTTGACcg	WRKY	cTTGACcg	WRKY	cTTGACcg	WRKY
		tcACCTAgcc	MYB	tcACCTAgcc	MYB	tcACCTAgcc	MYB
		AGAATattat	HSF	AGAATattat	HSF	AGAACatgct	HSF
		CACGCgtgccgaac	AP2	CACGCgtgccgaac	AP2	CACGCgtgccgaac	AP2
		CCGAA	Dehydrin	CCGAA	Dehydrin	CCGAA	Dehydrin
	pW77	gtGACGTact	bZIP	gtGACGTact	bZIP		
		GCGCGcgc	bHLH	GCGCGcgc	bHLH	GCGCGcgc	bHLH
		tCCGTTgagt	MYB	tCCGTTgagt	MYB	tCCGTTgagt	MYB
		CCGTC	Dehydrin	CCGTC	Dehydrin		
		TGACT	WRKY	TGACT	WRKY		
	pW79	gtgtgaCACGTcgtcccg	bHLH	gtgtgaCACGTcgtcccg	bHLH		
		ctaCGTGGa	bZIP	ctaCGTGGa	bZIP	ctaCGTGGa	bZIP
		cgGTCAAagg	WRKY	cgGTCAAagg	WRKY		
		gCCGTTcggt	MYB	gCCGTTcggt	MYB	gCCGTTcggt	MYB
		cgaaaTTTCT	HSF	cgaaaTTTCT	HSF	cgaaaTTTCT	HSF
		GTCTG	Dehydrin	GTCTG	Dehydrin		

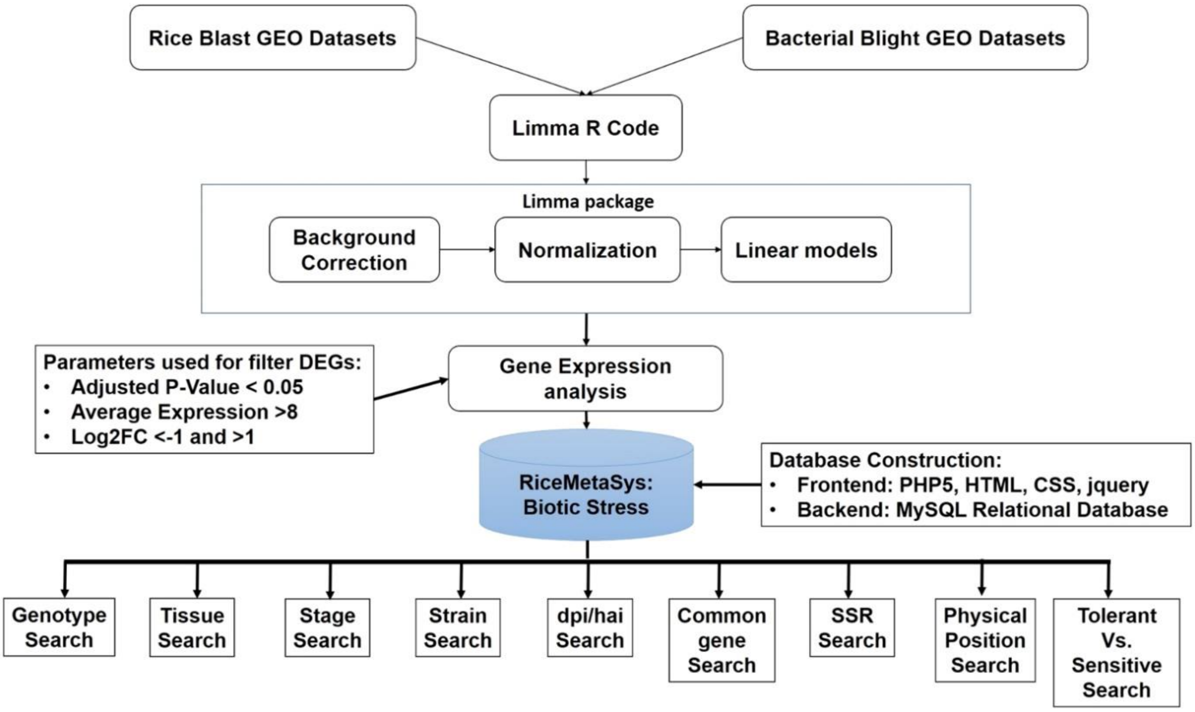

Fig. S1: Metadata Analysis and Database Design: Schematic Diagram of RiceMetaSys (Biotic Stress) Database. Expression datasets were downloaded from the NCBI Gene expression Omnibus (GEO) Database. The datasets were analyzed by using limma package in R platform. Different kinds of search options were enabled for users to retrieve responsive genes from the Database.

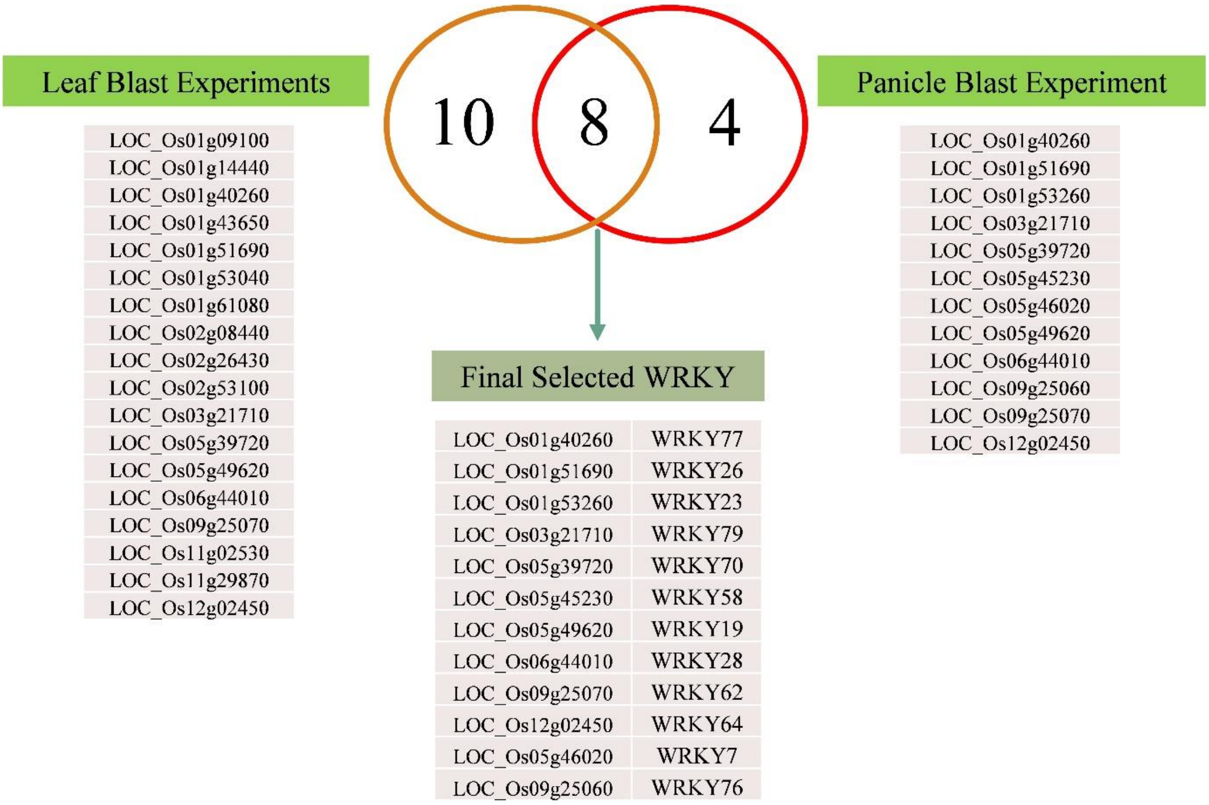

Fig. S2: Differentially expressed WRKY genes selected from RiceMetaSysB database and from panicle blast transcriptome.

```
                                  10        20        30        40        50        60        70
                         ....|....|....|....|....|....|....|....|....|....|....|....|....|....|
LOC_Os12g02450_WRKY64    ---------------------------------------------------------------------- 1
HWRKY64                  ---------------------------------------------------------------------- 1
T_WRKY64                 ---------------------------------------------------------------------- 1
LOC_Os03g21710_WRKY79    ---------------------------------------------------------------------- 1
H_WRKY79                 ---------------------------------------------------------------------- 1
T_WRKY79                 ---------------------------------------------------------------------- 1
LOC_Os05g49620_WRKY19    ---------------------------------------------------------------------- 1
H_WRKY19                 ---------------------------------------------------------------------- 1
T_WRKY19                 ---------------------------------------------------------------------- 1
LOC_Os05g46020_WRKY07    ---------------------------------------------------------------------- 1
T_WRKY07                 ---------------------------------------------------------------------- 1
H_WRKY07                 ---------------------------------------------------------------------- 1
LOC_Os01g51690_WRKY26    ---------------------------------------------------------------------- 1
H_WRKY26                 ---------------------------------------------------------------------- 1
T_WRKY26                 ---------------------------------------------------------------------- 1
H_WEKY77                 ---------------------------------------------------------------------- 1
T_WRKY77                 ---------------------------------------------------------------------- 1
LOC_Os01g40260_WRKY77    ---------------------------------------------------------------------- 1
LOC_Os05g39720_WRKY70    MTAAPGSLPLVNSRPVSLSLAASRSSFSSLLSGGAGSSLNLMTPPSSLPPSSPSSYFGGVSSSGFLDSPI 70
H_WRKY70                 MTAAPGSLPLVNSRPVSLSLAASRSSFSSLLSGGAGSSLNLMTPPSSLPPSSPSSYFGGVSSSGFLDSPI 70
T_WRKY70                 MTAAPGSLPLVNSRPVSLSLAASRSSFSSLLSGGAGSSLNLMTPPSSLPPSSPSSYFGGVSSSGFLDSPI 70
LOC_Os01g53260_WRKY23    ---------------------------------------------------------------------- 1
H_WRKY23                 ---------------------------------------------------------------------- 1
T_WRKY23                 ---------------------------------------------------------------------- 1
T_WRKY58                 ---------------------------------------------------------------------- 1
H_WRKY58                 ---------------------------------------------------------------------- 1
LOC_Os05g45230_WRKY58    ---------------------------------------------------------------------- 1
LOC_Os06g44010_WRKY28    ---------------------------------------------------------------------- 1
H_WRKY28                 ---------------------------------------------------------------------- 1
T_WRKY28                 ---------------------------------------------------------------------- 1
T_WRKY62                 ---------------------------------------------------------------------- 1
H_WRKY62                 ---------------------------------------------------------------------- 1
LOC_Os09g25070_WRKY62    ---------------------------------------------------------------------- 1
LOC_Os09g25060_WRKY76    ---------------------------------------------------------------------- 1
H_WRKY76                 ---------------------------------------------------------------------- 1
T_WRKY76                 ---------------------------------------------------------------------- 1

                                  80        90       100       110       120       130       140
                         ....|....|....|....|....|....|....|....|....|....|....|....|....|....|
LOC_Os12g02450_WRKY64    ---------------------------------------------------------------------- 1
HWRKY64                  ---------------------------------------------------------------------- 1
T_WRKY64                 ---------------------------------------------------------------------- 1
LOC_Os03g21710_WRKY79    ---------------------------------------------------------------------- 1
H_WRKY79                 ---------------------------------------------------------------------- 1
T_WRKY79                 ---------------------------------------------------------------------- 1
LOC_Os05g49620_WRKY19    ---------------------------------------------------------------------- 1
H_WRKY19                 ---------------------------------------------------------------------- 1
T_WRKY19                 ---------------------------------------------------------------------- 1
LOC_Os05g46020_WRKY07    ---------------------------------------------------------------------- 1
T_WRKY07                 ---------------------------------------------------------------------- 1
H_WRKY07                 ---------------------------------------------------------------------- 1
LOC_Os01g51690_WRKY26    ---------------------------------------------------------------------- 1
H_WRKY26                 ---------------------------------------------------------------------- 1
T_WRKY26                 ---------------------------------------------------------------------- 1
H_WEKY77                 ---------------------------------------------------------------------- 1
T_WRKY77                 ---------------------------------------------------------------------- 1
LOC_Os01g40260_WRKY77    ---------------------------------------------------------------------- 1
LOC_Os05g39720_WRKY70    LLTPSLFPSPTTTGALFSWITTATATAAIAPESQVQGGVKDEQQQYSDFTFLPTASTAPATTMAGATATT 140
H_WRKY70                 LLTPSLFPSPTTTGALFSWITTATATAAIAPESQVQGGVKDEQQQYSDFTFLPTASTAPATTMAGATATT 140
T_WRKY70                 LLTPSLFPSPTTTGALFSWMTTATATAATAPESQVQGGVKDEQQQYSDFTFLPTASTAPATTMAGATATT 140
LOC_Os01g53260_WRKY23    ---------------------------------------------------------------------- 1
H_WRKY23                 ---------------------------------------------------------------------- 1
T_WRKY23                 ---------------------------------------------------------------------- 1
T_WRKY58                 ---------------------------------------------------------------------- 1
H_WRKY58                 ---------------------------------------------------------------------- 1
LOC_Os05g45230_WRKY58    ---------------------------------------------------------------------- 1
LOC_Os06g44010_WRKY28    ---------------------------------MAKMLPPPSQSVPSRPPSWLYIPPRRRHGTFTSSCAF 37
H_WRKY28                 ---------------------------------MAKMLPPPSQSVPSRPPSWLYIPPRRRHGTFTSSCAF 37
T_WRKY28                 ---------------------------------MAKMLPPPSQSVSSRPPSWLYIPPRRRHGTFTSSCAF 37
T_WRKY62                 ---------------------------------------------------------------------- 1
H_WRKY62                 ---------------------------------------------------------------------- 1
LOC_Os09g25070_WRKY62    ---------------------------------------------------------------------- 1
LOC_Os09g25060_WRKY76    ---------------------------------------------------------------------- 1
H_WRKY76                 ---------------------------------------------------------------------- 1
T_WRKY76                 ---------------------------------------------------------------------- 1
```

```
                               150       160       170       180       190       200       210
                      ....|....|....|....|....|....|....|....|....|....|....|....|....|....|
LOC_Os12g02450_WRKY64 ---------------------------------------------------MKNSSNKRPLVADQWHPSS 19
HWRKY64               ---------------------------------------------------MKNSSNKRSLVADQWHPSS 19
T_WRKY64              ---------------------------------------------------MKNSSNKRSLVADQWHPSS 19
LOC_Os03g21710_WRKY79 ----------------------------------------------------------MAFSSEGGVPAE 12
H_WRKY79              ----------------------------------------------------------MAFSSEGGVPAE 12
T_WRKY79              ----------------------------------------------------------MAFSSEGGVPAE 12
LOC_Os05g49620_WRKY19 ---------------------------------------------------------MVELCGGEGEGQI 13
H_WRKY19              ---------------------------------------------------------MVELCGGEGEGQI 13
T_WRKY19              ---------------------------------------------------------MVELCGGEGEGQI 13
LOC_Os05g46020_WRKY07 -----------------------------------------------------------MAAVGAHAAV- 10
T_WRKY07              -----------------------------------------------------------MAAVGAHAAV- 10
H_WRKY07              -----------------------------------------------------------MAAVGAHAAV- 10
LOC_Os01g51690_WRKY26 ---------------------------------------------------------MYMAAAAAGASTP 13
H_WRKY26              ---------------------------------------------------------MYMAAAAAGASTP 13
T_WRKY26              ---------------------------------------------------------MYMAAAAAGASTP 13
H_WEKY77              -------------------------------------------------------------MSSLYPSLL 9
T_WRKY77              -------------------------------------------------------------MSSLYPSLL 9
LOC_Os01g40260_WRKY77 -------------------------------------------------------------MSSLYPSLL 9
LOC_Os05g39720_WRKY70 SNSFMQDSMLMAPLGGDPYNGEQQQPWSYQEPTMDADTRPAEFTSSAAAGDVAGNGSYSQVAAPAAAGGF 210
H_WRKY70              SNSFMQDSMLMAPLGGDPYNGEQQQPWSYQEPTMDADTRPAEFTSSAAAGDVAGNGSYSQVAAPAAAGGF 210
T_WRKY70              SNSFMQDSMLMAPLGGDPYNGEQQQPWSYQEPTMDADNRPAEFTSSAAAGDVAGNGSYSQVAAPAAAGGF 210
LOC_Os01g53260_WRKY23 ----------------------------------------MENLQLQGDD--HDDEALPHFPYFAVPSPP 28
H_WRKY23              ----------------------------------------MENLQLQGDD--HDDEALPHFPYFAVPSPP 28
T_WRKY23              ----------------------------------------MENLQLQGDD--HDDEALPHFPYFAVPSPP 28
T_WRKY58              ----------------------------------------MDGLEAAAGDQQHGRLLIPQLPAAYLASSS 30
H_WRKY58              ----------------------------------------MDGLEAAAGDQQHGRLLIPQLPAAYLASSS 30
LOC_Os05g45230_WRKY58 ----------------------------------------MDGLEAAAGDQQHGRLLIPQLPAAYLASSS 30
LOC_Os06g44010_WRKY28 RLSPSSPSSPPPPVLDFQYIQFMDSWIEQTSLSLDLNVGLPSTARRSSAPAAPIKVLVEENFLSFKKDHE 107
H_WRKY28              RLSPSSPSSPPPPVLDFQYIQFMDSWIEQTSLSLDLNVGLPSTARRSSAPAAPIKVLVEENFLSFKKDHE 107
T_WRKY28              RLSPSSPSSPPPTVLDFQYIQFMDSWIEQTSLSLDLNVGLPSTARRSSAPAAPIKVLVEENFLSFKKDHE 107
T_WRKY62              ---------------------------------------MDDDGDGSSSPTDDSAAAGLLPLFSRSPCVQ 31
H_WRKY62              ---------------------------------------------------------------------- 1
LOC_Os09g25070_WRKY62 ---------------------------------------MDDDGDGSSSPTDDSAAAGLLPLFSRSP--- 28
LOC_Os09g25060_WRKY76 -------------------------------------MDAAWRGGVGCSPVCLDLCVGLSPVREPSAARH 33
H_WRKY76              ---------------------------------------------------------------------- 1
T_WRKY76              -------------------------------------MDAAWRGGVGCSPVCLDLCVGLSPVREPSAARH 33

                               220       230       240       250       260       270       280
                      ....|....|....|....|....|....|....|....|....|....|....|....|....|....|
LOC_Os12g02450_WRKY64 VCCDHRAALREIARGQSLVTQLRAIVLPALHSDERGDLAAQ--------------MLEGILDCSRKAISE 75
HWRKY64               VCCDHRAALREIARGQSLVTQLRAIVLPALHSDERGDLAAQ--------------MLEGILDCSRKAISE 75
T_WRKY64              VCCDHRAALREIAKGQSLVTQLRAIVLPALHSDERCDLAAQ--------------MLEGILDCSRKAVSQ 75
LOC_Os03g21710_WRKY79 RVAAAVNDLVEVRDG---LVRLRGFLPPPPQAEQSSSRPPC--------------AAELMDATMSKLMSA 65
H_WRKY79              RVAAAVNDLVEVRDG---LVRLRGFLPPPPQAEQSSSRPPC--------------AAELMDATMSKLMSA 65
T_WRKY79              RVAAAVNDLVEVRDG---LARLRGFLPPPPQAEQSSSRPPC--------------AAELMDATMSKLMSA 65
LOC_Os05g49620_WRKY19 MLATELAQLRAMARE------LEAKMDPDRVAAR--ELCRA--------------LASSVDRSIRLAASC 61
H_WRKY19              MLATELAQLRAMARE------LEAKMDPDRVAAR--ELCRA--------------LASSVDRSIRLAASC 61
T_WRKY19              MLATELAQLRAMARE------LEAKMDPDRVAAR--ELCRA--------------LASSVDRSIRLAASC 61
LOC_Os05g46020_WRKY07 YHHPVSGLSAPAGDAAYS---MS---SYFSHGGSSTSSS-----------ASSFSAALAAATTPPLPDPS 63
T_WRKY07              YHHPVSGLSAPAGDAAYS---MS---SYFSHGGSSTSSS-----------ASSFSAALAAATTPPLPDPS 63
H_WRKY07              YHHPVSGLSAPAGDAAYS---MS---SYFSHGGSSTSSS-----------ASSFSAALAAATTPPLPDPS 63
LOC_Os01g51690_WRKY26 FNFCRHGSHAEYD-AVFSGSWMARRPSAAPHGGGASGSGSGSGYGAASYVAPTFGAAFRQQHLDLLDYLS 82
H_WRKY26              FNFCRHGSHAEYD-AVFSGSWMARRPSAAPHGGGASGSGSGSGYGAASYVAPTFGAAFRQQHLDLLDYLS 82
T_WRKY26              FSFCRHGSHAEYDDAVFSGSWMARRPSAAPHGGGASGS--------------SFGAAFRQQHLDLLDYLS 69
H_WEKY77              SLSESPAEYRQVGGGRYAGEDVVDDDDDMAAVADAVSSY------------LSFDMDDVEYYTPEVGFHS 67
T_WRKY77              SLSESPAEYRQVGGGRYAGEDVVDDDDDMAAVADAVSSY------------LSFDMDDVEYYTPEVGFHS 67
LOC_Os01g40260_WRKY77 SLSESPAEYRQVGGGRYAGEDVVDDDDDMAAVADAVSSY------------LSFDMDDVEYYTPEVGFHS 67
LOC_Os05g39720_WRKY70 RQQSRRSSDDGYNWRKYGQKQMKGSENPRSYYKCTFPGCPTKKKVEQSPDGQVTEIVYKGAHSHPKPPQN 280
H_WRKY70              RQQSRRSSDDGYNWRKYGQKQMKGSENPRSYYKCTFPGCPTKKKVEQSPDGQVTEIVYKGAHSHPKPPQN 280
T_WRKY70              RQQSRRSSDDGYNWRKYGQKQMKGSENPRSYYKCTFPGCPTKKKVEQSPDGQVTEIVYKGAHSHPKPPQN 280
LOC_Os01g53260_WRKY23 PLAVAPAASATTSDGHQHGPLEVLEQPPCSNNLHPDGLVDGPQLAAT---TAVPMMLPAMTSLDWQSLLQ 95
H_WRKY23              PLAVAPAASATTSDGHQHGPLEVLEQPPCSNNLHPDGLVDGPQLAAT---TAVPMMLPAMTSLDWQSLLQ 95
T_WRKY23              PLAVAPAASATTSDGHQHGPLEVLEQPPCSNNLHPDGLVDGPQLAAT---TAVPMMLPAMTSLDWQSLLQ 95
T_WRKY58              MAALSPAGDDWAAS------LILPDGGSAAAGVGEDDLGGG-----------------VMAAAAEESSCG 77
H_WRKY58              MAALSPAGDDWAAS------LILPDGGSAAAGVGEDDLGGG-----------------VMAAAAAESSCG 77
LOC_Os05g45230_WRKY58 MAALSPAGDDWAAS------LILPDGGSAAAGVGEDDLGGG-----------------VMAAAAAESSCG 77
LOC_Os06g44010_WRKY28 VEALEAELRRASEENKKLTEMLRAVVAKYTELQGQVNDMMSAAAAAAVNAGNHQSSTSEGGSVSPSRKRI 177
H_WRKY28              VEALEAELRRASEENKKLTEMLRAVVAKYTELQGQVNDMMSAAAAAAVNAGNHQSSTSEGGSVSPSRKRI 177
T_WRKY28              VEALEAELRRASEENKKLTEMLRAVVAKYTELQGQVNDMMSAAAAAAVNAGNHQSSTSEGGSVSPSRKRI 177
T_WRKY62              AEDLEEKLRRAMEENARLTRALDAILAGHHAHQR---------------------ALLAPSLSPPPPSAT 80
H_WRKY62              ---------------------------------------------------------------------- 1
LOC_Os09g25070_WRKY62 AEDLEEKLRRAMEENARLTRALDAILAGHHAHQR---------------------ALLAPSLSPPPPSAT 77
LOC_Os09g25060_WRKY76 ELLDRPAGCRGGGDSKSMTNDEAKIVEAKVTQMSEENRRLTEVIARLYGG---QIPRLGLDGSASPPRPV 100
H_WRKY76              ---------------------------------------------------------------ASPPRPV 7
T_WRKY76              ELLDRPAGCRGGGDSKSMTNDEAKILEAKVTQMS------------------------------EENRRL 73
```

```
                                290       300       310       320       330       340       350
                       ....|....|....|....|....|....|....|....|....|....|....|....|....|....|
LOC_Os12g02450_WRKY64  LQLQLSSDSPHDDDGHLDDKRRVRKIVSSSSDDDDHSSSKAAEDHNAKPLRQHKRRRFGD---------- 135
HWRKY64                LQLQLSSDSPHDDDGHLDDKRRVRKIVSSSSDDDDHSSSKAAEDHNAKPLRQHKRRRFGD---------- 135
T_WRKY64               LQLLLSSPHDDDDHHHVDDKRRVRKIISSS-DDDDHCSSKAAEDHNAKPLRQHKRRRFGD---------- 134
LOC_Os03g21710_WRKY79  MATLGGSG---DIAGEVDAAGRWTSVAESADPMVVRREGESSAGRTRRRRGGGSRSGRGR---------- 122
H_WRKY79               MATLGGSG---DIAGEVDAAGRWTSVAESADPMVVRREGESSAGRTRRRRGGGSRSGRGR---------- 122
T_WRKY79               MATLGGSG---DIAGEVDAAGRWTSVAESADPMVVRREGESSAGRTRRRRGGGSRSGRGR---------- 122
LOC_Os05g49620_WRKY19  FPPP-------------EHPPPAAGNAGRDAAFKKR-------------KGMAKVRRQVR---------- 95
H_WRKY19               FPPP-------------EHPPPAAGNAGRDAAFKKRQVMVPGHGGLISGKGMAKVRRQVR---------- 108
T_WRKY19               FPPP-------------EHPPPAAGNAGRDAAFKKRQVMVPGHGGLISGKGMAKVRRQVR---------- 108
LOC_Os05g46020_WRKY07  GSQFDISEFFFDDAPPAAVFNGAPTAA-----LPDGAAANAT---RSAAEAVPAPAPAAV---------- 115
T_WRKY07               GSQFDISEFFFDDAPPTAVFNGAPTAA-----LPDGAAANAT---RSAAEAVPAPAPAAV---------- 115
H_WRKY07               GSQFDISEFFFDDAPP----AGAPTAA-----LPDGAAANAT---RSAAEAVPAPAPAAV---------- 111
LOC_Os01g51690_WRKY26  DDQGVPAPPP-AAVPSASYVTPAPAMAPAEPVVPDAVAAAGG-YPRSVAAAAAAVAGEGR---------- 140
H_WRKY26               DDQGVPAPPP-AAVPSASYVTPAPAMAPAEPVVPDAVAAAGG-YPRSVAAAAAAVAGEGR---------- 140
T_WRKY26               DDQGVPAPPPPAAVPSASYVTPAPAMGPAEPVVPDAVAAAGGGYPRSVAAAAAAVAGEGR---------- 129
H_WEKY77               KQH---NPPPVAAAPLEAGGGREQSRR--------EAAVNLGKMDRGPAPVSGGAATGGV---------- 116
T_WRKY77               KQH---NPPPVAAAPLEAGGGREQSRR--------EAAVNLGKMDRGPAPVSGGAATGGV---------- 116
LOC_Os01g40260_WRKY77  KQH---NPPPVAAAPLEAGGGREQSRR--------EAAVNLGKMDRGPAPVSGGAATGGV---------- 116
LOC_Os05g39720_WRKY70  GRGRGGSGYALHGGAASDAYSSADALSGTPVATPENSSASFGDDEAVNGVSSSLRVASSVGGGEDLDDDE 350
H_WRKY70               GRGRGGSGYALHGGAASDAYSSADALSGTPVATPENSSASFGDDEAVNGVSSSLRVASSVGGGEDLDDDE 350
T_WRKY70               GRGRGGSGYALHGGAASDAYSSADALSGTPVATPENSSASFGDDEAVNGVSSSLRVASSVGGGEDLDDDE 350
LOC_Os01g53260_WRKY23  TCLQVPPPVLEQQQPAAAAQADQYSGENDHGDLQAAESSGAGNKEKQVMAKGGAGRPSGT---------- 155
H_WRKY23               TCLQVPPPVLEQQQPAAAAQADQYSGENDHGDLQAAESSGAGNKEKQVMAKGGAGRPSGT---------- 155
T_WRKY23               TCLQVPPPVLEQQQPAAAAQADQYSGENDHGDLQAAESSGAGNKEKQVMAKGGAGRPSGK---------- 155
T_WRKY58               GSSTVTSSGVTEAAAAAATTTR----------------RGRGNGKK----AGGGGR-------------- 113
H_WRKY58               GSSTVTSSGVTEAAAAAATTTR----------------RGRGNGKK----AGGGGR-------------- 113
LOC_Os05g45230_WRKY58  GSSTVTSSGVTEAAAAAATTTR----------------RGRGNGKK----AGGGGR-------------- 113
LOC_Os06g44010_WRKY28  RSVDSLDDAAHHRKPSPPFVAAAAAAAYASPDQMECTSAAAAAAAKRVVREDCKPKVSKR---------- 237
H_WRKY28               RSVDSLDDAAHHRKPSPPFVAAAAAAAYASPDQMECTSAAAAAAAKRVVREDCKPKVSKR---------- 237
T_WRKY28               RSVDSLDDAAHHRKPSPPFVAAAAAAAYASPDQMECTSAAAAAAAKRVVREDCKPKVSKR---------- 237
T_WRKY62               ARAPSVSTSCAAREDAAPAVAAAAASTACPSRQQPPTAEPRPKVRTVRVRADAADAT------------- 137
H_WRKY62               -------------------------------------------VRTVRVRADAADAT------------- 14
LOC_Os09g25070_WRKY62  ARAPSVSTSCAAREDAAPAVAAAAASTACPSRQQPPTAEPRPKVRTVRVRADAADAT------------- 134
LOC_Os09g25060_WRKY76  SPLSGKKRSRESMETANSCDANSNRHQGGDADHAESFAADDGTCRRIKVSR-VCRRI------------- 156
H_WRKY76               SPLSGKKRSRESMETANSCDANSNRHQGGDADHAESFAADDGTCRRIKVSR-VCRRI------------- 63
T_WRKY76               T----EKRSRESMETANSCDANSNRHQGGDADHAESFAADDGTCRRIKVSR-VCRRI------------- 125

                                360       370       380       390       400       410       420
                       ....|....|....|....|....|....|....|....|....|....|....|....|....|....|
LOC_Os12g02450_WRKY64  -------------------------------SVSLETPVPHYDGHQWRKYGQKHINNSKHPRSYYRCTYR 174
HWRKY64                -------------------------------SVSLETPVPHYDGHQWRKYGQKHINNSKHPRSYYRCTYR 174
T_WRKY64               -------------------------------SVSLETPVPHYDGHQWRKYGQKHIKNSKHPRSYYRCTYR 173
LOC_Os03g21710_WRKY79  -------------------------------SSNKRVAATLEDGHVWRKYGQKDIQNSPYPRSYYRCTHK 161
H_WRKY79               -------------------------------SSNKRVAATLEDGHVWRKYGQKDIQNSPYPRSYYRCTHK 161
T_WRKY79               -------------------------------SSNKRVAATLEDGHVWRKYGQKDIQNSPYPRSYYRCTHK 161
LOC_Os05g49620_WRKY19  -------------------------------VTSVQDTASLDDGLSWRKYGQKDILGAKYPRAYFRCTHR 134
H_WRKY19               -------------------------------VTSVQDTASLDDGLSWRKYGQKDILGAKYPRAYFRCTHR 147
T_WRKY19               -------------------------------VTSVQDTASLDDGLSWRKYGQKDILGAKYPRAYFRCTHR 147
LOC_Os05g46020_WRKY07  ---------------------ERPR-TERIAFRTKSEIEILDDGYKWRKYGKKSVKNSPNPRNYYRCSTE 163
T_WRKY07               ---------------------ERPR-TERIAFRTKSEIEILDDGYKWRKYGKKSVKNSPNPRNYYRCSTE 163
H_WRKY07               ---------------------ERPR-TERIAFRTKSEIEILDDGYKWRKYGKKSVKNSPNPRNYYRCSTE 159
LOC_Os01g51690_WRKY26  ---------------------DRTT-TDKIAFRTRSDDEILDDGYKWRKYGKKSVKNSPNPRNYYRCSTE 188
H_WRKY26               ---------------------DRTT-TDKIAFRTRSDDEILDDGYKWRKYGKKSVKNSPNPR-YFGTS-- 185
T_WRKY26               ---------------------DRTM-TDKIAFRTRSDDEILDDGYKWRKYGKKSVKNSPNPRNYYRCSTE 177
H_WEKY77               ---------------------PRSKNGSKIAFKTRSEVDVLDDGYRWRKYGKKMVKNSPNPRNYYRCSSE 165
T_WRKY77               ---------------------PRSKNGSKIAFKTRSEVDVLDDGYRWRKYGKKMVKNSPNPRNYYRCSSE 165
LOC_Os01g40260_WRKY77  ---------------------PRSKNGSKIAFKTRSEVDVLDDGYRWRKYGKKMVKNSPNPRNYYRCSSE 165
LOC_Os05g39720_WRKY70  PDSKRWRRDGGDGEGVSLVAGNRTVREPRVVVQTMSDIDILDDGYRWRKYGQKVVKGNPNPRSYYKCTTA 420
H_WRKY70               PDSKRWRRDGGDGEGVSLVAGNRTVREPRVVVQTMSDIDILDDGYRWRKYGQKVVKGNPNPRSYYKCTTA 420
T_WRKY70               PDSKRWRRDGGDGEGVSLVSGNRTVREPRVVVQTMSDIDILDDGYRWRKYGQKVVKGNPNPRSYYKCTTA 420
LOC_Os01g53260_WRKY23  ---------------------KKKASRPRFAFQTRSDNDILDDGYRWRKYGQKAVKNSKHPRSYYRCTHH 204
H_WRKY23               ---------------------KKKASRPRFAFQTRSDNDILDDGYRWRKYGQKAVKNSKHPRSYYRCTHH 204
T_WRKY23               ---------------------KKKASRPRFAFQTRSDNDILDDGYRWRKYGQKAVKNSKHPRSYYRCTHH 204
T_WRKY58               --------------------------TPRFAFHTRSENDILDDGYRWRKYGQKAVKNSDFP--------- 148
H_WRKY58               --------------------------TPRFAFHTRSENDILDDGYRWRKYGQKAVKNSDFP--------- 148
LOC_Os05g45230_WRKY58  --------------------------TPRFAFHTRSENDILDDGYRWRKYGQKAVKNSDFP--------- 148
LOC_Os06g44010_WRKY28  ----------------------------FVHADPSDLSLVVKDGYQWRKYGQKVTKDNPCPRAYFRCSFA 279
H_WRKY28               ----------------------------FVHADPSDLSLVVKDGYQWRKYGQK----------------- 262
T_WRKY28               ----------------------------FVHADPSDLSLVVKDGYQWRKYGQKVTKDNPCPRAYFRCSFA 279
T_WRKY62               --------------------------------DANSMAETVKDGYQWRKYGQKVTRDNPYPRAYFRCAFA 175
H_WRKY62               --------------------------------DANSMAETVKDGYQWRKYGQKVTRDNPYPRAYFRCAFA 52
LOC_Os09g25070_WRKY62  --------------------------------DANSMAETVKDGYQWRKYGQKVTRDNPYPRAYFRCAFA 172
LOC_Os09g25060_WRKY76  --------------------------------DPSDTSLVVKDGYQWRKYGQKVTRDNPSPRAYFRCAFA 194
H_WRKY76               --------------------------------DPSDTSLVVKDGYQWRKYGQKV---------------- 85
T_WRKY76               --------------------------------DPSDTSLVVKDGYQWRKYGQKVTRDNPSPRAYFRCAFA 163
```

```
                                   430       440       450       460       470       480       490
                         ....|....|....|....|....|....|....|....|....|....|....|....|....|....|
LOC_Os12g02450_WRKY64    QEEKCKATKTVQQRED--LHHANSYNGDHP------VMYTVVYYGQHTCCKGP-----AASADDHVVVEA 231
HWRKY64                  QEEKCKATKTVQQRED--LHHANSYNGDHP------IMYTVVYYGQHTCCKGP-----AALADDHVVVEA 231
T_WRKY64                 QEEKCKATKTVQQRED--LQHANSYNGDHP------VMYTVVYYGQHTCCKGP-----VASADDHVVVEA 230
LOC_Os03g21710_WRKY79    LDQGCGARRQTQRCEADPSNYDITYYGEHTCRDPSTIIPTAIANAAGAASDGPNNNIISFATGGVVVANS 231
H_WRKY79                 LDQGCGARRQTQRCEADPSNYDITYYGEHTCRDPSTIIPTAIANAAGAASDGPNNNIISFATGGVVVANS 231
T_WRKY79                 LDQGCGARRQTQRCEADPSNYDITYYGEHTCRDPSTIIPTAIANAAGAASDGPNHNIISFATGGVVVANS 231
LOC_Os05g49620_WRKY19    HTQGCNATKQVQRADGDPLLFDVVYLGDHTCG---QAAVAAAAQSAPPEHAGQ-----EQQRQSSLLAAG 196
H_WRKY19                 HTQGCNATKQVQRADGDPLLFDVVYLGDHTCG---QAAVAAAAQSAPPEHAGQ-----EQQRQSSLLAAG 209
T_WRKY19                 HTQGCNATKQVQRADGDPLLFDVVYLGDHTCG---QAAVTAAAQSAPPEHAGQ-----EQQRQSSLLAAG 209
LOC_Os05g46020_WRKY07    ---GCNVKKRVERDKDDPSYVVTTYEGTHNHVSPSTVYYASQDAA---------SGRFFVAGTQPPGSLN 221
T_WRKY07                 ---GCNVKKRVERDKDDPSYVVTTYEGTHNHVSPSTVYYASQDAA---------SGRFFVAGTQPPGSLN 221
H_WRKY07                 ---GCNVKKRVERDKDDPSYVVTTYEGTHNHVSPSTVYYASQDAA---------SGRFFVAGTQPPGSLN 217
LOC_Os01g51690_WRKY26    ---GCNVKKRVERDKNDPRYVVTMYEGIHNHVCPGTVYYAAQDAA---------SGRFFVAGISHP-DLN 245
H_WRKY26                 ----------------------FVYELVFKFGCF------------------------------------ 197
T_WRKY26                 ---GCNVKKRVERDKNDPRYVVTTYEGIHNHVCPGTVYYAAQDAA---------SGRFFVAGISHP-DLN 234
H_WEKY77                 ---GCRVKKRVERARDDARFVVTTYDGVHN-PAPLHLR--PQLPP---------PGGYSIAGAPAVVAPH 220
T_WRKY77                 ---GCRVKKRVERARDDARFVVTTYDGVHNHPAPLHLR--PQLPP---------PGGYSIAGAPAVVAPH 221
LOC_Os01g40260_WRKY77    ---GCRVKKRVERARDDARFVVTTYDGVHNHPAPLHLR--PQLPP---------PGGYSIAGAPAVVAPH 221
LOC_Os05g39720_WRKY70    ---GCPVRKHVERASNDLRAVITTYEGKHNHDVPAARGSAAAALYRATPPPQASNAGMMPTTAQPSSYLQ 487
H_WRKY70                 ---GCPVRKHVERASNDLRAVITTYEGKHNHDVPAARGSAAAALYRATPPPQASNAGMMPTTAQPSSYLQ 487
T_WRKY70                 ---GCPVRKHVERASNDLRAVITTYEGKHNHDVPAARGSAAAALYRATPPPQASNAGMMLTTAQPSSYLQ 487
LOC_Os01g53260_WRKY23    ---TCNVKKQVQRLAKDTSIVVTTYEGVHNHPCEKLMEALTPILK----------QLQFLSQF------- 254
H_WRKY23                 ---TCNVKKQVQRLAKDTSIVVTTYEGVHNHPCEKLMEALTPILK----------QLQFLSQF------- 254
T_WRKY23                 ---TCNVKKQVQRLAKDTSIVVTTYEGVHNHPCEKLMEALTPILK----------QLQFLSQF------- 254
T_WRKY58                 -------SDDE-LLFSDVDNTQTATENLRFIPLGRVYITG------------------------------ 180
H_WRKY58                 -------SDDELLLFSDVDNTQTATENLRFIPLGRVYITG------------------------------ 181
LOC_Os05g45230_WRKY58    -------SDDELLLFSDVDNTQTATENLRFIPLGRVYITG------------------------------ 181
LOC_Os06g44010_WRKY28    P--ACPVKKKVQRSADDNTVLVATYEGEHNHAQPP----------------------------HHDAGSK 319
H_WRKY28                 ---------------------------------------------------------------------- 262
T_WRKY28                 P--ACPVKKKVQRSADDNTVLVATYEGEHNHAQPP----------------------------HHDAGSK 319
T_WRKY62                 P--SCPVKKKLQRCAEDRSMLVATYEGEHNHALSTQTTEFVASGCTTSQHAGGSSSSPLPCSISINSSGR 243
H_WRKY62                 P--SCPVKKKLQRCAEDRSMLVATYEGEHNHALSTQTTEFVASGCTTSQHAGGSSSSPLPCSISINSSGR 120
LOC_Os09g25070_WRKY62    P--SCPVKKKLQRCAEDRSMLVATYEGEHNHALSTQTTEFVASGCTTSQHAGGSSSSPLPCSISINSSGR 240
LOC_Os09g25060_WRKY76    P--SCPVKKKVQRSAEDSSLLVATYEGEHNHPHPSPRAGELPA-------AAGGAGGSLPCSISINSSGP 255
H_WRKY76                 ---------------------------------------------------------------------- 85
T_WRKY76                 P--SCPVKKKVQRSAEDSSLLVATYEGEHNHPHPSPRAGELPA-------AVGGAGGSLPCSISINSSGP 224

                                   500       510       520       530       540       550       560
                         ....|....|....|....|....|....|....|....|....|....|....|....|....|....|
LOC_Os12g02450_WRKY64    SQISTDSHCQSPGSSSSELQA----------------------------AAHAGDSSQCSNISVTCSSSV 273
HWRKY64                  SQISTDSHCQSPGSSSSELQA----------------------------AAHAGDSSQCSNISVTCSSSV 273
T_WRKY64                 SQISTDSHCQSPGSSSSELQA----------------------------AAHAGDSSQCSNISVTCSSSV 272
LOC_Os03g21710_WRKY79    SRLAREGTTATTTSAATQLSSSWGTSGGGGGGDDVFSSSGERFMQWDELAAAVGHVSSVGVTSSTVGSAP 301
H_WRKY79                 SRLAREGTTATTTSAATQLSSSWGTSGGGGGGDDVFSSSGERFMQWDELAAAVGHVSSVGVTSSTVGSAP 301
T_WRKY79                 SRLAREGTTATTTSAATQLSSSWGTSGGGGGGDDVFSSSGERFMQWDELAAAVGHVSSVGVTSSTVGSAP 301
LOC_Os05g49620_WRKY19    TEGIHQQVVAEPMAAPFLFTS----------------------------TAAGGVDDGYFSFISPANSDC 238
H_WRKY19                 TEGIHQQVVAEPMAAPFLFTS----------------------------TAAGGVDDGYFSFISPANSDC 251
T_WRKY19                 TEGIHQQVVAEPMAAPFLFTS----------------------------TAAGGVDDGYFSFISPANSDC 251
LOC_Os05g46020_WRKY07    ---------------------------------------------------------------------- 221
T_WRKY07                 ---------------------------------------------------------------------- 221
H_WRKY07                 ---------------------------------------------------------------------- 217
LOC_Os01g51690_WRKY26    ---------------------------------------------------------------------- 245
H_WRKY26                 ---------------------------------------------------------------------- 197
T_WRKY26                 ---------------------------------------------------------------------- 234
H_WEKY77                 GRLGLEEAEVIALFRGTTATS----------------------------LLLP----------------- 245
T_WRKY77                 GRLGLEEAEVIALFRGTTATS----------------------------LLLP----------------- 246
LOC_Os01g40260_WRKY77    GRLGLEEAEVIALFRGTTATS----------------------------LLLP----------------- 246
LOC_Os05g39720_WRKY70    GGGGVLPAGGYGASYGGAPTT----------------------------TQPANGGGFAALSGRFDDDAT 529
H_WRKY70                 GGGGVLPAGGYGASYGGAPTT----------------------------TQPANGGGFAALSGRFDDDAT 529
T_WRKY70                 GGGGVLPAGGYGASYGGAPTT----------------------------TQPANGGGFAALSGRFDDDAT 529
LOC_Os01g53260_WRKY23    ---------------------------------------------------------------------- 254
H_WRKY23                 ---------------------------------------------------------------------- 254
T_WRKY23                 ---------------------------------------------------------------------- 254
T_WRKY58                 ---------------------------------------------------------------------- 180
H_WRKY58                 ---------------------------------------------------------------------- 181
LOC_Os05g45230_WRKY58    ---------------------------------------------------------------------- 181
LOC_Os06g44010_WRKY28    TAAAAKHSQHQPPPSAAAAVV----------------------------RQQQEQAAAAGPSTEVAARKN 361
H_WRKY28                 ---------------------------------------------------------------------- 262
T_WRKY28                 TAAAAKHSQHQPPPSAAAAVV----------------------------RQQQEQAAAAGPSTEVAARKN 361
T_WRKY62                 TITLDLTNQAGSGSIASCGVE----------------------------AAAVSGELVT--VLSPELRRH 283
H_WRKY62                 TITLDLTNQAGSGSIASCGVE----------------------------AAAVSGELVT--VLSPELRRH 160
LOC_Os09g25070_WRKY62    TITLDLTNQAGSGSIASCGVE----------------------------AAAVSGELVT--VLSPELRRH 280
LOC_Os09g25060_WRKY76    TITLDLTKNGGAVQVVEAAHP----------------------------PPPPDLKEVCREVASPEFRTA 297
H_WRKY76                 ---------------VEAAHP----------------------------PPPPDLKEVCREVASPEFRTA 112
T_WRKY76                 TITLDLTKNGGAVQVVEAAHP----------------------------PPPPDLKEVCREVASPEFRTA 266
```

```
                                   570       580       590       600       610
                         ....|....|....|....|....|....|....|....|....|....|....|
LOC_Os12g02450_WRKY64    VVEDCNKLLDMLPAADELTTDVLLFDMTAYAP----LDLDINWEMDTNALWA--- 321
HWRKY64                  VVEDCNKLLDMLPAADELTTDVLLFDMTAYAP----LDLDINWEMDTNALWV--- 321
T_WRKY64                 VVEDCNKLLDMLPAADELTTDVLLFDMTAYAP----LDLDINWEMDTNSLWA--- 320
LOC_Os03g21710_WRKY79    AAENDGGNGDTAAGGGGDGGGAGSFPSSPSAGSLGFVVGPLGSIEDVDDFFPFDP 356
H_WRKY79                 AAENDGGNGDTAAGGGGDGGGAGSFPSSPSAGSLGFVVGPLGSIEDVDDFFPFDP 356
T_WRKY79                 AAENDGGNGDTAAGGGGDGGGAGSFPSSPSAGSLGFVVGPLGSIEDVDDFFPFDP 356
LOC_Os05g49620_WRKY19    QFSSDFSAGSVGVDMDHEARFEDLFSST-----------LEFFQSEIQNL----- 277
H_WRKY19                 QFSSDFSAGSVGVDMDHEARFEDLFSST-----------LEFFQSEIQNL----- 290
T_WRKY19                 QFSSDFSAGSVGVDMDHEARFEDLFSST-----------LEFFQSEIQNL----- 290
LOC_Os05g46020_WRKY07    ------------------------------------------------------- 221
T_WRKY07                 ------------------------------------------------------- 221
H_WRKY07                 ------------------------------------------------------- 217
LOC_Os01g51690_WRKY26    ------------------------------------------------------- 245
H_WRKY26                 ------------------------------------------------------- 197
T_WRKY26                 ------------------------------------------------------- 234
H_WEKY77                 ------------------------------------------------------- 245
T_WRKY77                 ------------------------------------------------------- 246
LOC_Os01g40260_WRKY77    ------------------------------------------------------- 246
LOC_Os05g39720_WRKY70    GASYSYTSQQQQQPNDAVYYASRAKDEPRDDGIMSFFEQPLLF------------ 572
H_WRKY70                 GASYSYTSQQQQQPNDAVYYASRAKDEPRDDGIMSFFEQPLLF------------ 572
T_WRKY70                 GASYSYTSQQQQQPNDAVYYASRAKDEPRDDGIMSFFEQPLLF------------ 572
LOC_Os01g53260_WRKY23    ------------------------------------------------------- 254
H_WRKY23                 ------------------------------------------------------- 254
T_WRKY23                 ------------------------------------------------------- 254
T_WRKY58                 ------------------------------------------------------- 180
H_WRKY58                 ------------------------------------------------------- 181
LOC_Os05g45230_WRKY58    ------------------------------------------------------- 181
LOC_Os06g44010_WRKY28    LAEQMAATLTRDPGFKAALVTALSGRILELSPTKN-------------------- 396
H_WRKY28                 ------------------------------------------------------- 262
T_WRKY28                 LAEQMAATLTRDPGFKAALVTALSGRILELSPTKN-------------------- 396
T_WRKY62                 LVEEVVQVLKNDAEFVEAVTNAVAARVVDQIPHIPVHL----------------- 321
H_WRKY62                 LVEEVVQVLKNDAEFVEAVTNAVAARVVDQIPHIPVHL----------------- 198
LOC_Os09g25070_WRKY62    LVEEVVQVLKNDAEFVEAVTNAVAARVVDQIPHIPVHL----------------- 318
LOC_Os09g25060_WRKY76    LVEQMASALTSDPKFTGALAAAILQKLPEF------------------------- 327
H_WRKY76                 LVEQMASALTSDPKFTGALAAAILQKLPEF------------------------- 142
T_WRKY76                 LVEQMASALTSDPKFTGALAAAILQKLPEF------------------------- 296
```

Fig. S3: Structural variations at amino acid level in the three genotypes.

```
                              10        20        30        40        50        60        70
                     ....|....|....|....|....|....|....|....|....|....|....|....|....|....|
LOC_Os01g51690_pW26  --TTATA-TTCTGAGATGAATATAGTATAGCTTATTTCGTATACAACATTATTGGCCCATCTTCAAAATA 67
H289702_pW26         ---------------------------------------------------------------------- 1
T3056296_pW26        --TTATA-AAAACATAACCCAACGAGACAATAAATTTAAAAACTAAAAGTTTTAA---AATTTCTGAGGA 64
LOC_Os01g53260_pW23  CTTCATACTGAATATAAAAATCACGATTAATTTAATGTAGAGACCAGATTAATTTCTACTTTTTCCGTTT 70
H1961965_pW23        CTTCATACTGAATATAAAAATCACGATTAATTTAATGTAGAGACCAGATTAATTTCTACTTTTTCCGTTT 70
T3027906_pW23        CTTCATACTGAATATAAAAATCACGATTAATTTAATGTAGAGACAAGATTAATTTCTACTTTTTCCGTTT 70
LOC_Os06g44010_pW28  ---------------------------------------------------------------------- 1
H1976995_pW28        ---------------------AAAATGATTTTGAACATGTAAGTTCTGTTCATTAGTGCTAAACTGGTCC 49
T3063501_pW28        ----------------------AAATGATTTTGAACATGTAAGTTCTGTTCATTAGTGCTAAACTGGTCC 48
LOC_Os05g46020_pW07  -------------------------------TAACTTTCCTGTCAATAGGTTTGATTAGACTCGGGCAAA 39
H2000561_pW07        -------------------------------TAACTTTCCTGTCAATAGGTTTGATTAGACTCGGGCAAA 39
T3066720_pW07        -------------------------------TAACTTTCCTGTCAATAGGTTTGATTAGGCTCGGGCAAA 39
H2023318_pW64        ---------------------------------------------------------------------- 1
T3095730_pW64        ---------------------------------------------------------------------- 1
LOC_Os12g02450_pW64  ----------------------------------AATTAGTGGTCTAAAACTGAACCGCGTAAAAAGCTC 36
LOC_Os01g40260_pW77  ------------------------------------------CTATGTCATTAAAATAGTTATAAAAGAA 28
T3097007_pW77        ------------------------------------------CTATGTCATTAAAATAGTTATAAAAGAA 28
H1950043_pW77        -------------------------------------------TATGTCATTAAAATAGTTATAAAAGAA 27
LOC_Os09g25070_pW62  -----------------------AACACATAGCACCACACTCATATATCCGCAAATTCCTC-TAAAAGGC 46
H1972014_pW62p       -----------------------AACACATAGCACCACACTCATATATCCGCAAATTCCTC-TAAAAGGC 46
T3091546_pW62        -----------------------AACACATAGCACCACACTCATATATCCGCAAATTCCTC-TAAAAGGC 46
LOC_Os05g39720_pW70  --------------------------TACAACTGTCACTGACATACA--GTCCGAGCAGAC--ACCTGAA 40
H334389_pW70         --------------------------TACAACTGTCACTGACATACA--GTCCGAGCAGAC--ACCTGAA 40
T3094952_pW70        --------------------------TACAACTGTCACTGACATACA--GTCCGAGCAGAC--ACCTGAA 40
LOC_Os05g45230_pW58  ----------------------GAAACGGTCCCGAGGCAGTCCTGCGTCGCCGTTGCGCAT--CACCGAG 46
H1850565_pW58p       ----------------------GAAACGGTCCCGAGGCAGTCCTGCGTCGCCGTTGCGCAT--CACCGAG 46
T3075919_pW58        ----------------------GAAACGGTCCCGAGGCAGTCCTGCGTCGCCGTTGCGCAT--CACCGAG 46
LOC_Os03g21710_pW79  ---------------------------TCTCTCTGTCTGGTACTTTATTAATCACCCTCGT--TGATATA 41
H1952278_pW79        ---------------------------TCTCTCTGTCTGGTACTTTATTAATCACCCTCGT--TGATATA 41
T3070072_pW79        ---------------------------TCTCTCTGTCTGGTACTTTATTAATCACCCTCGT--TGATATA 41
LOC_Os05g49620_pW19  ---------------------------------CAATCAGGCTGGCAAGAAAGAACACTGT---GCCAAG 34
H2014700_pW19        ----------------------------------AATCAGGCTGGCAAGAAAGAACACTGT---GCCAAG 33
T3058779_pW19        ---------------------------------CGTTCCAAAATGTAACAACCTTAGTGGT---CCTGTT 34
LOC_Os09g25060_pW76  -----------------------------GTCTTAGAATATTATAATTTGTTTTAAAAGTTTCACTCATC 41
T3076120_pW76        --------------------------------------------------TTTTAAAAGTTTCACTCATC 20
H1974322_pW76p       -----------------------------GTCTTAGAATATTATAATTTGTTTTAAAAGTTTCACTCATC 41

                              80        90       100       110       120       130       140
                     ....|....|....|....|....|....|....|....|....|....|....|....|....|....|
LOC_Os01g51690_pW26  ACAAAAATATGGAAT-TTTCAAAAAAGAAAGGTTCATCTAAAATATAT-CGATGATAAATGACAATTAAG 135
H289702_pW26         ---------------------------------------------------------------------- 1
T3056296_pW26        CAATGAATAAAAGCG-GTGACAAAATGATAGATCGAACAAATAAATAA-AGAAGGGGATAACAAAAAAAA 132
LOC_Os01g53260_pW23  CAGGTTATAAGACTT-TCTAACAAATTGTATCTAGATTCATTAATAATTATATAAATATGGATAATGTTA 139
H1961965_pW23        CAGGTTATAAGACTT-TCTAACAAATTGTATCTAGATTCATTAATAATTATATAAATATGGATAATGTTA 139
T3027906_pW23        CAAGTTATAAGACTT-TCTAACAAATTATATCTAGATTCATTAACAATTATATAAATATGAATAATGTTA 139
LOC_Os06g44010_pW28  ---------------------------------------------TAACATTTCTATAAAAAAAAA--GA 23
H1976995_pW28        TAGAATACAGTGTTT-TCTTAATATAAAAACTTATTACACTGTACTAACATTTCTATAAAAAAAAA--GA 116
T3063501_pW28        TAGAATACAGTGTTT-TCTTAATATAAAAACTTATTACACTGTACTAACATTTCTATAAAAAAAAAA-GA 116
LOC_Os05g46020_pW07  GCAGGAACACACCTGATCCAAGACCCGATACCTCTTGGACAATCGGAGCACGATAGTATTAGCAGGCAAA 109
H2000561_pW07        GCAGGAACACACCTGATCCAAGACCCGATACCTCTTGGACAATCGGAGCACGATAGTATTAGCAGGCAAA 109
T3066720_pW07        GCAGGAACACACCTGATCCAAGACCCGATACCTCTTGGACAATCGGAGCACGATAGTATTAGCAGGCAAA 109
H2023318_pW64        ---------------------------------------------------------------------- 1
T3095730_pW64        ---------------------------------------------------------------------- 1
LOC_Os12g02450_pW64  AGGTGATTAATTAGAACTTGGTGTTGAACGTACTACCTGATGATCTATCAAGCTAGCACTAAGAAACTGA 106
LOC_Os01g40260_pW77  CTGAAAAAAATTGAAAGAT--GAATTAATATGTGATATATCATTCCACAAGTATGCAACTTAAATTCA-- 94
T3097007_pW77        CTGAAAAAAATTGAAAGAT--GAATTAATATGTGATATATCATTCCACAAGTATGCAACTTAAATTCA-- 94
H1950043_pW77        CTGAAAAAAATTGAAAGAT--GAATTAATATGTGATATATCATTCCACAAGTATGCAACTTAAATTCA-- 93
LOC_Os09g25070_pW62  TAAAACACACTAGAAAGGACGAAACCAACGACACATTCTTAATCTCATTAAAATTCTATGAAAGCCCCCT 116
H1972014_pW62p       TAAAACACACTAGAAAGGACGAAACCAACGACACATTCTTAATCTCATTAAAATTCTATGAAAGCCCCCT 116
T3091546_pW62        TAAAACACACTAGAAAGGACGAAACCAACGACACATTCATAATCTCATTAAAATTCTATGAAAGCCCCCT 116
LOC_Os05g39720_pW70  ATG--CAAATTTCAACAGTAGAATCAGTCAGCTGATTTAA-ATATAAACAGGGGTAAGAATTTAAGCA-- 105
H334389_pW70         ATG--CAAATTTCAACAGTAGAATCAGTCAGCTGATTTAA-ATATAAACAGGGGTAAGAATTTAAGCA-- 105
T3094952_pW70        ATG--CAAATTTCAACAGTAGAATCAGTCAGCTGATTTAA-ATATAAACAGGGGTAAGAATTTAAGCA-- 105
LOC_Os05g45230_pW58  GCGGCCCGACGTCACCGTTGCACGCCATTGACGCGTTCCT-GTGTAGCCGTTGTGCGGCACCGACACT-- 113
H1850565_pW58p       GCGGCCCGACGTCACCGTTGCACGCCATTGACGCGTTCCT-GTGTAGCCGTTGTGCGGCACCGACACT-- 113
T3075919_pW58        GCGGCCCGACGTCACCGTTGCACGCCACTGACGCGTTCCT-GTGTAGCCGTTGTGCGGCACCGACACT-- 113
LOC_Os03g21710_pW79  CTTGTTCTTATATCGATTCTAATATTTCTGTCCTACGTGGAGGAAAG--AGACACCTGTGTGACACGTCG 109
H1952278_pW79        CTTGTTCTTATATCGATTCTAATATTTCTGTCCTACGTGGAGGAAAG--AGACACCTGTGTGACACGTCG 109
T3070072_pW79        CTTGTTCTTATATCGATTCTAATATTTCTGTCCTACGTGGAGGAAAG--AGACACCTGTGTGACACGTCG 109
LOC_Os05g49620_pW19  CTAATTCAC-TGTCCAAGCTGATAATACTTATCATTTTGAAAAAAACACAGTCTTTAAAAAATAATTTTG 103
H2014700_pW19        CTAATTCAC-TGTCCAAGCTGATAATACTTATCATTTTGAAAAAAACACAGTCTTTAAAAAATAATTTTG 102
T3058779_pW19        CGAATCTAC-CGCAAGGATGAAGATTAAGTGTGCGCGCAAAACGAGATAAGTCATTAGCATATGATTAAT 103
LOC_Os09g25060_pW76  TTCCTTTATCGATCTAAACAAGAACATGCTTTTTTTTTTTTACTTTGGTAAGTATTTGTGATAATCATGG 111
T3076120_pW76        TTCCTTTATCGATCTAAACAAGAACATGCTTTTTTTTT--TACTTTGGTAAGTATTTGTGATAATCATGG 88
H1974322_pW76p       TTCCTTTATCGATCTAAACAAGAACATGCTTTTTTTTTTTTACTTTGGTAAGTATTTGTGATAATCATGG 111
```

```
                        150       160       170       180       190       200       210
                    ....|....|....|....|....|....|....|....|....|....|....|....|....|....|
LOC_Os01g51690_pW26 AGGGGAGAAGCAACATGCTTATAATGGAGTAAGCGTAGGCCTATAAACCCCAATGATAATCATATTTGA- 204
H289702_pW26        ---------------------------------------------------------------------- 1
T3056296_pW26       TAAGGGTTAGCAACTGGTGCGACTTTTAAAAACTATAAAATTAGAAATATGTGGATGATAAGGTTTGGT- 201
LOC_Os01g53260_pW23 AAAAGTCTTATAACCTGAAACAGAGGTAATAGTAAATATCGACGATGCACGTATAATCTCTAGCTCGTA- 208
H1961965_pW23       AAAAGTCTTATAACCTGAAACAGAGGTAATAGTAAATATCGACGATGCACGTATAATCTCTAGCTCGTA- 208
T3027906_pW23       AAAAATCTTATAACCTGAAACGGAGGTAGTAGTAAATATCGACGATGCACGTATAATCTCTAGCTCGTA- 208
LOC_Os06g44010_pW28 GAAGAACTTTTTCTCAGAGACAGCATCAATAAGACAAAAAAAAGTTACATGGGAAAAAGAGAAAACACA- 92
H1976995_pW28       GAAGAACTTTTTCTCAGAGACAGCATCAATAAGACAAAAAAAAGTTACATGGGAAAAAGAGAAAACACA- 185
T3063501_pW28       GAAGAACTTTTTCTCAGAGACAGCATCAATAAGACAAAAAAAAGTTACATGGGAAAAAGAGAAAACACA- 185
LOC_Os05g46020_pW07 AATCAACGCCCACGTCGGTGTCGGGTGCGTATTTAAAAACATCGATA---ATATATGCATGGCTAGATA- 175
H2000561_pW07       AATCAACGCCCACGTCGGTGTCGGGTGCGTATTTAAAAACATCGATA---ATATATGCATGGCTAGATA- 175
T3066720_pW07       AATCAACGCCCACGTCGGTGTCGGGTGCGTATTTAAAAACATCGATA---ATATATGCATGGCTAGATA- 175
H2023318_pW64       ------------------------------TACTCCCATCCTACATAGACAACTATGTAGTACAATGTGG 42
T3095730_pW64       ---------------------------------------------------------------------- 1
LOC_Os12g02450_pW64 GGCCCTGTTTAGATGGGACTAAAACTTTTAAGTCCCTATCACATCGGATGTTTGAAAATTAATTATAAAT 176
LOC_Os01g40260_pW77 -ACTTAAAATTTGTTTTTTCCTGTGACATATAGAAGTTGAATTTTCAGTTGCATAATTATGCAGTGATAT 163
T3097007_pW77       -ACTTAAAATTTGTTTTTTCCTGTGACATATAGAAGTTGAATTTTCAGTTGCATAATTATGCAGTGATAT 163
H1950043_pW77       -ACTTAAAATTTGTTTTTTCCTGTGACATATAGAAGTTGAATTTTCAGTTGCATAATTATGCAGTGATAT 162
LOC_Os09g25070_pW62 TGCTTGTGACCAATATTCAGGTACAGAATCAATGATGTACATCCACA-TCACCTTAACCTTCTTAGATTT 185
H1972014_pW62p      TGCTTGTGACCAATATTCAGGTACAGAATCAATGATGTACATCCACA-TCACCTTAACCTTCTTAGATTT 185
T3091546_pW62       TGCTTGTGACCAATATTCAGGTACAGAATCAATGATGTACATCCACA-TCACCTTAACCTTCTTAGATTT 185
LOC_Os05g39720_pW70 -GCTAAAAAGCGATACAAACCCTAATTAACTATACTCTACTAATTAAGCTACCCAACCAT-CTGACATGG 173
H334389_pW70        -GCTAAAAAGCGATACAAACCCTAATTAACTATACTCTACTAATTAAGCTACCCAACCAT-CTGACATGG 173
T3094952_pW70       -GCTAAAAAGCGATACAAACCTTAATTAACTATACTCTACTAATTAAGCTACCCAACCAT-CTGACATGG 173
LOC_Os05g45230_pW58 -GCCCCAC-GTGTTCCTGCCCTCCATCA-CGTTGCCGCAC-ACCCGGGTCATGGCGCCGCACTTCCATGC 179
H1850565_pW58p      -GCCCCAC-GTGTTCCTGCCCTCCATCA-CGTTGCCGCAC-ACCCGGGTCATGGCGCCGCACTTCCATGC 179
T3075919_pW58       -GCCCCAC-GTGTTCCTGCCCTCCATCA-CGTTGCCGCAC-ACCCGGGTCATGGCGCCGCACTTCCACGC 179
LOC_Os03g21710_pW79 TCCCGTGTAGCCA--AAACCACTGGCCGTTCGGTCTCGGTGTTTCCAGTTTCCATCAACCGTAAGCGTGT 177
H1952278_pW79       TCCCGTGTAGCCA--AAACCACTGGCCGTTCGGTCTCGGTGTTTCCAGTTTCCATCAACCGTAAGCGTGT 177
T3070072_pW79       TCCCGTGTAGCCA--AAACCACTGGCCGTTCGGTCTCGGTGTTTCCAGTTTCCATCAACCGTAAGCGTGT 177
LOC_Os05g49620_pW19 ACCATTTTT-TTATTATAATAT-AAAAATATCAATAAATATCCTATTCCAGTGAAGCATTTTTAACACTT 171
H2014700_pW19       ACCATTTTT-TTATTATAATAT-ANNNNNNNNNNNNNNNNNNNNNNNNNNNNNNNNNNTTTTTAACACTT 170
T3058779_pW19       TAAATTTTAATTATTAAAATCTTAAAAATGGATTTATTAGAATTTTTATAGTAACTTTTATATAGAAAGT 173
LOC_Os09g25060_pW76 CGATGATGTGAACACGCGTGCCGAACGTAAAACTATCATTTTTTTTACTTAGTAAAGACAACAAAACCAT 181
T3076120_pW76       CGATGATGTGAACACGCGTGCCGAACGTAAAACTATCATTTTTTTTACTTAGTAAAGACAACAAAACCAT 158
H1974322_pW76p      CGATGATGTGAACACGCGTGCCGAACGTAAAACTATCATTTTTTTTACTTAGTAAAGACAACAAAACCAT 181

                        220       230       240       250       260       270       280
                    ....|....|....|....|....|....|....|....|....|....|....|....|....|....|
LOC_Os01g51690_pW26 TTTTAAAA-TCTTAAGGA---ATTCAAGCAATTTAAAATTCGAATTTCGACCGCTGAAATGGTG-AGAAT 269
H289702_pW26        ---------------------------------------------------------------------- 1
T3056296_pW26       TTTTCAAA-GTATTAAGA---TAATAAGATAGCTATTTAACAAATTTTAAATAAAATCATATTA-AAAAA 266
LOC_Os01g53260_pW23 AATAAATTTGCATGAACA---GTTGGATATATTAATTAACCTGCTGCTGAGTGCTGGCATTTTGCACGAA 275
H1961965_pW23       AATAAATTTGCATGAACA---GTTGGATATATTAATTAACCTGCTGCTGAGTGCTGGCATTTTGCACGAA 275
T3027906_pW23       AATAAATTTGCATGAACA---GTTGGATATATTAATTAACCTGCTGCTGAGTGCTGGCATTTTGCACGAA 275
LOC_Os06g44010_pW28 ACTAAAAAGAGAAAAACACATATTACCACAATTAACTATTCTCTCTGTTCCTGAAGACATACTTCATTTT 162
H1976995_pW28       ACTAAAAAGAGAAAAACACATATTACCACAATTAACTATTCTCTCTGTTCCTGAAGACATACTTCATTTT 255
T3063501_pW28       ACTAAAAAGAGAAAAACACATATTACCACAATTAACTATTCTCTCTGTTCCTGAAGACATACTTCATTTT 255
LOC_Os05g46020_pW07 ACTAAACAGAGATTATGA---ATCAGCACATATGATTTGGGAGCATCATGTCAGACATGTCGAACACATG 242
H2000561_pW07       ACTAAACAGAGATTATGA---ATCAGCACATATGATTTGGGAGCATCATGTCAGACATGTCGAACACATG 242
T3066720_pW07       ACTAAACAGAGATTATGA---ATCAGCACATATGATTTGGGAGCATCATGTCAGACATGTCGAACACATG 242
H2023318_pW64       AGTGGAGTTGAATATACAACTGTAGAATTCACTAGTAGCTAGTATAGTTCTAGTAGAGAATTAATAGTAG 112
T3095730_pW64       ---------------------------------------------------------------------- 1
LOC_Os12g02450_pW64 ATTAAACGTAGACTATTAATAAAACCCATCCATAATCTTGGACTAATTTACGAGACGAATCTAATGAGCC 246
LOC_Os01g40260_pW77 ATCA-TAT------ACTATATCTTAATACATTTTTCTCTCTTTTTTT-TAATTTTTTCATAACTATTTGT 225
T3097007_pW77       ATCA-TAT------ACTATATCTTAATACATTTTTCTCTCTTTTTTT-TAATTTTTTCATAACTATTTGT 225
H1950043_pW77       ATCA-TAT------ACTATATCTTAATACATTTTTCTCTCTTTTTTT-TAATTTTTTCATAACTATTTGT 224
LOC_Os09g25070_pW62 ACCGCTCT------AACATCCCACATACCGGATTAAAGTCATCATTT-AAAAGTGTATTGCGGGGATTGA 248
H1972014_pW62p      ACCGCTCT------AACATCCCACATACCGGATTAAAGTCATCATTT-AAAAGTGTATTGCGGGGATTGA 248
T3091546_pW62       ACCGCTCT------AACATCCCACATACCGGATTAAAGTCATCATTT-AAAAGTGTATTGCGGGGATTGA 248
LOC_Os05g39720_pW70 TTTACTACTTCAGAAGGCAGCTCCCCAATTCATTAAAAAAACCACTAGTAATTTAATTATAGCAAATTTT 243
H334389_pW70        TTTACTACTTCAGAAGGCAGCTCCCCAATTCATTAAAAAAACCACTAGTAATTTAATTATAGCAAATTTT 243
T3094952_pW70       TTTACTACTTCAGAAGGCAGCTCCCCAATTCATTAAAAAAACCACTAGTAATTTAATTATAGCAAATTTT 243
LOC_Os05g45230_pW58 CGCAGGAC--CAAAGCGGTGCCGCACGGCCGTCCTGCGCAACCACTAGTCCCACGGTATGGAAGAGCGGC 247
H1850565_pW58p      CGCAGGAC--CAAAGCGGTGCCGCACGGCCGTCCTGCGCAACCACTAGTCCCACGGTATGGAAGAGCGGC 247
T3075919_pW58       CGCGGGAC--CAAAGCGGTGCCGCACGGTCGTCCTGCGCAACCACCAGTCCCGCGGT------------C 235
LOC_Os03g21710_pW79 CATTCAACGCGCCAATGTATACTAATGAATCTTTATTTTGTGTCTGCATCATGCATTCATGCTTGTGTAC 247
H1952278_pW79       CATTCAACGCGCCAATGTATACTAATGAATCTTTATTTTGTGTCTGCATCATGCATTCATGCTTGTGTAC 247
T3070072_pW79       CATTCAACGCGCCAATGTATACTAATGAATCTTTATTTTGTGTCTGCATCATGCATTCATGCTTGTGTAC 247
LOC_Os05g49620_pW19 GTTCAAA-ACAACAAC-TAGTATTATCAACCCGCATGAAGTAGC-AAGAATCCTATG-ATCAATAC---T 234
H2014700_pW19       GTTCAAA-ACAACAAC-TAGTATTATCAACCCGCATGAAGTAGC-AAGAATCCTATG-ATCAATAC---T 233
T3058779_pW19       TTTCAAACACACCATT-TAGTTTGAAAAACGTGCTAACGGAAACCAAGAAAAAAATGTATCTTAAT---T 239
LOC_Os09g25060_pW76 CCGTGGGTATTAAGAGCAAGTACAATTGTAAAGATATAGCTTAGCTACAAGCACATATGAAAAAGGAAGA 251
T3076120_pW76       CCGTGGGTATTAAGAGCAAGTACAATTGCAAAGATATAGCTTAGCTACAAGCACATATGAAAAAGGGAGA 228
H1974322_pW76p      CCGTGGGTATTAAGAGCAAGTACAATTGTAAAGATATAGCTTAGCTACAAGCACATATGAAAAAGGAAGA 251
```

```
                          290       300       310       320       330       340       350
                  ....|....|....|....|....|....|....|....|....|....|....|....|....|....|
LOC_Os01g51690_pW26  CTTACCCTTTAACCTTAAT--ACTCCCTC-CCTTTCAAATTATAAGAC-GTTTTAACTTTGGTT--CAAG 333
H289702_pW26         ---------------------------------------------------------------------- 1
T3056296_pW26        TAGATAATTTCGTATGGGT--ACTAGCCG-CAATATTGCGCTGGCTAC-CAGCTAGTTTTTTTAGATAAT 332
LOC_Os01g53260_pW23  CGTGTGCATGCACCTGACCCCACCAAACG-CAAAGCTACCATTGCATC-GACC--GCCTCCATCGGCCAT 341
H1961965_pW23        CGTGTGCATGCACCTGACCCCACCAAACG-CAAAGCTACCATTGCATC-GACC--GCCTCCATCGGCCAT 341
T3027906_pW23        CGTGTGCATGCACCTGACCCCACCAAACG-CAAAGCTACCATTGCATC-GACC--GCCTCCATCGGCCAT 341
LOC_Os06g44010_pW28  TTTCATATTTTAAGTCGTTTTAGTTTTTT-CTAATTAAACTTTTTTAA-GTTTGATGAATTTTGAAGAAA 230
H1976995_pW28        TTTCATATTTTAAGTCGTTTTAGTTTTTT-CTAATTAAACTTTTTTAA-GTTTGATGAATTTTGAAGAAA 323
T3063501_pW28        TTTCATATTTTAAGTCGTTTTAGTTTTTT-CTAATTAAACTTTTTTAA-GTTTGATGAATTTTGAAGAAA 323
LOC_Os05g46020_pW07  CCCGTGCCTATCGAACACAGTATCCGTGGGTAAAATCGCTATCGCTGCTCAGTCCAAAGTAGACGGAGCT 312
H2000561_pW07        CCCGTGCCTATCGAACACAGTATCCGTGGGTAAAATCGCTATCGCTGCTCAGTCCAAAGTAGACGGAGCT 312
T3066720_pW07        CCCGTGCCTATCGAACACAGTATCCGTGGGTAAAATCGCTATCGCTGCTCAGTCCAAAGTAGACGGAGCT 312
H2023318_pW64        TAAT-AGTTTAGGTGCAATCTATATA----TATACCACACGGTTG--GAGTATAATCTGCGTAGCGATTC 175
T3095730_pW64        --------------------TATATA----TATACCACACGGTTG--GAGTATAATCTGCGTAGTGATTC 44
LOC_Os12g02450_pW64  TAATTAATCCATGATTAGCCTATGTGATGCTACAGTAAACATTCTCTAATTATAGATTAATTAGGCTTAA 316
LOC_Os01g40260_pW77  GTGACGTACTCCCTCCGTC--C----AAAAAAAGACAAACCCTGAGTTTT-CGTGTCCAACGTTTGATTG 288
T3097007_pW77        GTGACGTACTCCCTCCGTC--C----AAAAAAAGACAAACCCTGAGTTTT-CGTGTCCAACGTTTGATTG 288
H1950043_pW77        GTGACGTACTCCCTCCGTC--C----AAAAAAAGACAAACCCTGAGTTTT-CGTGTCCAACGTTTGATTG 287
LOC_Os09g25070_pW62  ACTAAATCCTGATTTGGTA--T----TGACACAAACATATACGAAAAAGAACATATTGGACATCACGTTG 312
H1972014_pW62p       ACTAAATCCTGATTTGGTA--T----TGACACAAACATATACGAAAAAGAACATATTGGACATCACGTTG 312
T3091546_pW62        ACTAAATCCTGATTTGGTA--T----TGACACAAACATATACGAAAAAGAACATATTGGACATCACGTTG 312
LOC_Os05g39720_pW70  GCTACGCTACTTCGTGGTT--C----TATATGTAAGACACCACACGAAGTAAATTTTAAGAAAAAAAATA 307
H334389_pW70         GCTACGCTACTTCGTGGTT--C----TATATGTAAGACACCACACGAAGTAAATTTTAAGAAAAAAAATA 307
T3094952_pW70        GCTACGCTACTTCGTGGTT--C----TATATGTAAGACACCGCATGAAGTAAATTTTAAGAAAAAAAATA 307
LOC_Os05g45230_pW58  ACCACCTTAATTCTGCGTC--CCTGTTATACAGTAGAGGTATATTTATGCAAATATTTTTCATGCAGGTA 315
H1850565_pW58p       ACCACCTTAATTCTGCGTC--CCTGTTATACAGTAGAGGTATATTTATGCAAATATTTTTCATGCAGGTA 315
T3075919_pW58        ACCATCTTAATTCTGCGTC--CCTATTATACAGTAGAGGTATATTTGTGCAAATATTTTTCATGTAGGTA 303
LOC_Os03g21710_pW79  AGGAGGAGACCGATCCATTACGTACGTATAAAGAGAAGATGAAGCTACAAT---ATATGCAGCTCGATCG 314
H1952278_pW79        AGGAGGAGACCGATCCATTACGTACGTATAAAGAGAAGATGAAGCTACAAT---ATATGCAGCTCGATCG 314
T3070072_pW79        AGGAGGAGACCGATCCATTACGTACGTATAAAGAGAAGATGAAGCTACAAT---ATATGCAGCTCGATCG 314
LOC_Os05g49620_pW19  AAAT-AATATTCCTTCGTT-CTAAAATATA-ACAACCTTAGTGGTCCTATTCGAATCTACCGTAAGAATG 301
H2014700_pW19        AAAT-AATATTCCTTCGTT-CTAAAATATA-ACAACCTTAGTGGTCCTATTCGAATCTACCGTAAGAATG 300
T3058779_pW19        AGTTTAGAACGGGGCCATTATTAGATTAGATATAATACTAAGGGTG-TGTTCGGACCTCT----AGGTTC 304
LOC_Os09g25060_pW76  AAAAAACAGACCATAAATT--TGTAGTGTGAGAGATGAGCCAAGTATTAATGATTCAATGTAT------- 312
T3076120_pW76        AAAAAACAGACCATAAATT--TGTAGTGTGAGAGATGAGCCAAGTATTAATGATTCAATGTATGTTTATA 296
H1974322_pW76p       AAAAAACAGACCATAAATT--TGTAGTGTGAGAGATGAGCCAAGTATTAATGATTCAATGTAT------- 312

                          360       370       380       390       400       410       420
                  ....|....|....|....|....|....|....|....|....|....|....|....|....|....|
LOC_Os01g51690_pW26  TCAAACTGT---TTCAAATTTGTAGAAAATAATAATAATATTTTTAGCTCAACACAAATTTATTATGAAA 400
H289702_pW26         ---------------------------------------------------------------------- 1
T3056296_pW26        GAGAAGTAT---TTTCAATCGGTAGATACATGCCGCTCCCTCTGTTTCAACATATAAACATTTCTAGGAT 399
LOC_Os01g53260_pW23  TCAACCTGC---AAGCGCTCGACCGGTCATCATTCATCAGCTAAAAACTGCAAAAGACGACCCAAGAGAA 408
H1961965_pW23        TCAACCTGC---AAGCGCTCGACCGGTCATCATTCATCAGCTAAAAACTGCAAAAGACGACCCAAGAGAA 408
T3027906_pW23        TCAACCTGC---AAGCGCTCGACCGGTCATCATTCATCAGCTAAAAACTGCAAAAGACGACCCAAGAGAA 408
LOC_Os06g44010_pW28  AATATAGCA---ATGTTTTCAATATAAAACAAATATGCTATCAAAA--TATATTTAATATTTAATGAAAC 295
H1976995_pW28        AATATAGCA---ATGTTTTCAATATAAAACAAATATGCTATCAAAA--TATATTTAATATTTAATGAAAC 388
T3063501_pW28        AATATAGCA---ATGTTTTCAATATAAAACAAATATACTATCAAAA--TATATTTAATATTTAATGAAAC 388
LOC_Os05g46020_pW07  AAAATAAAA---TAAAGAGAGATGCCACTCATTTAATTTGTTTTATTTTAGATCGAATTTAAACTGATAT 379
H2000561_pW07        AAAATAAAA---TAAAGAGAGATGCCACTCATTTAATTTGTTTTATTTTAGATCGAATTTAAACTGATAT 379
T3066720_pW07        AAAATAAAA---TGAAGAGAGATGCCACTCATTTATTTTGTTTTATTTTAGATCGAATTTAAACTGATAT 379
H2023318_pW64        ATGACAAAT-TAGTGGTCTAAAACTGAACCGCGTAAAAAGCTCAGGTGATTAATTAGAACTTGGTGTTG- 243
T3095730_pW64        ATGACAAAT-TAGTGGTCTAAAACTGAACCGCGTAAAAAGCTCAGGTGATTAATTAGAACTTGGTGTTG- 112
LOC_Os12g02450_pW64  AAAATTTGTCTCGTGAATTAGCTTTCATTTATGTAATTAGTTTTG-TAAGTAGTCTATATTTAATACTCT 385
LOC_Os01g40260_pW77  TTCGTTTTAT--------ATGAAATTTTTTATAATTAGTATTTTCATTGTTGTTAGATAATAAAACATGA 350
T3097007_pW77        TTCGTTTTAT--------ATGAAATTTTTTATAATTAGTATTTTCATTGTTGTTAGATAATAAAACATGA 350
H1950043_pW77        TTCGTTTTAT--------ATGAAATTTTTTATAATTAGTATTTTCATTGTTGTTAGATAATAAAACATGA 349
LOC_Os09g25070_pW62  TAAGCTCGATGTCAATGAATTAAGTTTTCTGTAAACGTCAGTCT-GTCATCTTTAAAGCACACAGCACAG 381
H1972014_pW62p       TAAGCTCGATGTCAATGAATTAAGTTTTCTGTAAACGTCAGTCT-GTCATCTTTAAAGCACACAGCACAG 381
T3091546_pW62        TAAGCTCGATGTCAATGAATTAAGTTTTCTGTAAACGTCAGTCT-GTCATCTTTAAAGCACACAGCACAG 381
LOC_Os05g39720_pW70  CTCTATAAATAT-GATTATTTACTAGATGTACAATATCGTTTAGTGTTACCATAAAAATACCAGGAGCAA 376
H334389_pW70         CTCTATAAATAT-GATTATTTACTAGATGTACAATATCGTTTAGTGTTACCATAAAAATACCAGGAGCAA 376
T3094952_pW70        CCCTATAAATAT-GATTATTTACTAGATGTACAATATCGTTTAGTGTTACCATAAAAATACCAGGAGCAA 376
LOC_Os05g45230_pW58  ---TATAAATGC-AAAATATTAATGAAAATATATATTTTCTTTTTTTTTCTTGTTAAGTACTCGACAGGA 381
H1850565_pW58p       ---TATAAATGC-AAAATATTAATGAAAATATATATTTTCTTTTTTTTTCTTGTTAAGTACTCGACAGGA 381
T3075919_pW58        ---TATAAACGC-AAAATATTAATGAAAATATATATTTTCTTTTTTTTTCTTGTTAAGTACTCGACAGGG 369
LOC_Os03g21710_pW79  ATCGCTAGCTGCTATCGGCTAGCGACAGGGCTCGAGTTCGATGTCATCAGTGA----TCCAATGTTAGAT 380
H1952278_pW79        ATCGCTAGCTGCTATCGGCTAGCGACAGGGCTCGAGTTCGATGTCATCAGTGA----TCCAATGTTAGAT 380
T3070072_pW79        ATCGCTAGCTGCTATCGGCTAGCGACAGGGCTCGAGTTCGATGTCATCAGTGA----TCCAATGTTAGAT 380
LOC_Os05g49620_pW19  AAGATTAAGTGTGCGCGCAAAACGAG-ATAAGTCATTAGCATATGATTAATTAAATTTTAATTATTAAAA 370
H2014700_pW19        AAGATTAAGTGTGCGCGCAAAACGAG-ATAAGTCATTAGCATATGATTAATTAAATTTTAATTATTAAAA 369
T3058779_pW19        CCAATCCAAATCCCTCGTTTTTCGCGCGCACGTTTTTCAAAC-TGCTAAACGGTGCGTTTTTTATAAAAA 373
LOC_Os09g25060_pW76  -------------AGTAGACTATTAAAGTGACTGTAGATGATGTATTAATTTTGAAAGGTAGCAGTGGGT 369
T3076120_pW76        TAAACTATTGTATAGTAGACTATTAAAGTGACTGTAGATGATGTATTAATTTTGAAAGGTAGCAGTGGGT 366
H1974322_pW76p       -------------AGTAGACTATTAAAGTGACTGTAGATGATGTATTAATTTTGAAAGGTAGCAGTGGGT 369
```

```
                         430       440       450       460       470       480       490
                     ....|....|....|....|....|....|....|....|....|....|....|....|....|....|
LOC_Os01g51690_pW26  ATATATTCAATTATTAATTTAA-----TGAAACTAT-TT--ATAAA-TATTACTATA-----TTTATGCA 456
H289702_pW26         -----------------------------------------ATAAA-TATTACTATA-----TTTATGCA 23
T3056296_pW26        TAAATTTTATACATAATACAAG-----TATTTCTATGTC--ACCAA-TTCCAATATACGAAATCTATACA 461
LOC_Os01g53260_pW23  GAAACGATCTAGAACGAACC-G-----TGCCGTCCTGCC--GTGAA-TCAGAACTTTTCCTAGTTA-ACT 468
H1961965_pW23        GAAACGATCTAGAACGAACC-G-----TGCCGTCCTGCC--GTGAA-TCAGAACTTTTCCTAGTTA-ACT 468
T3027906_pW23        GAAACGATCTAGAACGAACC-G-----TGCCGTCCTGCC--GTGAA-TCAGAACTTTTTCTAGTTA-ACT 468
LOC_Os06g44010_pW28  TAATTTTATTTTGTAGATGTTG-----CTAATTTTCTCT--GTAAA-CTTAATTAAAT----CTTAAAAA 353
H1976995_pW28        TAATTTTATTTTGTAGATGTTG-----CTAATTTTCTCT--GTAAA-CTTAATTAAAT----CTTAAAAA 446
T3063501_pW28        TAATTTTATTTTGTAGATGTTG-----CTAATTTTTTCT--GTAAA-CTTAATTAAAT----CTTAAAAA 446
LOC_Os05g46020_pW07  AAGCGTCTGAGTTTAAATTAAG-----TGAGTGCATATTTAATGAA-AATAAATAAACTATATCTAAAAA 443
H2000561_pW07        AAGCGTCTGAGTTTAAATTAAG-----TGAGTGCATATTTAATGAA-AATAAATAAACTATATCTAAAAA 443
T3066720_pW07        AAGTGTCTGAGTTTAAATTAAG-----TAAGTGTATATTTGGTGAA-AATAAACGAACTATATCTAAAAA 443
H2023318_pW64        AACGTACTACCTGATGATCTA------TCAAGCTAGCACTAAGGA---AGAAACTGAGTCCATGCATATA 304
T3095730_pW64        AACGTACTACCTGATGATCTA------TCAAGCTAGCACTAAGGA---AGAAACTGAGTCCATGCATATA 173
LOC_Os12g02450_pW64  AAATTAGTGTCTAAAGACAGGGA---CTAAAGTTAAGTCCCTGGATCTAAACACCACCTGAGTCCATATA 452
LOC_Os01g40260_pW77  TTAA--TACTTTAT----------------GCGTGACTTGTCTT----TTTAATTTTTTTTCATAGTT-- 396
T3097007_pW77        TTAA--TACTTTAT----------------GCGTGACTTGTCTT----TTTAATTTTTTTTCATAGTT-- 396
H1950043_pW77        TTAA--TACTTTAT----------------GCGTGACTTGTCTT----TTTAATTTTTTTTCATAGTT-- 395
LOC_Os09g25070_pW62  TAAAGATACTTCCTCTCTTTTAGAAAAAAGGCAAATCCTGACTACGTATCTAAAGATAATACATGCCTAA 451
H1972014_pW62p       TAAAGATACTTCCTCTCTTTTAGAAAAAAGGCAAATCCTGACTACGTATCTAAAGATAATACATGCCTAA 451
T3091546_pW62        TAAAGATATTTCCTCTCTTTTAAAAAAAAGGCAAATCCTGACTAAATATATAAACATAATACATGCCTAA 451
LOC_Os05g39720_pW70  TTTGGTCACACCATGT--------TATAAAGCAGGTCCTATCTTGTATCCTAGAAACACTAAATTAAT-- 436
H334389_pW70         TTTGGTCACACCATGT--------TATAAAGCAGGTCCTATCTTGTATCCTAGAAACACTAAATTAAT-- 436
T3094952_pW70        TTTGGTCACACCATGT--------TATAAAGCAGGTCCTATCTTGTATCCTAGAAACACTAAATTAAT-- 436
LOC_Os05g45230_pW58  TCAAACGGACGCAATT--------GACCACGCATGACACATATAAAACCAACACTACGTGAACAAAAA-- 441
H1850565_pW58p       TCAAACGGACGCAATT--------GACCACGCATGACACATATAAAACCAACACTACGTGAACAAAAA-- 441
T3075919_pW58        TCAAACGGACGCAATT--------GACCACGCATGACACATATAAAACCAACACTACGTGAACAAAAA-- 429
LOC_Os03g21710_pW79  GCAACGAAAGCAAC---ATGTGTACACTAAAAGAGACGAAATTTCTATCCAAAGTAG--ATGCGGACAAC 445
H1952278_pW79        GCAACGAAAGCAAC---ATGTGTACACTAAAAGAGACGAAATTTCTATCCAAAGTAG--ATGCGGACAAC 445
T3070072_pW79        GCAACGAAAGCAAC---ATGTGTACACTAAAAGAGACGAAATTTCTATCCAAAGTAG--ATGCGGACAAC 445
LOC_Os05g49620_pW19  TCTTAAAAATGGAT-TTATTAGAATTTTTATAGTAAC---TTTTATATAGAAAGTTTTCAAACACACCGT 436
H2014700_pW19        TCTTAAAAATGGAT-TTATTAGAATTTTTATAGTAAC---TTTTATATAGAAAGTTTTCAAACACACCGT 435
T3058779_pW19        GTTTCTATATGAAAGTTGCTTAAAAAATCAAATTAATCTATTTTTTTTAAAAAAATT----AGCTAATAC 439
LOC_Os09g25060_pW76  TACTTGACTATATTATCAAACTTGCTCTAACACTGCGGTCTTCAATTTATAAGTAATTAAGTACGGCCCT 439
T3076120_pW76        TACTTGGCTATATTATCAAACTTGCTCTAACACTGCGGTCTTCAATTTATAAGTAATTAAGTACGGCCCT 436
H1974322_pW76p       TACTTGACTATATTATCAAACTTGCTCTAACACTGCGGTCTTCAATTTATAAGTAATTAAGTACGGCCCT 439

                         500       510       520       530       540       550       560
                     ....|....|....|....|....|....|....|....|....|....|....|....|....|....|
LOC_Os01g51690_pW26  TAAAC--CTAGTTAAAT---T---TGAAGTAGTTTGACTTTGACCAAAGTCAAAACATCTTATAACCTGA 518
H289702_pW26         TAAAC--CTAGTTAAAT---T---TGAAGTAGTTTGACTTTGACCAAAGTCAAAACATCTTATAACCTGA 85
T3056296_pW26        TTTTCAGCCAATTAGATGGTT---TAAAGGAACTCAAGCAATTTAAAATTCGAA---TTTCGACCGCTGA 525
LOC_Os01g53260_pW23  TTACGTACCAGTAGAGTACGTACGTATACGTACTGTAACTTTTACAGACAGGAA-CACCTTTTGACTTCG 537
H1961965_pW23        TTACGTACCAGTAGAGTACGTACGTATACGTACTGTAACTTTTACAGACAGGAA-CACCTTTTGACTTCG 537
T3027906_pW23        TTACGTACCAGTAGAGTACGTACGTATACGTACTGTAACTTTTACAGACAGGAA-CACCTTTTGACTTCG 537
LOC_Os06g44010_pW28  TAATG--GCAAATAAATGACTTGTAATATAGAACGGGAGAGTATCTTTTTTTAA---GCTAAGGATCGAG 418
H1976995_pW28        TAATG--GCAAATAAATGACTTGTAATATAGAACGGGAGAGTATCTTTTTTTAA---GCTAAGGATCGAG 511
T3063501_pW28        TAATG--GCAAATAAATGACTTGTAATATAGAACGGGAGAGTATCTTTTTT-AA---GCTAAGGATCGAG 510
LOC_Os05g46020_pW07  ATTTT--CTTAACCGGTTCTTGACTATCCTATCTCCTAGCTTCACCCCCTCCCAAAATTTCTTGGTTTAG 511
H2000561_pW07        ATTTT--CTTAACCGGTTCTTGACTATCCTATCTCCTAGCTTCACCCCCTCCCAAAATTTCTTGGTTTAG 511
T3066720_pW07        AATTT--TTTAACCGGTTCTTGGCTACCCTATCTCCTAGCTTCACCCCCTCCCAAAATTTCTTGGTTTAG 511
H2023318_pW64        TATGAGCTCCATTCAGCATGC----GTGTGGTATAGGAGTTTAATTTAGT-------TTTTATGCGTCAC 363
T3095730_pW64        TATGAGCTCCATTCAGCATGC----GTGTGGTATAGGAGTTTAATTTAGT-------TTTTATGCGTCAC 232
LOC_Os12g02450_pW64  TATGAGCTCCATTCAGCATGC----GTGTGGTA--GGAGTTTAATTTAGT-------TTTTATGCGTCAC 509
LOC_Os01g40260_pW77  -TTTATAA-AAGAGTTTGTCTTTTTTTTTTGGGACGGAGGGAGTAT-AAGCAACTAGGGAAACGTTCCGT 463
T3097007_pW77        -TTTATAA-AAGAGTTTGTCTTTTTTTTTTGG-ACGGAGGGAGTAT-AAGCAACTAGGGAAACGTTCCGT 462
H1950043_pW77        -TTTATAA-AAGAGTTTGTCTTTTTTTTTTGGGACGGAGGGAGTAT-AAGCAACTAGGGAAACGTTCCGT 462
LOC_Os09g25070_pW62  ATATATAGCAAGGATTTGTCTTTTTTGACGCGGAGTTAGTAAGTCTCGAGAGAAGGAAGAAACGT-CCAA 520
H1972014_pW62p       ATATATAGCAAGGATTTGTCTTTTTTGACGCGGAGTTAGTAAGTCTCGAGAGAAGGAAGAAACGT-CCAA 520
T3091546_pW62        ATATATAGCAAGGATTTGTCTTTTTTGACACGGAGTTAGTAAGTCTCGAGAGAAGGAAGAAACGT-CCAA 520
LOC_Os05g39720_pW70  GTATGCAATGTCCATT------GGTAAATAACTCAGAGTATATTCTCCTAAAAATTACTCTATGT-TGTG 499
H334389_pW70         GTATGCAATGTCCATT------GGTAAATAACTCAGAGTATATTCTCCTAAAAATTACTCTATGT-TGTG 499
T3094952_pW70        GTATGTAATGTCCGTT------GGTAAATAACTCAGAGTATATTCTCCTAAAAATTACTCTATGT-TGTG 499
LOC_Os05g45230_pW58  CTATACTACAACGATTACGACGGTTTGATATCTCTCCCTCGTTCGGCGAACAAACCTGCCTGAAC-CCTA 510
H1850565_pW58p       CTATACTACAACGATTACGACGGTTTGATATCTCTCCCTCGTTCGGCGAACAAACCTGCCTGAAC-CCTA 510
T3075919_pW58        CTATACTACAACGATTACGACGGTTTGATATCTCTCCCTCGTTCGGCGAACAAACCTGCCTGAAC-CCTA 498
LOC_Os03g21710_pW79  CTGATTGAAAACTGCAGGTCTCAGAGCACGTAATCTAACGGTCCGTGTGTGTGCCAAGTAAGCGAGTACT 515
H1952278_pW79        CTGATTGAAAACTGCAGGTCTCAGAGCACGTAATCTAACGGTCCGTGTGTGTGCCAAGTAAGCGAGTACT 515
T3070072_pW79        CTGATTGAAAACTGCAGGTCTCAGAGCACGTAATCTAACGGTCCGTGTGTGTGCCAAGTAAGCGAGTACT 515
LOC_Os05g49620_pW19  TTAGTTTGAAAAACGTGCTAACGGAAACCGAGAAAAAAATGTATCTTAATTAATTTAGAACGGGGCCATT 506
H2014700_pW19        TTAGTTTGAAAAACGTGCTAACGGAAACCGAGAAAAAAATGTATCTTAATTAATTTAGAACGGGGCCATT 505
T3058779_pW19        TTAATT--AATCATGTGCTAATGGAACGCTCCG-------TTTTYCGTGCGGAGGCACTGTGTTCCCATC 500
LOC_Os09g25060_pW76  GGTAATTCTCACAATCTCACCTAGCCAATCTCTCC---ACACACCCTTGAGGCCTTGACGTAGACCCCTG 506
T3076120_pW76        GGTAATTCTCACAATCTCACCTAGCCAATCTCTCCTCCACACACCCTTGAGGCCTTGACGTAGACCCCTG 506
H1974322_pW76p       GGTAATTCTCACAATCTCACCTAGCCAATCTCTCC---ACACACCCTTGAGGCCTTGACGTAGACCCCTG 506
```

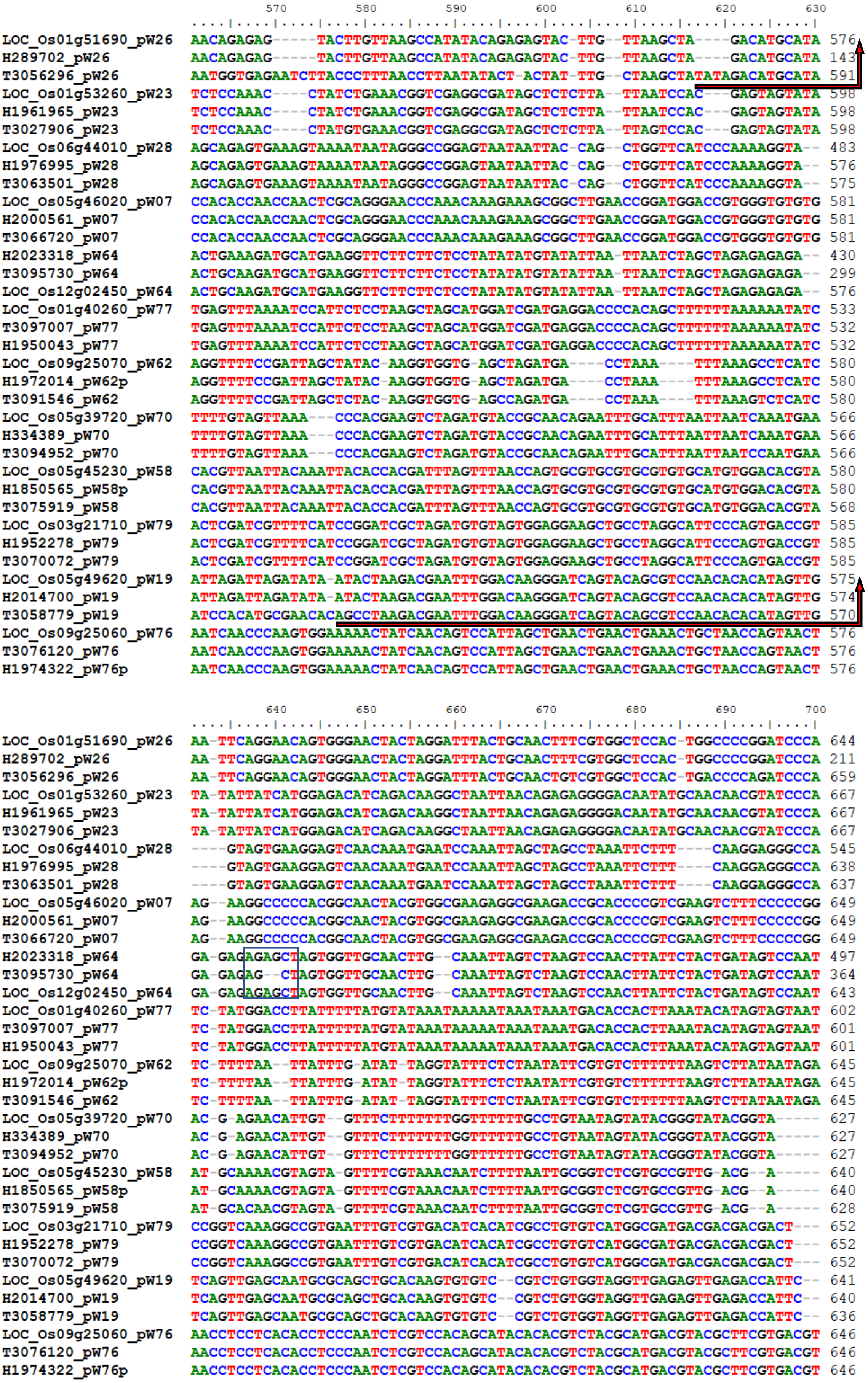

```
                              710       720       730       740       750       760       770
                     ....|....|....|....|....|....|....|....|....|....|....|....|....|....|
LOC_Os01g51690_pW26  TCATCCCAGCCCCACGC-CGCCCCCAAAGTCCAAAACACCTTA--CCACACGAGGGCAAATACCAAAGGC 711
H289702_pW26         TCATCCCAGCCCCACGC-CGCCCCCAAAGTCCAAAACACCTTA--CCACACGAGGGCAAATACCAAAGGC 278
T3056296_pW26        TCATCCCAGCCCCACGC-CGCCCCCAAAGTCCAAAACACCTTA--CCACACGAGGGCAAATACCAAAGGC 726
LOC_Os01g53260_pW23  CAAGCTTCCATCCATGCATGCATGCATGGATCGGCGAAGTTAA--AATTTCTATGACTCGT-TGAGGGAC 734
H1961965_pW23        CAAGCTTCCATCCATGCATGCATGCATGGATCGGCGAAGTTAA--AATTTCTATGACTCGT-TGAGGGAC 734
T3027906_pW23        CAAGCTTCCATCCATGCATGCATGCATGGATCGGCGAAGTTAA--AATTTCTATGACTCGT-TGAGGGAC 734
LOC_Os06g44010_pW28  AATTGTAACCCTCCTGGTTGTTGGCATGTGGTGTTTTTCCTTG--AC-CCTGCTTGCAGATGCAACCAGC 612
H1976995_pW28        AATTGTAACCCTCCTGGTTGTTGGCATGTGGTGTTTTTCCTTG--AC-CCTGCTTGCAGATGCAACCAGC 705
T3063501_pW28        AATTGTAACCCTCCTGGTTGTTGGCATGTGGTGTTTTTCCTTG--AC-CCTGCTTGCAGATGCAACCAGC 704
LOC_Os05g46020_pW07  AGACATTTCCGCCATGC-TGCTAAAATAAGTGGGCACGCCGTG--CG-CGCGTGCGTGCGTTCGAGCGGC 715
H2000561_pW07        AGACATTTCCGCCATGC-TGCTAAAATAAGTGGGCACGCCGTG--CG-CGCGTGCGTGCGTTCGAGCGGC 715
T3066720_pW07        AGACATTTCCGCCATGC-TGCTAAAATAAGTGGGCACGCCGTG--CG-CGCGTGCGTGCGTTCGAGCAGC 715
H2023318_pW64        AAGCATGTGCTACATGCATCATATCAATCCATGAGATCTATTG--ATCTTCCCCTGCAATTGCCTTAGCC 565
T3095730_pW64        AAGCATGTGCTACATGCATCATATCAATCCATGAGATCTATTG--ATCTTCCCCTGCAATTGCCTGAGCC 432
LOC_Os12g02450_pW64  AAGCATGTGCTACATGCATCATATCAATCCATGAGATCTATCG--ATCTTCCCCTGCAGTTGCCTGAGCC 711
LOC_Os01g40260_pW77  TGATTTAAATTTCTGGCTCCGTCTGGACAGGAGCCGGGCCACT-TGTACTGCACAGTTCTGGACTTCTG- 670
T3097007_pW77        TGATTTAAATTTCTGGCTCCGTCTGGACAGGAGCCGGGCCACT-TGTACTGCACAGTTCTGGACTTCTG- 669
H1950043_pW77        TGATTTAAATTTCTGGCTCCGTCTGGACAGGAGCCGGGCCACT-TGTACTGCACAGTTCTGGACTTCTG- 669
LOC_Os09g25070_pW62  AGG--TAAAACATTAATTCCGTAGAAGAAAAAGTAGAATTTTGATATCCTACGAGGCCATGAGTCACAT- 712
H1972014_pW62p       AGG--TAAAACATTAATTCCGTAGAAGAAAAAGTAGAATTTTGATATCCTACGAGGCCATGAGTCACAT- 712
T3091546_pW62        AGG--TAAAAAATTAATTCCGTAGAAGAAAAAGTAGAATTTTGATATCCTACGAGGCCATGAGTCACAT- 712
LOC_Os05g39720_pW70  CGCCCGGAACTAGTCAGACAAACGA--------TTCGCGACCGTCACTCCGCACGCTCCGAACCCCCGC- 688
H334389_pW70         CGCCCGGAACTAGTCAGACAAACGA--------TTCGCGACCGTCACTCCGCACGCTCCGAACCCCCGC- 688
T3094952_pW70        CGCCCGGAACTAGTCAGACAAACGA--------TTCGCGACCGTCACTCCGCACGCTCCGAACCCCCGC- 688
LOC_Os05g45230_pW58  CGCCTTAATCTGCACACGCTTATGGGGAGAGAGAGAGAGAGAGAGAGAGAGAGAGAGAGAGAGATCAGTA 710
H1850565_pW58p       CGCCTTAATCTGCACACGCTTATGGGGAGAGAGAGAGAGAGAGAGAGAGAGAGAGAGAGAGAGATCAGTA 710
T3075919_pW58        CGCCTTAATCTGCACACGCTTACGG--------AGAGAGAGAGAGAGAGAGAGAGAGAGAGAGATCAGTA 690
LOC_Os03g21710_pW79  TAGTTTAG--TATAGTATTAGTACCATATATGTATATATGT----GTAATGTAATCCATGTC-------- 708
H1952278_pW79        TAGTTTAG--TATAGTATTAGTACCATATATGTATATATGT----GTAATGTAATCCATGTC-------- 708
T3070072_pW79        TAGTTTAG--TATAGTATTAGTACCATATATGTATATATGT----GTAATGTAATCCATGTC-------- 708
LOC_Os05g49620_pW19  TATTCTAA--CAAATGGTCAGTCAGACACAAGTTTTTTTTTT-CCGAACGGTCAGTCAGACAGACAGAAG 708
H2014700_pW19        TATTCTAA--CAAATGGTCAGTCAGACACAAGTTTTTTTTTT-CCGAACGGTCAGTCAGACAGACAGAAG 707
T3058779_pW19        TATTCTAA--CAAATGGTCAGTCAGACACAAGTTTTTTTTTTTCCGAACGGTCAGTCAGACAGACAGAAG 704
LOC_Os09g25060_pW76  AAGGCCTCATGCCCTATACGCGATTCGTGACGTGCGCTCCGTGACGTCGTCTTGACCGGGGCGGGACGAC 716
T3076120_pW76        AAGGCCTCATGCCCTATACGCGATTCGTGACGTGCGCTCCGTGACGTCGTCTTGACCGGGGCGGGACGAC 716
H1974322_pW76p       AAGGCCTCATGCCCTATACGCGATTCGTGACGTGCGCTCCGTGACGTCGTCTTGACCGGGGCGGGACGAC 716

                              780       790       800       810       820       830       840
                     ....|....|....|....|....|....|....|....|....|....|....|....|....|....|
LOC_Os01g51690_pW26  AAATAG---GCAAA-AGGCGA-CGTCC---ACGAGTCCATGTCATGA---CACACCCCAAGTTGCTGTAA 770
H289702_pW26         AAATAG---GCAAA-AGGCGA-CGTCC---ACGAGTCCATGTCATGA---CACACCCCAAGTTGCTGTAA 337
T3056296_pW26        AAATAG---GCAAA-AGGCGA-CGTCC---ACGAGTCCATGTCATGA---CACACCCCAAGTTGCTGTAA 785
LOC_Os01g53260_pW23  GAGACG---ACGGGCAGGTGAGCGACC---TTAGATACAGGGTGGTT---GTGATCTGGTTGTGCTCTAT 795
H1961965_pW23        GAGACG---ACGGGCAGGTGAGCGACC---TTAGATACAGGGTGGTT---GTGATCTGGTTGTGCTCTAT 795
T3027906_pW23        GAGACG---ACGGGCAGGTGAGCGACC---TTAGATACAGGGTGGTT---GTGATCTGGTTGTGCTCTAT 795
LOC_Os06g44010_pW28  CAGCCA---GAAGACTCTTCTATCCTC---TTAATTTGACAGAATAATCCCAGATCTAATAAAAAA-CAC 675
H1976995_pW28        CAGCCA---GAAGACTCTTCTATCCTC---TTAATTTGACAGAATAATCCCAGATCTAATAAAAAA-CAC 768
T3063501_pW28        CAGCCA---GAAGACTCTTCTATCCTC---TTAATTTGACAGAATAATCCCAGATCTAATAAAAAAACAC 768
LOC_Os05g46020_pW07  GGCGCGCGCGCGGCAACTCCCGCACCCGCTCCGAACCTTCTCCCTCCTCACGAAGCTCACCGCGAAGCCG 785
H2000561_pW07        GGCGCGCGCGCGGCAACTCCCGCACCCGCTCCGAACCTTCTCCCTCCTCACGAAGCTCACCGCGAAGCCG 785
T3066720_pW07        GGCGCGCGCGCGGCAACTCCCGCACCCGCTCCGAACCTTCTCCCTCCTCACGAAGCTCACCGCGAAGCCG 785
H2023318_pW64        GATTAT---ATTAGGATACAAGTTGTCTTAGCTGAGGTTAAGTGTAGGGCCATATTGAACAGCACTGTAT 632
T3095730_pW64        GATTAT---ATTAGGATACAAGTTGTCTTAGCTGAGGTTAAGTGTAGGGCCATATTGAACAGCACTGTAT 499
LOC_Os12g02450_pW64  GATTAT---ATTAGGGTACAAGTTGTCTTAGCTGAGGT-AATTGTAGGGCCATATTGAACAGCACTGCAT 777
LOC_Os01g40260_pW77  GTTTAGCAGAGACTCGC---CGCGCGCG-CGCGGGTGGCCGTCGCGTTGCCGGCCGTAGCTGTCGCATAT 736
T3097007_pW77        GTTTAGCAGAGACTCGC---CGCGCGCG-CGCGGGTGGCCGTCGCGTTGCCGGCCGTAGCTGTCGCATAT 735
H1950043_pW77        GTTTAGCAGAGACTCGC---CGCGCGCG-CGCGGGTGGCCGTCGCGTTGCCGGCCGTAGCTGTCGCATAT 735
LOC_Os09g25070_pW62  GAGTA--AGACGCTCGGTCACGTAAGCA-CGGGGGCGTCAGCCGCGTCACTG-CTGATGCCAACTTGCTG 778
H1972014_pW62p       GAGTA--AGACGCTCGGTCACGTAAGCA-CGGGGGCGTCAGCCGCGTCACTG-CTGATGCCAACTTGCTG 778
T3091546_pW62        GAGTA--AGACGCTCGGTCACGTAAGCA-CGGGGGCGTCAGCCGCGTCACTG-CTGATGCCAACTTGCTG 778
LOC_Os05g39720_pW70  GACCAGAAACCGTACCCGCCTTCTCCCG-CACCGGGCCCCACCATAGTCCACGTCACCTGCTCAGCGCTC 757
H334389_pW70         GACCAGAAACCGTACCCGCCTTCTCCCG-CACCGGGCCCCACCATAGTCCACGTCACCTGCTCAGCGCTC 757
T3094952_pW70        GACCAGAAACCGTACCCGCCTTCTCCCG-CACCGGGCCCCACCATAGTCCACGTCACCTGCTCAGCGCTC 757
LOC_Os05g45230_pW58  GATCGGCAGTAGTTGAGGTGGTTGATCG-GTCTATGGCTGCTCACTCTTCTC----TCAGCTTGGTGTAC 775
H1850565_pW58p       GATCGGCAGTAGTTGAGGTGGTTGATCG-GTCTATGGCTGCTCACTCTTCTC----TCAGCTTGGTGTAC 775
T3075919_pW58        GATCGGCAGTAGTTGAGGTGGTTGATCG-GTCTATGGCTGCTCACTCTTCTC----TCAGCTTGGTGTAC 755
LOC_Os03g21710_pW79  ---CGGTTTTCTTTGCCTTGCTTTGAT--CAATTCATCTGGATATGTACATCTAGGGCCACAGGGGTCGG 773
H1952278_pW79        ---CGGTTTTCTTTGCCTTGCTTTGAT--CAATTCATCTGGATATGTACATCTAGGGCCACAGGGGTCGG 773
T3070072_pW79        ---CGGTTTTCTTTGCCTTGCTTTGAT--CAATTGATCTGGATATGTACATCTAGGGCCACAGGGGTCGG 773
LOC_Os05g49620_pW19  TAGTGATTAGATTAGAGCTGGATTAGT--CTCTGCACATCTCCATGATGGCCAAGAAACCAAAAGACCGG 776
H2014700_pW19        TAGTGATTAGATTAGAGCTGGATTAGT--CTCTGCACATCTCCATGATGGCCAAGAAACCAAAAGACCGG 775
T3058779_pW19        TAGTGATTAGATTAGAGCTGGATTAGT--CTCTGCACATCTCCATGATGGCCAAGAAACCAAAAGACCGG 772
LOC_Os09g25060_pW76  GCCACGCGCGCGTGGCGCGTCCTTGTCATGGCGGGCGTCGACAGCCAGCTCGCGCGCGCCCCCGTTTTTC 786
T3076120_pW76        GCCACGCGCGCGTGGCGCGTCCGTGTCATGGCGGGCGTCGACAGCCAGCTCGCGCGCGCCCCCGTTTTTC 786
H1974322_pW76p       GCCACGCGCGCGTGGCGCGTCCTTGTCATGGCGGGNNNNNNNNNNNNNNNNNNNNNNNNNNNNNNNNNNN 786
```

```
                          850       860       870       880       890       900       910
                  ....|....|....|....|....|....|....|....|....|....|....|....|....|....|
LOC_Os01g51690_pW26 GTGG----CAGGGCCGTGCGGGGCATCAAAGCAAAACAATTCGAAA-CGGGGCGGCTCGCG-CACGGTCG 834
H289702_pW26        GTGG----CAGGGCCGTGCGGGGCATCAAAGCAAAACAATTCGAAA-CGGGGCGGCTCGCG-CACGGTCG 401
T3056296_pW26       GTGG----CACGGCCGCGCGGGGCATCAAAGCAAAACAATTCGAAA-CGGGGCGGCTCGCG-CACGGTCG 849
LOC_Os01g53260_pW23 CTCT----CGCCCTCTCTCCAATCTCCATCTCT---CTGTCTGATT-TGTAATCTTTTCC--CTTGGCTT 855
H1961965_pW23       CTCT----CGCCCTCTCTCCAATCTCCATCTCT---CTGTCTGATT-TGTAATCTTTTCC--CTTGGCTT 855
T3027906_pW23       CTCT----CGCCCTCTCTCCAATCTCCATCTCT---CTGTCTGATT-TGTAATCTTTTCC--CTTGGCTT 855
LOC_Os06g44010_pW28 AGTT----TAATCCTCCTTATTTTCTAAAATGAAAATAGTAGTACT-ACTACTAATTATCATCACAAATG 740
H1976995_pW28       AGTT----TAATCCTCCTTATTTTCTAAAATGAAAATAGTAGTACT-ACTACTAATTATCATCACAAATG 833
T3063501_pW28       AGTT----TAATCCTCCTTATTTTCTAAAATGAAAATAGTAGTACT-ACTACTAATTATCATCACAAATG 833
LOC_Os05g46020_pW07 GCAA----AATCCTCCCGGAGAGAGAGACACAAACACAACACAACC-AGC--TCCCCCGCGCCCCGCGTG 848
H2000561_pW07       GCAA----AATCCTCCCGGAGAGAGAGACACAAACACAACACAACC-AGC--TCCCCCGCGCCCCGCGTG 848
T3066720_pW07       GCAA----AATCCTCCCGGAGAGAGAGACACAAACACAACACAACC-AGC--TCCCCCGCGCCCCGCGCG 848
H2023318_pW64       ACAC----CATCATCGCGTGTAT----ATATAGCCACATTAAAGTA-GGCACCAATCAAGTACACACTTC 693
T3095730_pW64       ACAC----CATCATCGCGTGTAT----ATATAGCCACATTAAAGTA-GGCACCAATCAAGTACACACTTC 560
LOC_Os12g02450_pW64 ACACGCACCATCATCCTGCATATC-TCGTTTAATTGTGTATATATATAGCCACATTAAAGTACACACTTG 846
LOC_Os01g40260_pW77 CCGGGATCGAGACGTGCGTGTATGT-GCTACTGCTGCATGCGGCGATGTTTCCTGCCGCGATTTTCCAGG 805
T3097007_pW77       CCGGGATCGAGACGTGCGTGTATGT-GCTACTGCTGCATGCGGCGATGTTTCCTGCCGCGATTTTCCAGG 804
H1950043_pW77       CCGGGATCGAGACGTGCGTGTATGT-GCTACTGCTGCATGCGGCGATGTTTCCTGCCGCGATTTTCCAGG 804
LOC_Os09g25070_pW62 TCGTAGTGAGCACAATCGGTCAAGTCAATGGGGGAGAATGCCAAAATCTCCTTTGTGGGCTTAATTTATA 848
H1972014_pW62p      TCGTAGTGAGCACAATCGGTCAAGTCAATGGGGGAGAATGCCAAAATCTCCTTTGTGGGCTTAATTTATA 848
T3091546_pW62       TCGTAGTGAGCACAATCGGTCAAGTCAATGGAGGAGAATGCCAAAATCTCCTTTGTGGGCTTAATTTATA 848
LOC_Os05g39720_pW70 TCCCA-TTGGTGGGTGCGAATA-GT--CCACAGCAGCATGCGGGGTCGCAGCCCGCCAGTCAAACC---- 819
H334389_pW70        TCCCA-TTGGTGGGTGCGAATA-GT--CCACAGCAGCATGCGGGGTCGCAGCCCGCCAGTCAAACC---- 819
T3094952_pW70       TCCCA-TTGGTGGGTGCGAATA-GT--CCACAGCAGCATGCGGGGTCGCAGCCCGCCAGTCAAACC---- 819
LOC_Os05g45230_pW58 ACAGT-TTGAGACGCGGGAGAG-GT--G-AAGGCAGCTTGCACGCTTGCCCACCCCCACCTTCCCCGC-- 838
H1850565_pW58p      ACAGT-TTGAGACGCGGGAGAG-GT--G-AAGGCAGCTTGCACGCTTGCCCACCCCCACCTTCCCCGC-- 838
T3075919_pW58       GCAGT-TTGAGACGCGGGAGAG-GT--G-AAGGCAGCTTGCACGCTTGCCCACCCCCACCTTCCCCGC-- 818
LOC_Os03g21710_pW79 TCGGGAACCGGGACCCACCTCCGCTCTTCCATGCAGCTTCCTCATTT-TAGCCTTTTTCCGAGTTCCGAC 842
H1952278_pW79       TCGGGAACCGGGACCCACCTCCGCTCTTCCATGCAGCTTCCTCATTT-TAGCCTTTTTCCGAGTTCCGAC 842
T3070072_pW79       TCGGGAACCGGGACCCACCTCCGCTCTTCCATGCAGCTTCCTCATTT-TAGCCTTTTTCCGAGTTCCGAC 842
LOC_Os05g49620_pW19 TCCAAGTGTTTGAATGACACTCGGGTAGTGATATTCTCTGCTTAGTCATAATCTTTTCCCTCGGTCCGGT 846
H2014700_pW19       TCCAAGTGTTTGAATGACACTCGGGTAGTGATATTCTCTGCTTAGTCATAATCTTTTCCCTCGGTCCGGT 845
T3058779_pW19       TCCAAGTGTTTGAATGACACTCGGGTAGTGATATTCTCTGCTTAGTCATAATCTTTTCCCTCGGTCCGGT 842
LOC_Os09g25060_pW76 CCACT-CAATTGCGTTACAGTTTTCTCCCACAGATCCATGCAAATTAAAGATCACACGGTCTCGTTCTCG 855
T3076120_pW76       CCACT-CAATTGCGTTACAGTTTTCTCCCACAGATCCATGCAAGTTAAAGATCACACGGTCTCGTTCTCG 855
H1974322_pW76p      NNNNN-NNNNNNNNNNNNNNNNNNNNCCCACAGATCCATGCAAATTAAAGATCACACGGTCTCGTTCTCG 855

                          920       930       940       950       960       970       980
                  ....|....|....|....|....|....|....|....|....|....|....|....|....|....|
LOC_Os01g51690_pW26 CGCGCGCCGAAGCTTCATCCAACGACTCGAGCCAGGAAGCTCGGTGCGAAGCCCA-CCGACGTCGCCCGC 903
H289702_pW26        CGCGCGCCGAAGCTTCATCCAACGACTCGAGCCAGGAAGCTCGGTGCGAAGCCCA-CCGACGTCGCCCGC 470
T3056296_pW26       CGCGCGCCGAAGCTTCATCCAACGACTCGAGCCAGGAAGCTCGGTGCGAAGCCCA-CCGACGTCGCCCGC 918
LOC_Os01g53260_pW23 TGAGCGTGCACGCTT-GCCCAACTTCCC---CTTGGCTTCTAGCGTCTTATTCCA-CACGCGCACTCCAG 920
H1961965_pW23       TGAGCGTGCACGCTT-GCCCAACTTCCC---CTTGGCTTCTAGCGTCTTATTCCA-CACGCGCACTCCAG 920
T3027906_pW23       TGAGCGTGCACGCTT-GCCCAACTTCCC---CTTGGCTTCTAGCGTCTTATTCCA-CACGCGCACTCCAG 920
LOC_Os06g44010_pW28 ACATGATTGATGCTACCTTTAGTCTTTAGACCCTAATTATCCTCCACATGCTGAAATGAAAACCACACTG 810
H1976995_pW28       ACATGATTGATGCTACCTTTAGTCTTTAGACCCTAATTATCCTCCACATGCTGAAATGAAAACCACACTG 903
T3063501_pW28       ACATGATTGATGCTACCTTTAGTCTTTAGACCCTAATTATCCTCCACATGCTGAAATGAAAACCACACTG 903
LOC_Os05g46020_pW07 CGATCGATCACGATCGATCTCATCCCTCTCGATCGCCTACTACTACCAGTACAAATCCACCCCGCCACCA 918
H2000561_pW07       CGATCGATCACGATCGATCTCATCCCTCTCGATCGCCTACTACTACCAGTACAAATCCACCCCGCCACCA 918
T3066720_pW07       CGATCGATCACGATCGATCTCATCCCTCTCGATCGCCTACTACTACCAGTACAAATCCACCCCGCCACCA 918
H2023318_pW64       TGACAAACCTGGTTAACCACACTGCCACAAAGCCAGGTGATCGTCTC---CCTTAATTAGCTTCCACTAA 760
T3095730_pW64       TGACAAACCTGGTTAACCACACTGCCACAAAGCCAGGTGATCGTCTC---CCTTAATTAGCTTCCACTAA 627
LOC_Os12g02450_pW64 TGACAAACCTGATTAACCACACTGCCACAAAGCCAGGTGATCATCTC---CCTTAATCAGCTTCCATTAA 913
LOC_Os01g40260_pW77 CAAAGTCCCCCCGCGCACACCC-ACACTGGCACATGTACTACGTC-GCCGTCGGTTTGAACGCGGCCGTG 873
T3097007_pW77       CAAAGTCCCCCCGCGCACACCC-ACACTGGCACATGTACTACGTC-GCCGTCGGTTTGAACGCGGCCGTG 872
H1950043_pW77       CAAAGTCCCCCCGCGCACACCC-ACACTGGCACATGTACTACGTC-GCCGTCGGTTTGAACGCGGCCGTG 872
LOC_Os09g25070_pW62 TGTGGATCACCATTATCCTCGC-ACAA-AGTTCACGTTTTGGTCC-CCCTCCTGTTCGGCGACGGGCGAC 915
H1972014_pW62p      TGTGGATCACCATTATCCTCGC-ACAA-AGTTCACGTTTTGGTCC-CCCTCCTGTTCGGCGACGGGCGAC 915
T3091546_pW62       TGTGGATCACCATTATCCTCGC-ACAA-AGTTCACGTTTTGGTCC-CCCTCCTGTTCGGCGACGGGCGAC 915
LOC_Os05g39720_pW70 TCGAGGGAGCTTTGACCAGTCA-ACGG----TCAAACGTTCAAAG-GTCTATATAATGATCACCGGAGGC 883
H334389_pW70        TCGAGGGAGCTTTGACCAGTCA-ACGG----TCAAACGTTCAAAG-GTCTATATAATGATCACCGGAGGC 883
T3094952_pW70       TCGAGGGAGCTTTGACCAGTCA-ACGG----TCAAACGTTCAAAG-GTCTATATAATGATCACCGGAGGC 883
LOC_Os05g45230_pW58 TCACACCGCCCTTCACCT-TCC-ACTT----CCAGTCGCAGCTAA-CCCT-CGCAGCTGCACCTGCACAC 900
H1850565_pW58p      TCACACCGCCCTTCACCT-TCC-ACTT----CCAGTCGCAGCTAA-CCCT-CGCAGCTGCACCTGCACAC 900
T3075919_pW58       TCACACCGCCCTTCACCT-TCC-ACTT----CCAGTCGCAGCTAA-CCCT-CGCAGCTGCACCTGCACAC 880
LOC_Os03g21710_pW79 TGCTTTTCGGCTTTTTTCCCCG-GCGATATATAAGACGCGGATTGGCACGGCGAATTTTGCTTCATTTGC 911
H1952278_pW79       TGCTTTTCGGCTTTTTTCCCCG-GCGATATATAAGACGCGGATTGGCACGGCGAATTTTGCTTCATTTGC 911
T3070072_pW79       TGCTTTTCGGCTTTTTTCCCCG-GCGATATATAAGACGCGGATTGGCACGGCGAATTTTGCTTCATTTGC 911
LOC_Os05g49620_pW19 CAAAGTTTGGATCTGAACATGACGCAACGAATCACATTTTGCT---CCCTATAAATACTCCTCTCCCTG- 912
H2014700_pW19       CAAAGTTTGGATCTGAACATGACGCAACGAATCACATTTTGCT---CCCTATAAATACTCCTCTCCCTG- 911
T3058779_pW19       CAAAGTTTGGATCTGAACATGACGCAACGAATCACATTTTGCT---CCCTATAAATACTCCTCTCCCTG- 908
LOC_Os09g25060_pW76 CCGCCTATATGTAGCGCCACCGATTCC----CCATGAACCATTCAAGTGTTTGTACTTGGCTTTGCTGGC 921
T3076120_pW76       CCGCCTATATGTAGCGCCACCGATTCC----CCATGAACCATTCAAGTGTTTGTACTTGGCTTTGCTGGC 921
H1974322_pW76p      CCGCCTATATGTAGCGCCACCGATTCC----CCATGAACCATTCAAGTGTTTGTACTTGGCTTTGCTGGC 921
```

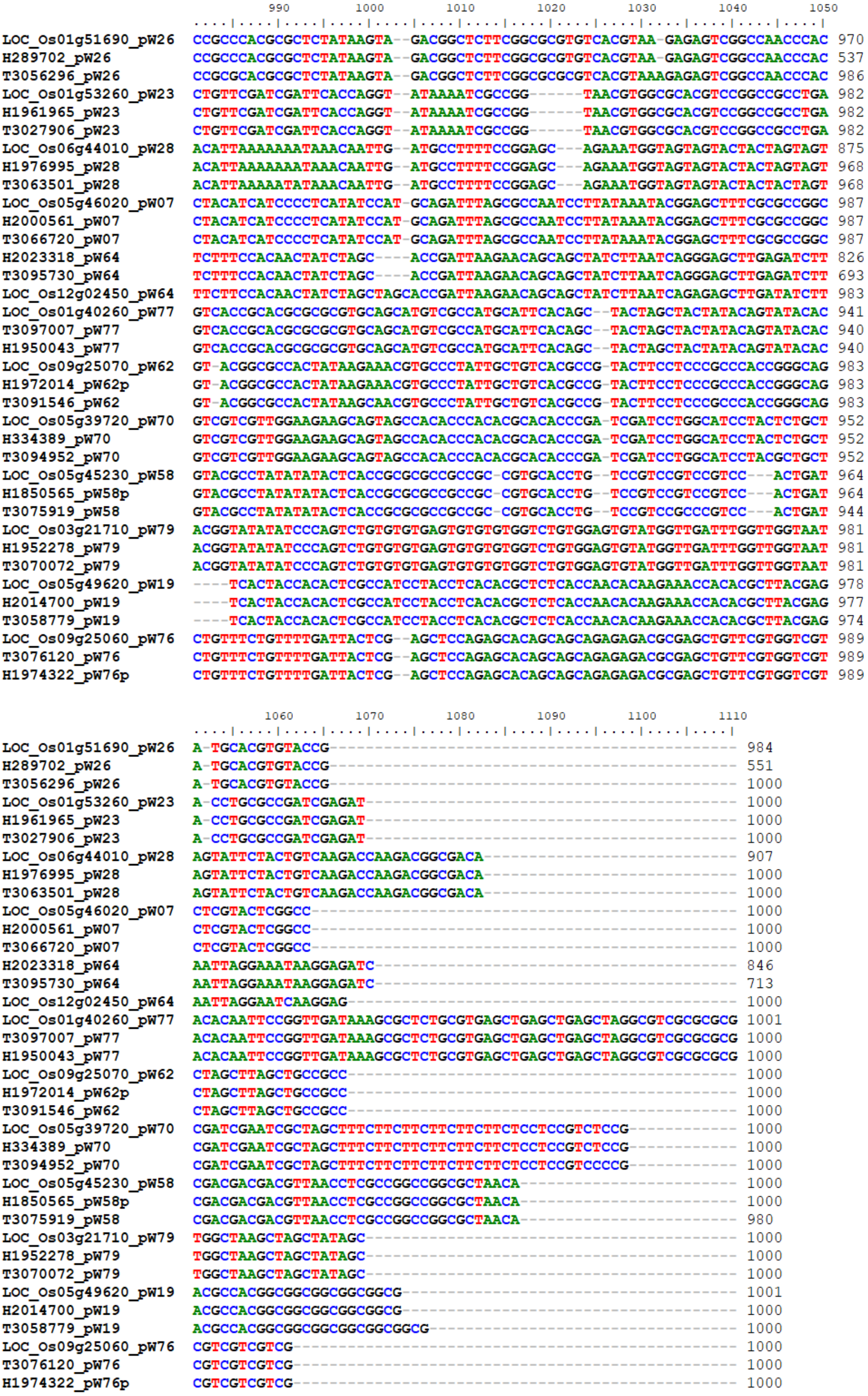

Fig. S4: Structural variation at promoter regions in Tetep and HP2216 in comparison with Nipponbare. The black boxes are showing variations and the brown arrows showing sequence dissimilarity.

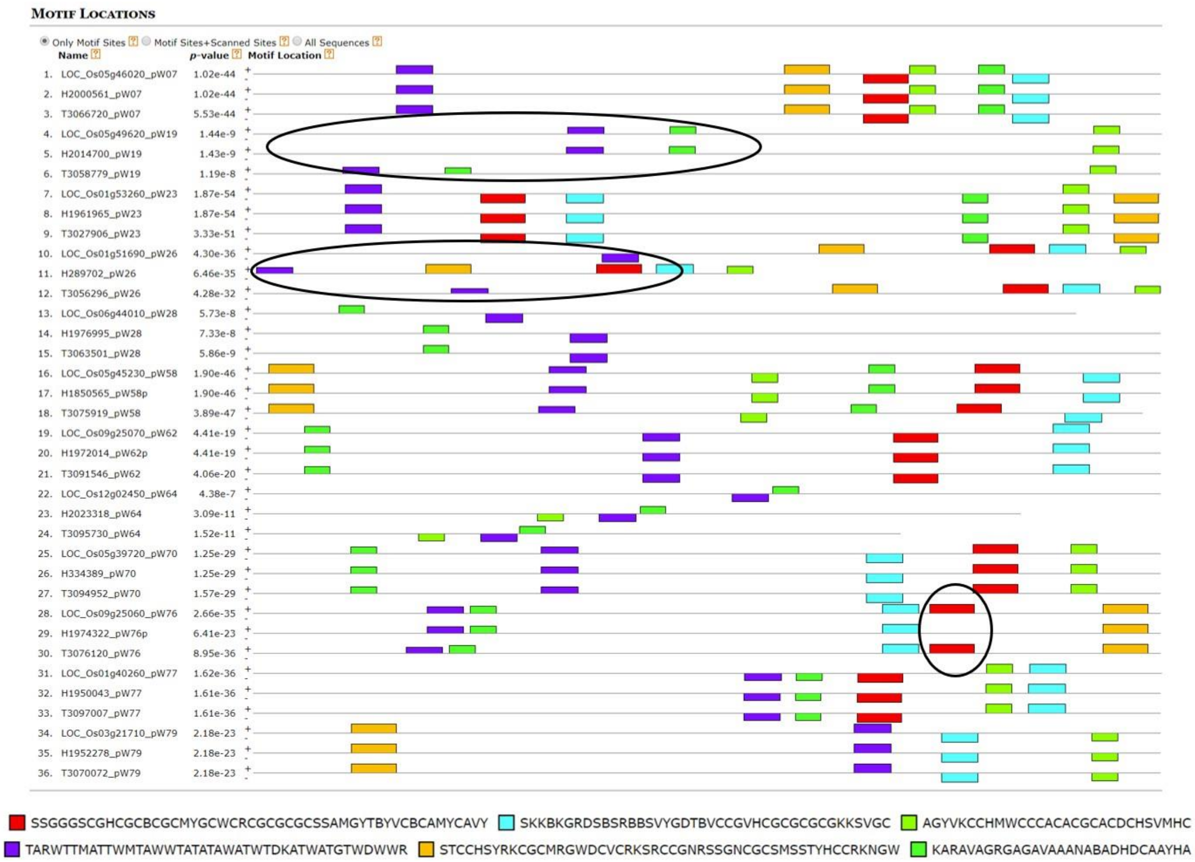

Fig. S5: 1 kb promoter analysis of DEG WRKY for conserved domains search using MEME software. Black circles depict variations in domains.
